# Marine biodiversity loss in Finnish coastal waters: Evidence and implications for management

**DOI:** 10.1007/s13280-025-02185-x

**Published:** 2025-05-16

**Authors:** Henri Sumelius, Samuli Korpinen, Alf Norkko, Sonja Salovius-Laurén, Markku Viitasalo, Christoffer Boström

**Affiliations:** 1 Environmental and Marine Biology, Åbo Akademi University, Aurum, Henriksgatan 2, 20500 Turku, Finland; 2 Finnish Environment Institute Syke, Latokartanonkaari 11, 00790 Helsinki, Finland; 3 Tvärminne Zoological Station, University of Helsinki, J.A. Palméns Väg 260, 10900 Hanko, Finland

**Keywords:** Baltic Sea, Biodiversity loss, Climate change, Eutrophication, Littoral, Management and conservation

## Abstract

Marine biodiversity loss poses significant ecological and socio-economic challenges. This paper examines how coastal biodiversity loss is expressed and outlines implications for management. Synthesizing scientific literature on biodiversity change in the well-studied Finnish coastal waters (Baltic Sea) as a case study, we show that biodiversity loss occurs throughout the area in virtually all biotopes and organism groups examined. Biodiversity loss was expressed in 43 different ways. The three most common forms of biodiversity loss—local disappearance of species and decrease in abundance and biomass—covered nearly half of the observations. For these, the most common underlying causes were eutrophication, climate change, and physical disturbance of the seabed. Overall, eutrophication and climate change were the most frequent ones among the 13 loss drivers identified. We emphasize that critical knowledge gaps must be bridged, and monitoring improved, but, importantly, resolute decisions for action are required for the recovery of coastal marine ecosystems.

**Supplementary Information:**

The online version contains supplementary material available at 10.1007/s13280-025-02185-x.

## Introduction

Biodiversity loss is a global threat caused by human activity (CBD 2020). Habitats are degrading or disappearing, populations of animals and plants are in decline, and species are going extinct—nature is changing faster than ever before in human history (IPBES 2019). Biodiversity loss is estimated to be strongest and most variable in the marine realm (Blowes et al. 2019). However, compared to terrestrial habitats, marine environments are not easily accessible, making observation of biodiversity changes less obvious (Paasche and Bonsdorff 2018). In this paper, we synthesize key findings from the scientific literature on coastal marine biodiversity loss in a northern Baltic Sea case study area and provide perspectives on ways forward to counteract this negative trend.

Globally, habitat alteration, resource exploitation, and pollution are primary drivers of biodiversity loss (IPBES 2019). Additionally, climate change effects are already apparent and are projected to further accelerate this decline (Pörtner et al. 2023). Only 13% of all marine areas are free from intense effects of human activities, and there are very few unexploited coastal areas (Jones et al. 2018). The coastal zone maintains high biodiversity and productivity but faces simultaneously strong pressures from human activities both from land and at sea. Marine biodiversity loss in coastal areas inevitably also affects ecosystem services (e.g., fish production; Worm et al. 2006), many of which support human well-being and socio-economic development (Heckwolf et al. 2021). As both pressures and nature values converge in this limited space, the coastal zone is particularly important for management efforts (Lotze et al. 2006). A better understanding of marine biodiversity—and its loss—helps us appreciate and protect it (Barbier et al. 2011).

The Baltic Sea is one of the most rapidly changing seas in the world (Reusch et al. 2018). It is a northern, semi-enclosed, non-tidal, sea with a mix of marine, freshwater and brackish water ecosystems (Snoeijs-Leijonmalm et al. 2017). Key environmental factors, such as seabed characteristics, salinity, nutrient levels, light availability, and temperature, vary considerably across the sea (Leppäranta and Myrberg 2009) affecting biodiversity patterns from south to north and west to east (HELCOM 2020). The Baltic Sea is highly affected by various types of human activities, with eutrophication being overall the most significant long-lasting human-induced pressure (HELCOM 2023a). Regional biodiversity indicators, covering pelagic and benthic environments and their biota including algae, invertebrates, fish, water birds and marine mammals, show generally poor status across the Baltic Sea and in all levels of the food web, with only a few indicators having acceptable levels in certain parts of the sea (HELCOM 2023b). However, to our knowledge there are no comprehensive syntheses of biodiversity loss in the Baltic Sea as a whole or, indeed, for the coastal areas that are hotspots of biodiversity.

Most Baltic Sea coastal countries are committed to the targets of UN’s Kunming-Montreal Global Biodiversity Framework (CBD 2022) and EU Biodiversity Strategy (EC 2020) aiming to halt and reverse the trend of biodiversity loss. At a regional level, efforts toward these goals are based on the Helsinki Convention on the Protection of the Marine Environment of the Baltic Sea Area (HELCOM 2014) and the Baltic Sea Action Plan (HELCOM 2021). However, so far, actions to halt biodiversity loss and improve the state of the Baltic Sea have not been sufficient.

**Fig. 1:**
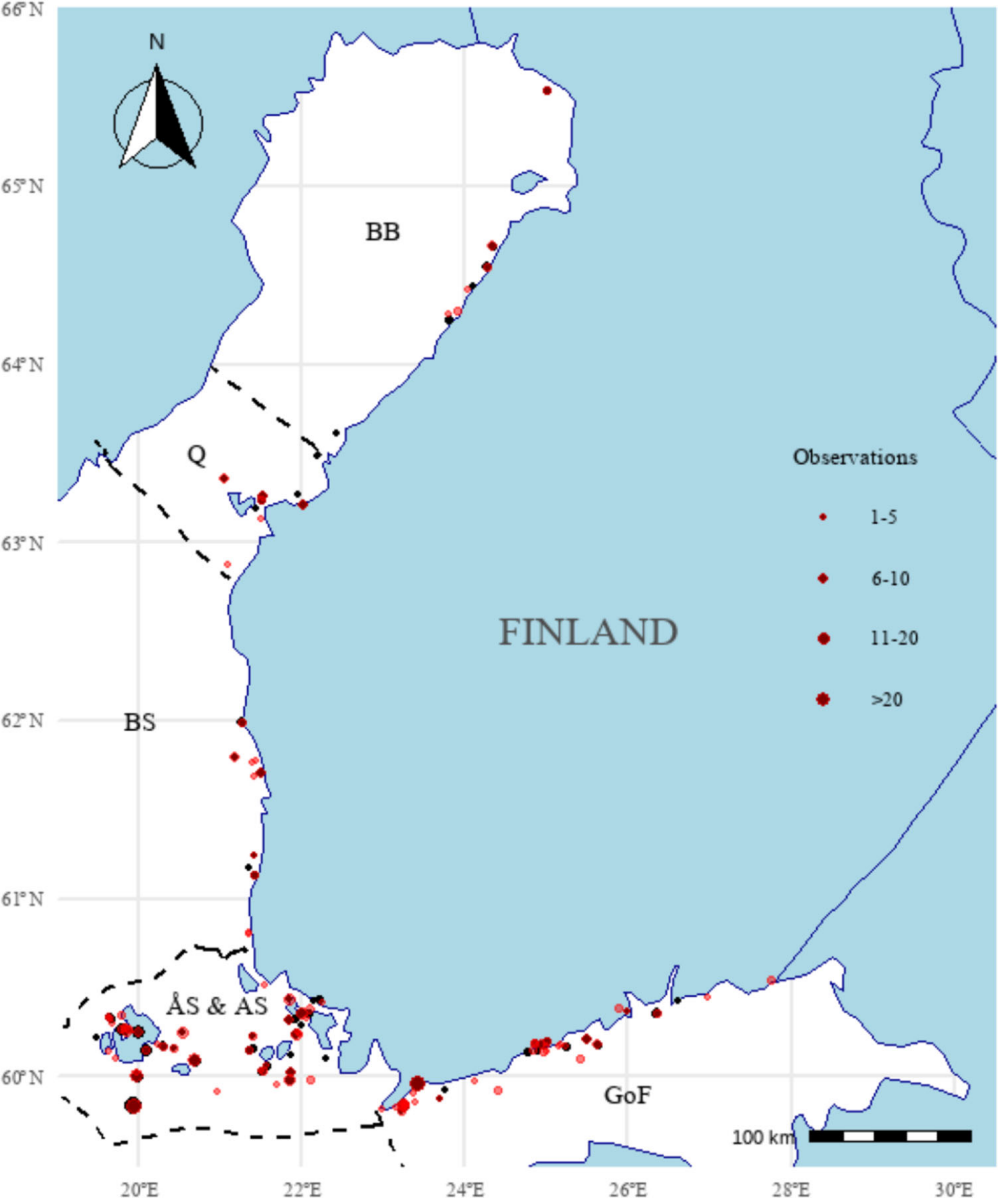
A map of the case study area, the marine coastal areas of Finland in the northern Baltic Sea. Research observations related to potential biodiversity change in the shallow (0–10 m) littoral waters as found from the scientific literature are marked with different sized dots corresponding to the number of observations from each geographical location. Red dots show the observations indicating biodiversity loss (i.e., negative changes), while the black dots correspond to non-negative changes in biodiversity. Dashed lines indicate the borders for the following Finnish sea areas: BB = Bothnian Bay, Q = Quark, BS = Bothnian Sea, ÅS & AS = sea area of the Åland Islands and the Archipelago Sea (treated separately in the assessment), GoF = Gulf of Finland. To be noted that the purpose of the map is to give a robust visual overview of the research observations, but not to give exact georeferences to each location as some of the displayed locations are, e.g., approximations for multi-site observations. Source: R (basemap layer) and HELCOM (sea area borders)

In this paper we use the sea areas of Finland (Fig. 1), the northern Baltic Sea, as a case study to assess changes in the coastal marine biodiversity. Finland has a vast coastline of around 46 000 km and one of the most complex archipelagos in the world (Viitasalo et al. 2017). Due to the low salinity (~ 0–6), general shallowness, and shoreline fragmentation, Finland’s coastal waters support a unique set of underwater biotopes and biodiversity (Snoeijs-Leijonmalm et al. 2017). While the overall number of macroscopic species along the Finnish coast is relatively low compared to more saline sea areas, some groups such as fish and aquatic plants are more diverse than in the southern Baltic Sea due to the increasing presence of freshwater species toward the north (HELCOM 2020). Only few of the biodiversity indicators in Finland’s Marine Strategy (based on the EU Marine Strategy Framework Directive, MSFD) show good environmental status in all Finnish marine areas. Importantly, no single area is in good state according to all indicators (SYKE 2024). In addition, around 5% of assessed marine species and about a quarter of underwater biotopes along the Finnish coast are classified as threatened (Kontula and Raunio 2018; Hyvärinen et al. 2019). Although Finland boasts one of the most comprehensive underwater marine biodiversity inventory programs and datasets globally (Forsblom et al. 2024), the temporal changes in shallow (< 10 m) coastal ecosystems are still understood only on a very generic level.

Here we summarize evidence from the peer-reviewed literature to synthesize our current understanding of biodiversity loss, how it is expressed and where it occurs in biodiversity- and management-wise important shallow sublittoral ecosystems along the Finnish coast. We also identify drivers of change and main knowledge gaps. Finally, we offer perspectives on why this information is important and how it can inform future research, management and conservation. Our results show that biodiversity loss affects virtually all shallow underwater biotopes and organism groups across all coastal areas in Finland (Table 1). Coastal biodiversity loss is a diverse threat, comprising much more than local extinctions and population declines.

**Table 1:**
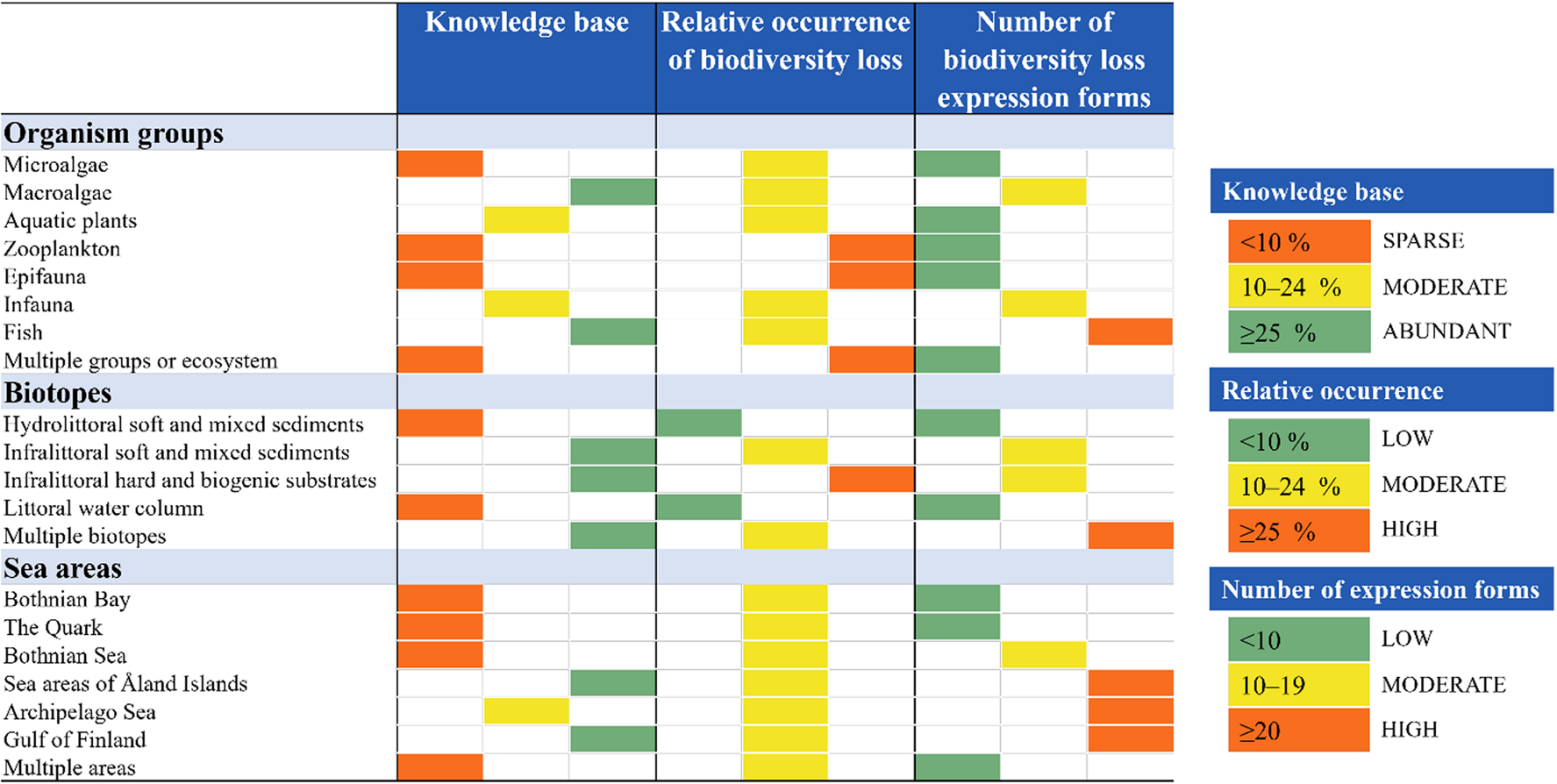
Summary of the manifestation of underwater biodiversity loss in shallow coastal areas based on available research literature, categorized by organism groups, EUNIS level 3 classified Baltic Sea biotopes, and sea areas of the Finnish coast. “Knowledge base” refers to the quantity of data (as research observations) relevant for biodiversity change, derived from scientific articles included in the literature analysis, and is classified as sparse, moderate, or abundant. “Relative occurrence of biodiversity loss” refers to the proportion of biodiversity loss evidence within all included data for each organism group, biotope, or sea area grouping, categorized as low, moderate, or high. “Number of biodiversity expression forms” refers to the number of different expression forms of biodiversity loss observed in the scientific literature, classified as low, moderate, or high. Definitions and boundaries for these classifications are explained in detail in the Supplementary Information, Sect. 3)

## Methods overview

We systematically searched (Web of Science and Scopus databases, 10/2022) the peer-reviewed literature for empirical studies on temporal biodiversity changes in the coastal waters of Finland. To target the important land-sea interface in focus of the assessment, we concentrated on the shallowest (0–10 m depth), euphotic, sublittoral zone. All major underwater organism groups were included, except mammals, reptiles, and waterfowl. Data that showed or could have shown temporal changes in marine biodiversity were considered eligible (i.e., data of negative, positive or no changes; Box 1).

Biodiversity loss was defined as a negative change at any level (genotypic to ecosystem and functions) of biodiversity over time. As the definability of ‘negative’ is inherently subjective, it was here determined on linguistic grounds^1^—i.e., based on the type of change word used (e.g., decline, reduction, disappearance, decrease, weakening)—rather than on the ecological significance of the change. For biodiversity we applied the definition by the Intergovernmental Science-Policy Platform on Biodiversity and Ecosystem Services (IPBES 2019).

The literature search rendered 3513 unique records that underwent a relevance screening according to set eligibility criteria (Box 1). Finally, 90 articles (published between 1984–2023) were included for further assessment. From each article one or more discernable research observations related to changes in biodiversity were extracted, constituting the dataset of this assessment. In total 774 research observations were identified, of which 427 (55%) indicated biodiversity loss and the rest (347) showed positive or no changes. Based on the level of biodiversity concerned and the observed type of change, we identified different ways for biodiversity loss to be expressed in (hereafter ‘expression forms’; see examples in Box 1). The research observations were assessed with respect to these expression forms of biodiversity loss and its occurrence across organism groups, biotopes, and geographical areas. The data included are presented in three ways: 1) as the quantity of all included research observations related to biodiversity change, 2) as the quantity of research observations indicating biodiversity loss, and 3) as the relative occurrence of biodiversity loss. The quantity of all included research observations (1) and the quantity of biodiversity loss observations (2) are presented as the category-wise percent shares across organism groups, biotopes, and geographical areas. For the relative occurrence of biodiversity loss (3) a different percentage is used, i.e., a percent share of observations indicating biodiversity loss relative to all included observations for each assessable categorization of interest. (N_biodiversity loss observation in a category_ * N^–1^_all observations in a category_ = ‘relative occurrence’, %).

Research observations indicating biodiversity loss were linked to one or more drivers of change as provided in the literature. Only a small portion (8%) of the driver linkages were evidence-based (i.e., the impact of a particular driver had been explicitly studied), while the rest (92%) were classified as probable or possible. The significance of the drivers was assessed as their frequency of occurrence (%) among the observations indicating biodiversity loss. Each driver could attain an occurrence frequency between 0 and 100%. These drivers are presented and discussed in the dedicated chapter below in varying level of detail reflecting their significance based on the literature.

A detailed account of the literature search, bibliographic results, assessment methods, and supplemental results are provided in a previous report (Sumelius and Boström 2024) and in the Supplementary Information of this article.

Box 1. Eligibility criteria of the literature search

- **Temporal:** The observation period must be at least 4 years long, with no predefined start year. No temporal limitations related to publication year were defined for the search.
- **Spatial:** All marine areas belonging to Finland (the northern Baltic Sea) and their shallow coastal areas, defined as underwater areas from the surface to the bottom, from the shallowest sublittoral zone (> 0 m) to the lower boundary of the euphotic zone (approximately 10 m).
- **Organism groups:** All aquatic organisms except for waterfowl, mammals, and reptiles.
- **Changes in biodiversity and factors of biodiversity loss:** Biodiversity changes are broadly interpreted as any temporal changes in any element of biodiversity and biodiversity loss encompasses all such negative changes. Based on the level of biodiversity concerned and the observed type of negative change, biodiversity loss expression forms were identified, including but not limited to, e.g., local decline or disappearance of species or populations, decrease in species richness or other negative changes (e.g., homogenization) in communities, declines in population abundance and biomass, negative changes in species occurrence and distribution, degradation concerning individual traits in species or populations, altered or degraded ecological functions, or other community or ecosystem-level changes that due to their complexity are difficult to clearly define as negative or positive.
- **Data:** Primarily field study data derived from temporal observations, sampling, measurements, and monitoring conducted in nature, excluding pure experimental studies or future scenario modeling. Temporal trend studies or time point comparisons, with such designs and data structures that could indicate changes in biodiversity metrics, indicators, or any other elements of biodiversity. Both quantitative and qualitative results were accepted.

## Evidence of biodiversity loss

### The expression of biodiversity loss is diverse

**Fig. 2:**
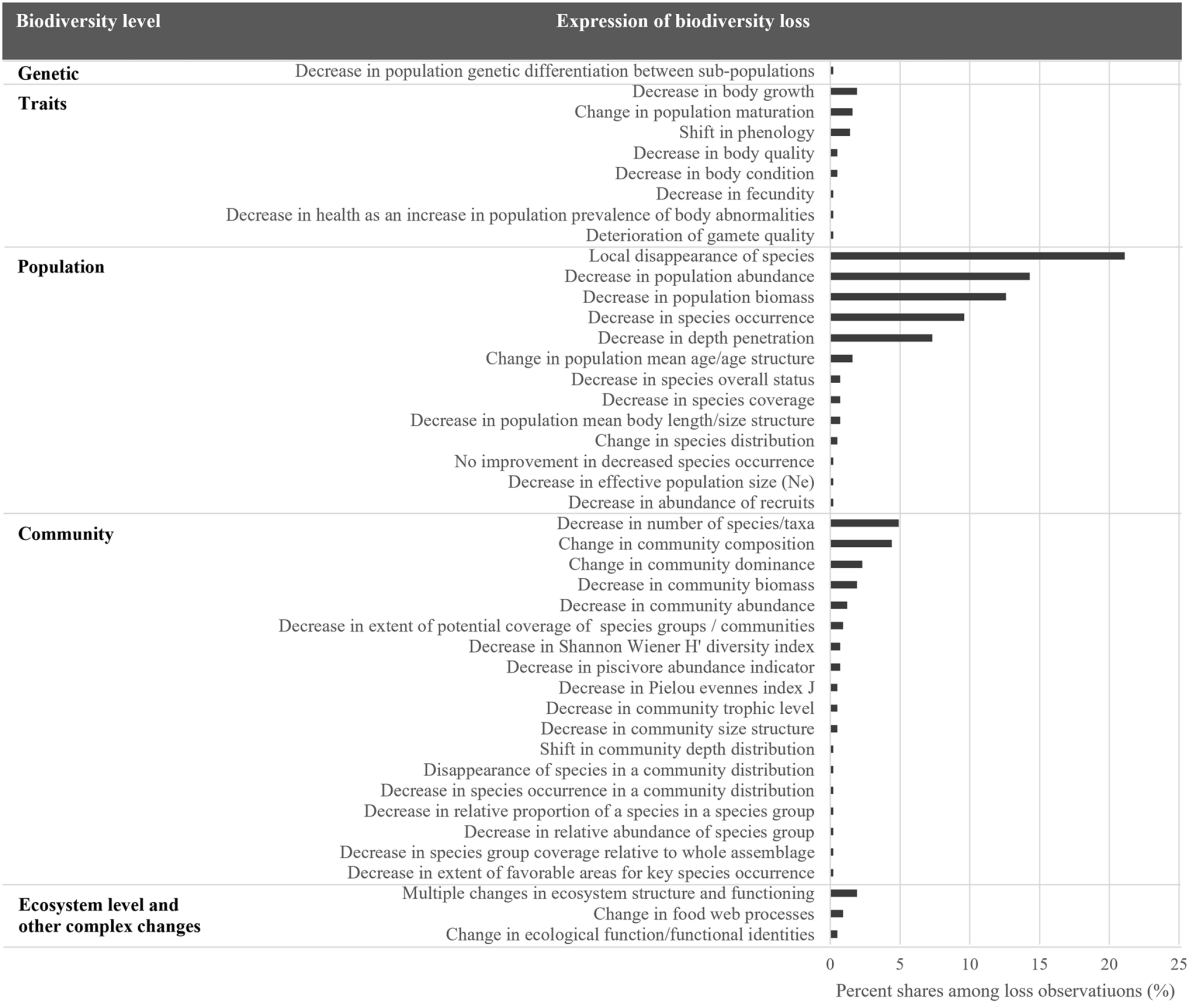
The percent shares of all found expression forms of biodiversity loss among the research observations indicating loss (n = 427). The data is categorized by different levels of biodiversity

Forty-three different expression forms of biodiversity loss were identified in the literature. The five most common expression forms covered 65% of all observations and were: local disappearance of species (21%), decrease in population abundance (14%), decrease in population biomass (13%), reduction in species occurrence (10%), and narrowing of distributional depth range (7%; Fig. 2). Expression forms related to genetic diversity and species traits were the rarest and occurred exclusively in fish. The most diverse set of biodiversity loss (18 forms) was observed at the community level (Fig. 2).

Biodiversity loss was most diverse in fish (27 forms), while in most other organism groups it occurred in fewer than ten ways (Table 1). Among the sublittoral biotopes identified, biodiversity loss was most diverse (13 forms) in Infralittoral Muddy Sediments. Geographically, fewer forms of biodiversity loss were found in the Gulf of Bothnia (9–11 forms) compared to the Archipelago Sea, the Gulf of Finland, and the Åland Islands (> 20 forms; Table 1). Noteworthily, there was a correlation (Pearson’s *r* = 0.85–0.87, p < 0.001) between the number of expression forms and the number of research observations (Fig. S5). This could potentially explain the observed geographical and organism group specific differences. A summary and literature examples of the expression of biodiversity loss are given in Table 2.

**Table 2:** Summary and examples of the expression of biodiversity loss by Finnish sea areas and littoral underwater biotopes (EUNIS level 3). The numbers shown in parentheses represent the number of research observations. *The reference information for the literature is presented in full in the Supplementary Information, Table S3

Sea area/biotope	Biodiversity loss expression form	Species/taxon/ecological levels	Example	Literature*
Gulf of Finland (123)				
Littoral Water Column (4)	• Decrease in population biomass in phytoplankton (1) • Change in community composition in phytoplankton (2) • Change in community composition in zooplankton (1)	*Peridinella catenata* (1), Phytoplankton community (2), Zooplankton community (1)	As a probable consequence of eutrophication and the local temperature increase from a power plant, the abundance of cyanobacteria (Cyanophyceae) in the phytoplankton community, particularly in late summer and autumn, increased by approximately 61% during the monitoring period from 1971 to 1994 in Loviisa, in the eastern Gulf of Finland, to the extent that cyanobacteria locally dominated the biomass of the phytoplankton community (Ilus and Keskitalo 2008).	Finni et al. (2001); Ilus and Keskitalo (2008)
Hydrolittoral Muddy Sediments (4)	• Decrease in species occurrence in aquatic plants (3) • Local disappearance of species in aquatic plants (1)	*Alisma plantago-aquatica* (1)*, Typha angustifolia* (1)*, Typha latifolia* (1)*, Ranunculus confervoides* (1)	*Ranunculus confervoides* disappeared from the archipelago of the western Gulf of Finland between the years 1936–1948 and 2005–2007, as a probable consequence of eutrophication, physical disturbance, and secondary effects from ecological interactions (Pitkänen et al. 2013).	Pitkänen et al. (2013)
Hydrolittoral Mixed Sediments (1)	• Decrease in species occurrence in aquatic plants (1)	*Schoenoplectus* sp. (1)	The occurrences of *Schoenoplectus* sp. decreased by approximately 76% in the archipelago of the western Gulf of Finland between the years 1936–48 and 2005–2007, as a probable consequence of eutrophication, physical disturbance, and secondary effects from ecological interactions (Pitkänen et al. 2013).	Pitkänen et al. (2013)
Infralittoral Muddy Sediments (26)	• Decrease in species occurrence in aquatic plants (9) • Local disappearance of species in aquatic plants (1) • Change in distribution of aquatic plants (1) • Decrease in population abundance in benthic infauna (2) • Local disappearance of species in benthic infauna (8) • Decrease in number of species/taxa in benthic infauna (1) • Change in community composition in benthic infauna (1) • Decrease in community abundance in benthic infauna (1) • Decrease in population abundance in fish (1) • Decrease in species occurrence in fish (1)	*Potamogeton pusillus* (1), *Chara globularis* (1*), Chara tomentosa* (2) *Equisetum fluviatile* (1), *Nitella* sp. (1), *Nymphaea alba* (1), *Persicaria amphibia* (1), *Ranunculus peltatus* ssp. *baudotii* (1), *Sagittaria sagittifolia* (1), *Schoenoplectus maritimus* (1), *Asellus asellus* (1), *Corophium voluntator* (1), *Gammarus locusta* (1), *Monoporeia affinis* (1), *Prostoma obscurum* (1), *Saduria entomon* (1), Trichoptera (1), *Macoma baltica* (1), Chironomidae (1), Oligochaeta (1), Benthic infauna community (3), *Gymnocephalus cernuus* (1), *Rutilus rutilus* (1)	At a sampling site in the western Gulf of Finland, the benthic invertebrate community changed between the reference years 1928 and 2000 from a previously diverse community dominated by *Corophium volutator*, *Macoma baltica*, and Chironomidae to a poorer community dominated by *M. baltica* and the newly arrived polychaete, *Marenzelleria viridis*, probably due to eutrophication related environmental changes. Up to 11 species, including all crustacean species, had disappeared, and only 3 species were present in both 1928 and 2000 (Laine et al. 2003).	Lehtonen et al. (1998); Laine et al. (2003); Munsterhjelm et al. (2008) and Pitkänen et al. (2013)
Infralittoral Sandy Sediments (23)	• Decrease in species occurrence in aquatic plants (6) • Local disappearance of species in aquatic plants (4) • Decrease in population abundance in benthic infauna (4) • Decrease in species occurrence in benthic infauna (1) Decrease in number of species/taxa in benthic infauna (1) • Change in community dominance in benthic infauna (1) • Decrease in diversity index (H’) in benthic infauna community (2) • Decrease in evenness index (Pielou J) in benthic infauna community (2) • Decrease in population abundance in fish (2)	*Eleocharis acicularis* (1), *Eleocharis parvula* (1), *Isoëtes lacustris* (1), *Plantago uniflora* (1), *Ranunculus reptans* (1), *Stuckenia filiformis* (1), *Chara baltica* (1), *Isoëtes echinospora* (1), *Myriophyllum alterniflorum* (1), *Potamogeton gramineus* (1), *Corophium volutator* (2), *Nereis diversicolor* (1), *Pygospio elegans* (2), Benthic infauna community (6), *Platichthys* sp. (2)	In the western Gulf of Finland, the juvenile densities of flounder (*Platichthys* sp.) in shallow coastal areas decreased dramatically by approximately 98% between the comparison periods of 1979–1992 and 2012–2014, probably linked to eutrophication related reduced habitat quality (Jokinen et al. 2016).	Boström et al. (2002); Jokinen et al. (2015, 2016); Pitkänen et al. (2013)
Infralittoral Mixed Sediments (11)	• Local disappearance of species in aquatic plants (4) • Change in community composition in benthic infauna (1) • Decrease in body growth in fish (2) • Decrease in population abundance in fish (2) • Decrease in population biomass in fish (1) • Change in community composition in fish (1)	*Elatine hydropiper* (1), *Elatine triandra* (1), *Potamogeton alpinus* (1), *Potamogeton gramineus x perfoliatus* (1), Epifauna community (1), *Perca fluviatilis* (1), *Rutilus rultilus* (1), *Platichthys* sp. (2), *Blicca bjoerkna* (1), Fish community (1)	*Potamogeton gramineus* disappeared from the archipelago of the western Gulf of Finland between the years 1936–1948 and 2005–2007, as a probable consequence of eutrophication, physical disturbance, and secondary effects from ecological interactions (Pitkänen et al. 2013).	Lappalainen and Pesonen (2000); Lappalainen et al. (2001); Packalén et al. (2008); Jokinen et al. (2015); Pitkänen et al. (2013)
Infralittoral Biogenic Habitats (9)	• Decrease in population abundance in epifauna (6) • Decrease in population biomass in epifauna (3)	*Mytilus trossulus* (9)	The number/density of blue mussel recruits decreased by approximately 83% from the peak values in 1998 to the year 2005 in the mid-coastal area of the Gulf of Finland, as a result of temporal changes in key environmental variables linked to climate change (Westerbom et al. 2019).	Westerbom et al. (2019)
Infralittoral Rock (15)	• Decrease in species occurrence in macroalgae (3) • Decrease in species depth penetration in macroalgae (7) • Local disappearance of species in macroalgae (3) • Decrease in population abundance in fish (1) • Decrease in population biomass in fish (1)	*Fucus vesiculosus* (2), *Fucus* spp. (3), *Coccotylus truncatus/Phyllophora pseudoceranoides* (1*), Furcellaria lumbricalis* (2), *Vertebrata fucoides* (1), *Rhodomela confervoides* (1), *Ahnfeltia plicata* (1), *Chroodactylon ornatum* (1), *Erythrocladia polystromatica* (1), *Platichthys* sp. (2)	The occurrence depth of bladderwrack (*Fucus* spp.) in the western Gulf of Finland decreased by approximately 52% (from about 8 m to 4.5–5 m) between the reference years 1980 and 2000, as a probable consequence of eutrophication via increased sedimentation and decreased water transparency (Rinne and Salovius-Laurén 2020).	Kangas et al. (1985); Torn et al. (2006); Jokinen et al. (2015); Rinne and Salovius-Laurén (2020); Rinne and Kostamo (2022)
Multiple Biotopes (30)	• Decrease in number of species/taxa in diatoms (4) • Change in community composition in diatoms (1) • Change in community dominance in diatoms (5) • Decrease in diversity index (H’) in diatom community (2) • Decrease in relative abundance of species group in diatoms (1) • Decrease in species occurrence in aquatic plants (1) • Local disappearance of species in aquatic plants (1) • Decrease in body condition in fish (1) • Shift in phenology in fish (1) • Change in population mean body length/size structure in fish (1) • Decrease in population abundance in fish (2) • Decrease in population biomass in fish (8) • Decrease in piscivore abundance indicator in fish community (1) • Decrease in relative proportion of a species in a species group (1) • Multiple changes in ecosystem structure and functioning (1)	*Bacillariophyceae* (12), Aquatic plant community (2), *Platichthys* sp. (6), *Perca fluviatilis* (1), *Esox lucius* (2), *Clupea harengus membras* (2), *Salmo trutta* (1), *Lota lota* (1), *Osmerus eperlanus* (1), Fish community (1), Littoral ecosystem (1)	Test fishing data (1989–2013) indicate a significant and sudden decline in flounder (*Platichthys* sp.) in the western Gulf of Finland, with the average numerical catch per unit effort decreasing by approximately 97% in the years following 2003 compared to the years before, potentially linked to multiple ecosystem changes including declines in salinity during critical phases of reproduction and habitat change and loss due to eutrophication effects (Jokinen et al. 2015).	Korhola and Blom (1996); Lehtonen et al. (1998); Lehtonen et al. (2009); Lappalainen et al. (2001); Boström et al. (2002); Rönnberg and Bonsdorff (2004); Weckström (2006); Lehikoinen et al. (2011); Pitkänen et al. (2013); Jokinen et al. (2015); Bergström et al. (2016); Momigliano et al. (2019); Weigel et al. (2021); Peltonen and Weigel (2022); Olsson et al. (2023)
Archipelago Sea (82)				
Infralittoral Muddy Sediments (24)	• Decrease in population abundance in benthic infauna (4) • Decrease in population biomass in benthic infauna (1) • Local disappearance of species in benthic infauna (10) • Decrease in number of species/taxa in benthic infauna (3) • Change in community composition in benthic infauna (2) • Decrease in community abundance in benthic infauna (2) • Decrease in community biomass in benthic infauna (2)	*Mytilus edulis* (1), *Bithynia tentaculata* (1), Ceratopogonidea (1), *Halicryptus spinulosus* (1) *Harmothoe sarsi* (1), *Lymnea peregra* (1), *Monoporeia affinis* (1), *Mya arenaria* (1), *Polydora redeki* (1), *Prostoma obscurum* (1), *Macoma bathica* (2), *Nereis diversicolor* (1), Chironomidae (1), *Hydrobia* sp. (1), Benthic infauna community (9)	The population biomass of *Macoma balthica* decreased by approximately 98% in a sheltered bay in the Archipelago Sea between 1965 and 2005, likely due to both local fish farming and the general eutrophication trend in the Baltic Sea (Holmström et al. 2007).	Bonsdorff et al. (1997); Kraufvelin et al. (2001); Holmström et al. (2007)
Infralittoral Mixed Sediments (13)	• Decrease in population abundance in fish (2) • Decrease in species occurrence in fish (6) • Local disappearance of species in fish (3) • Decrease in number of species/taxa in fish (1) • Decrease in community biomass in fish (1)	*Gasterosteus aculeatus* (1), *Gobius niger* (1), *Nerophis ophidion* (1), *Perca fluviatilis* (1), *Pomatoschistus minutus* (1), *Pungitius pungitius* (1), *Gobiusculus flavescens* (1), *Phoxinus phoxinus* (1), *Zoarces viviparus* (1), Fish community (2)	The total biomass of the littoral fish community collapsed by approximately 99% under the effects of eutrophication in the Archipelago Sea between 1980 and 1996 (Rajasilta et al. 1999).	Rajsilta et al. (1999)
Infralittoral Rock (18)	• Change in species distribution in macroalgae (1) • Decrease in species depth penetration in macroalgae (6) • Decrease in species coverage in macroalgae (2) • Decrease in population abundance in macroalgae (1) • Decrease in species occurrence in macroalgae (4) • Local disappearance of species in macroalgae (3) • No improvement in decreased species occurrence in macroalgae (1)	*Fucus vesiculosus* (7), *Fucus* spp. (2), *Coccotylus truncatus/Phyllophora pseudoceranoides* (2), *Furcellaria lumbricalis* (2), *Vertebrata fucoides* (1), *Rhodomela confervoides* (1), *Ahnfeltia plicata* (1), *Chroodactylon ornatum* (1), *Erythrocladia polystromatica* (1)	As a result of eutrophication effects and decreased salinity, the abundance (density within the bladderwrack zone) of bladderwrack decreased by approximately 85% in the outer archipelago of the southern Archipelago Sea from 1993 to 2001 and has remained low since then (up to 2007) (Vahteri and Vuorinen 2016).	Rönnberg et al. (1985); Snickars et al. (2014); Torn et al. (2006); Vahteri and Vuorinen (2016); Rinne and Salovius-Laurén (2020); Rinne and Kostamo (2022)
Multiple Biotopes (27)	• Decrease in number of species/taxa in diatoms (1) • Shift in phenology in fish (1) • Decrease in fecundity in fish (1) • Decrease in body growth in fish (1) • Decrease in body condition in fish (1) • Decrease in body quality in fish (2) • Decrease in health in fish (1) • Change in maturation in fish (1) • Deterioration of gamete quality in fish (1) • Change in population mean body length/size structure in fish (1) • Decrease in abundance of recruits in fish (1) • Decrease in population biomass in fish (8) • Decrease in population abundance in fish (2) • Change in community composition in fish (1) • Decrease in piscivore abundance indicator in fish community (1) • Multiple changes in ecosystem structure and function (3)	Bacillariophyceae (1), *Clupea harengus membras* (10), *Sander lucioperca* (2), *Perca fluviatilis* (2), *Esox lucius* (2), *Lota lota* (1), *Platichthys* sp. (3), *Salmo trutta* (1), Fish community (2), Littoral ecosystem (3)	The species richness of diatoms decreased significantly (by approximately 27%) due to increased nutrient point loading from urbanization from the late 1800s until 1950, after which no clear improvement has occurred (Weckström et al. 2007).	Kääriä et al. (1988); Bonsdorff et al. (1997); Leppäkoski et al. (1999); Rajasilta et al. (1999); Rajasilta et al. (2016); Rajasilta et al. (2019); Rajasilta et al. (2021); Rönnberg and Bonsdorff (2004); Ådjers et al. (2006); Weckström et al. (2007); Jokinen et al. (2015); Kokkonen et al. (2015); Kokkonen et al. (2019); Bergström et al. (2016); Weigel et al. (2021); Peltonen and Weigel (2022); Olsson et al. (2023)
Åland Islands (137)				
Infralittoral Muddy Sediments (22)	• Local disappearance of species in benthic infauna (6) • Decrease in community abundance in benthic infauna (2) • Decrease in community biomass in benthic infauna (5) • Decrease in number of species/taxa in benthic infauna (1) • Change in community composition in benthic infauna (5) • Change in ecological function / functional identities in benthic infauna (2) • Change in community dominance in fish (1)	*Bylgides sarsi* (1), *Cerastoderma glaucum* (1), *Corophium voluntator* (1), *Jaera albifrons* (1), *Saduria entomon* (1), *Theodoxus fluviatilis* (1), Benthic infauna community (15), Fish community (1)	The total individual abundances of benthic invertebrates in the archipelago of the Åland Islands (57 sampling sites) decreased by approximately 25% between 1989 and 2000, potentially due to changed nutrient availability and system productivity (Perus and Bonsdorff 2004).	Blomqvist (1984); Perus and Bonsdorff (2004); Villnäs et al. (2011); Weigel et al. (2015, 2016)
Infralittoral Sandy Sediments (1)	• Decrease in population abundance in fish (1)	*Platichthys* sp. (1)	In the archipelago of the Åland Islands, the juvenile densities of flounder (*Platichthys* spp.) in shallow coastal areas decreased by approximately 68% between the comparison periods of 1980–1992 and 2012–2014, probably linked to eutrophication related reduced habitat quality (Jokinen et al. 2016).	Jokinen et al. (2016)
Infralittoral Mixed Sediments (6)	• Decrease in population abundance in aquatic plants (4) • Local disappearance of species in aquatic plants (2)	*Chara aspera* (1), *Potamogeton pectinatus* (2), *Potamogeton perfoliatus* (2), *Tolypella nidifica* (1)	In the middle archipelago zone of the Åland Islands, *Potamogeton pectinatus* had locally decreased by approximately 84% in one area and completely disappeared from another between 1975 and 2000, likely due to changes in substrate quality caused by eutrophication (Roos et al. 2004).	Roos et al. (2004)
Infralittoral Rock (81)	• Decrease in population abundance in macroalgae (21) • Decrease in species occurrence in macroalgae (3) • Decrease in species coverage in macroalgae (1) • Local disappearance of species in macroalgae (39) • Decrease in species depth penetration in macroalgae (9) • Decrease in number of species/taxa in macroalgae (7) • Decrease in species group coverage relative to whole assemblage in macroalgae (1)	*Fucus vesiculosus* (9), *Fucus* spp. (1), *Furcellaria lumbricalis* (5), *Rhodomela confervoides* (4), *Gaillona rosea* (2), *Ahnfeltia plicata* (1), *Grania efflorescens* (2), *Battersia plumigera* (1), *Capsosiphon fulvescens* (2), *Chaetomorpha linum* (2), *Chaetomorpha sp.* (2), *Chroodactylon ornatum* (1), *Coccotylus truncatus/Phyllophora pseudoceranoides* (6), *Dictyosiphon chordaria* (1), *Dictyosiphon foeniculaceus* (1), *Enteromorpha sp.* (2), *Erythrocladia polystromatica* (1), *Eudesme virescens* (2), *Halopteris scoparia* (2), *Litosiphon laminariae* (1), *Polysiphonia fibrillosa* (3), *Vertebrata fucoides* (3), *Pseudolithoderma rosenvingei* (1), *Rhodomela confervoides* (3), *Rhodochorton purpureum* (1), *Sphacelaria plumigera* (1), *Sphacelaria arctica* (1), *Sphacelaria* spp. (1), *Streblonema oligosporum* (1), *Urospora penicilliformis* (1), *Ceramium tenuicorne* (2), *Chorda filum* (2), *Ectocarpus silicosus* (2), *Pilayella littoralis* (1), *Stictyosiphon tortilis* (2), *Ulva intestinalis* (1), *Cladophora rupestris* (1), *Spongomorpha aeruginosa* (1), Macroalgal community (8)	The depth distribution of bladderwrack (*Fucus* sp.) in the southeastern Åland Islands has decreased dramatically (by approximately 90%) since the 1950s compared to the levels during the period 2004–2016, as a probable consequence of eutrophication via increased sedimentation and decreased water transparency (Rinne and Salovius-Laurén 2020).	Rönnberg et al. (1985); Rönnberg and Mathiessen (1998); Roos et al. (2004); Torn et al. (2006); Eveleens Maarse et al. (2020); Rinne and Salovius-Laurén (2020); Sahla et al. (2020); Rinne and Kostamo (2022)
Multiple Biotopes (27)	• Decrease in number of species/taxa in benthic infauna (1) • Shift in phenology in fish (1) • Decrease in population abundance in fish (3) • Decrease in population biomass in fish (5) • Decrease in community size structure in fish (2) • Decrease in community trophic level in fish (2) • Change in community dominance in fish (3) • Change in community composition in fish (1) • Decrease in piscivore abundance indicator in fish community (1) • Shift in community depth distribution in fish (1) • Multiple changes in ecosystem structure and function (7)	Benthic infauna community (1), *Clupea harengus membras* (2), *Esox lucius* (1), *Lota lota* (1), *Platichthys* sp. (2), *Rutilus rutilus* (1), *Sander lucioperca* (2), *Salmo trutta* (1), Fish community (9), Littoral ecosystem (7)	In two bay areas in the Åland Islands, the fish community changed between 2000 and 2009, with the proportion of pikeperch decreasing by approximately 39%, while the proportion of cyprinids increased during the same period, probably caused by effects of eutrophication in combination with fishing pressure (Mustamäki and Mattila 2015).	Blomqvist (1984); Bonsdorff et al. (1992, 1997); Leppäkoski et al. (1999); Rönnberg and Bonsdorff (2004); Ådjers et al. (2006); Mustamäki et al. (2014); Jokinen et al. (2015); Mustamäki and Mattila (2015); Snickars et al. (2015); Yletyinen et al. (2015); Bergström et al. (2016); Weigel et al. (2021); Peltonen and Weigel (2022); Olsson et al. (2023)
Bothnian Sea (25)				
Littoral Water Column (1)	• Change in community composition in microalgae (1)	Phytoplankton community (1)	In the coastal area of the Bothnian Sea, the planktonic microalgal community experienced an increase in eutrophication-favoring species (including many cyanobacteria) at the expense of species not favoring eutrophication between 1977 and 1982 (Keskitalo 1987).	Keskitalo (1987)
Infralittoral Muddy Sediments (3)	• Decrease in population abundance in benthic infauna (1) • Decrease in number of species/taxa in benthic infauna (1) • Change in community composition in benthic infauna (1)	*Pontoporeia affinis* (1), Benthic infauna community (2)	The average number of benthic infauna species at a monitoring site in the Bothnian Sea (Rauma) decreased by approximately 17% between 1973 and 1982, probably linked to eutrophication and worsened water quality. After this period, the situation stabilized, and the species richness began a slight recovery (Mattila 1993).	Mattila (1993)
Infralittoral Sandy Sediments (1)	• Decrease in population abundance in fish (1)	*Coregonus lavaretus* (1)	In the shallow sandy bottoms of the Bothnian Sea, the numbers of young whitefish (*Coregonus lavaretus*) decreased by 50% between the reference years of 1990 and 2010, as a potential consequence of eutrophication effects and more frequent iceless winters (Veneranta et al. 2013).	Veneranta et al. (2013)
Infralittoral Rock (9)	• Decrease in species depth penetration in macroalgae (4) • Decrease in species occurrence in macroalgae (2) • Local disappearance of species in macroalgae (3)	*Fucus* spp. (1), *Coccotylus truncatus/Phyllophora pseudoceranoides* (1*), Furcellaria lumbricalis* (2), *Vertebrata fucoides* (1), *Rhodomela confervoides* (1), *Ahnfeltia plicata* (1), *Chroodactylon ornatum* (1), *Erythrocladia polystromatica* (1)	The depth distribution of bladderwrack (*Fucus* spp.) in the Bothnian Sea has significantly decreased (by approximately 64%) since 1950 compared to the period from 2004 to 2016, as a probable consequence of eutrophication via increased sedimentation and decreased water transparency (Rinne and Salovius-Laurén 2020).	Rinne and Salovius-Laurén (2020); Sahla et al. (2020); Rinne and Kostamo (2022)
Multiple Biotopes (11)	• Shift in phenology in fish (1) • Decrease in body growth in fish (2) • Change in maturation in fish (2) • Decrease in population biomass in fish (5) • Decrease in species overall status in fish (1)	*Platichthys* sp. (1), *Clupea harengus membras* (2), *Coregonus spp.* (5), *Salmo trutta* (2), *Lota lota* (1)	The majority of Baltic Sea sea trout populations have deteriorated as a probable consequence of degradation of spawning and rearing rivers and exploitation by fishery in the sea; most historical populations have become extinct, and the remaining populations are threatened by small reproductive sizes (Jutila et al. 2007).	Jutila et al. (2007); Veneranta et al. (2021); Weigel (2021); Peltonen and Weigel (2022)
The Quark (24)				
Infralittoral Rock (8)	• Decrease in species depth penetration in macroalgae (3) • Local disappearance of species in macroalgae (3) • Decrease in species occurrence in macroalgae (2)	*Furcellaria lumbricalis* (2), *Rhodomela confervoides* (1), *Ahnfeltia plicata* (1), *Chroodactylon ornatum* (1), *Erythrocladia polystromatica* (1), *Coccotylus truncatus/Phyllophora pseudoceranoides* (1*), Vertebrata fucoides* (1)	The occurrence of *Furcellaria lumbricalis* red algae in the Bothnian Sea decreased from the 1960s to 2015, as a probable consequence of eutrophication via reduced light penetration, increased sedimentation, and increased competition from filamentous green and brown algae, and of negative physiological and reproductive changes due to decreasing salinity (Rinne and Kostamo 2022).	Rinne and Kostamo (2020)
Multiple Biotopes (16)	• Shift in phenology in fish (1) • Change in maturation in fish (2) • Decrease in population biomass in fish (9) • Decrease in population abundance in fish (1) • Decrease in species overall status in fish (1)	*Clupea harengus membras* (2), *Coregonus* spp. (5), *Salmo trutta* (2), *Abramis brama* (1), *Esox lucius* (2), *Lota lota* (2), *Perca fluviatilis* (1), *Osmerus eperlanus* (1)	Based on official catch and sales statistics from commercial fishing, the bream (*Abramis brama*) stock collapsed (approximately 93% decrease in catch) from the levels of 1977–1979 to the levels of 1987–1988, due to episodical local acidification (Hudd and Leskelä 1998).	Kjellman and Hudd (1996); Hudd and Leskelä (1998); Jutila et al. (2007); Veneranta et al. (2021); Weigel et al. (2021); Peltonen and Weigel (2022)
Bothnian Bay (21)				
Multiple Biotopes (21)	• Decrease in genetic differentiation between sub-populations in fish (1) • Shift in phenology in fish (1) • Decrease in body growth in fish (1) • Change in maturation in fish (2) • Change in population mean body length/size structure in fish (1) • Change in population mean age / age structure in fish (7) • Decrease in population biomass in fish (6) • Decrease in species overall status in fish (1) • Decrease in effective population size (Ne) in fish (1)	*Clupea harengus membras* (1), *Coregonus* sp. (3), *Coregonus lavaretus* (13), *Coregonus maraena* (1), *Salmo trutta* (2), *Coregonus albula* (1)	On the coast of the Bay of Bothnia (Kalajoki estuary), the growth rate of vendace (*Coregonus lavaretus*) slowed down between the comparison periods of 1984–1989 and 1990–2001, probably linked to heavy fishery exploitation. As a result, the average lengths of males and females at ages 5 and 6 significantly decreased during the study period (Aronsuu and Huhmarniemi 2004).	Lehtonen et al. (1995); Aronsuu and Huhmarniemi (2004); Jutila et al. (2007); McCairns et al. (2012); Lappalainen et al. (2020); Veneranta et al. (2021); Weigel et al. (2021); Peltonen and Weigel (2022)
Multiple Areas (15)				
Infralittoral Muddy Sediments (4)	• Decrease in extent of potential coverage of species groups / communities in macroalgae (1) • Decrease in extent of potential coverage of species groups / communities in aquatic plants (2) • Decrease in extent of potential coverage of species groups / communities in benthic infauna (1)	Macroalgal community (1), Aquatic plant community (1), Benthic infauna community (1)	The occurrences and coverage of potential aquatic plants (including vascular plants, stoneworts, and aquatic mosses) on shallow soft bottoms have decreased due to dredging activities in coastal areas of Finland (Virtanen et al. 2023).	Virtanen et al. (2023)
Infralittoral Rock (3)	• Decrease in species depth penetration in macroalgae (2) • Decrease in extent of favorable areas for key species occurrence in macroalgae (1)	*Fucus* spp. (2), Macroalgal community (1)	The reduction in light availability has decreased environments favorable to bladderwrack-dominated macroalgal communities by 45% from 1905 to 2005 and by 31% over a 50-year period from 1955 to 2005 (Sahla et al. 2020).	Sahla et al. (2020)
Multiple Biotopes (8)	• Decrease in population biomass in fish (6) • Change in community composition in fish (1) • Multiple changes in ecosystem structure and function (1)	*Clupea harengus* (1), *Coregonus maraena* (1), *Lota lota* (1), *Osmerus eperlanus* (1), *Platichthys* sp. (1), *Salmo trutta* (1), Fish community (1), Littoral ecosystem (1)	Climate change has diverse impacts on shallow coastal waters. For instance, heatwaves induce effects resembling eutrophication, such as filamentous algae growth at the expense of bladderwrack; decrease in salinity weakens the success of marine species like bladderwrack, eelgrass, and blue mussels; changes in habitats favor macroalgae-dependent invertebrates and fish on hard substrates; climate change promotes the establishment of invasive species, which can affect the dynamics of the Baltic Sea food web (Viitasalo and Bonsdorff 2022).	Peltonen and Weigel (2022); Viitasalo and Bonsdorff (2022)

### Biodiversity loss is widespread across geographical areas, biotopes, and organism groups

**Fig. 3:**
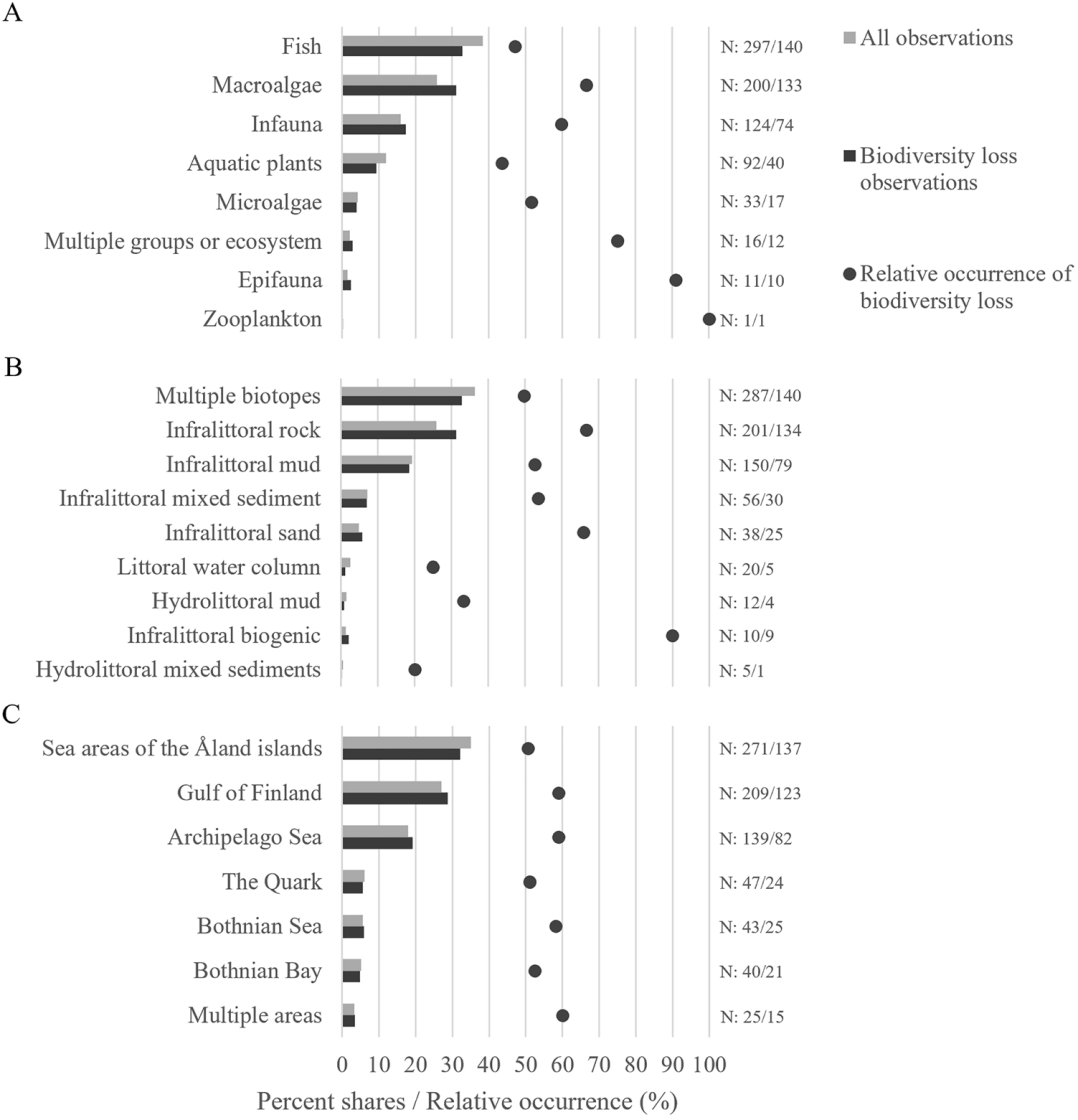
The percent shares of research observations relevant to biodiversity change categorized by organism groups (**A**), EUNIS level 3 classified Baltic Sea littoral biotopes (**B**), and Finnish marine areas (**C**). The percent shares for all included data (i.e., data that showed negative, positive or no change; n = 774) is shown in a lighter shade and the percent shares for biodiversity loss observations (i.e., negative changes; n = 427) in a darker shade. The relative occurrence of biodiversity loss indicated as black filled circles is presented as percent share of the biodiversity loss observations out of all included data in each of the categories. Number of observations in each category is indicated on the right side of the graphs (“N: all observations/biodiversity loss observations”)

Biodiversity loss was present in all marine areas, with most research observations (presented as the category-wise percent shares across categorization) found in the sea areas of the Åland Islands (32%), the Gulf of Finland (29%), and the Archipelago Sea (19%), together accounting for 80% of all biodiversity loss observations (Figs. 1 and 3C). The relative occurrence of biodiversity loss (presented as a percent share of biodiversity loss observations in a category relative to all included observations in that category) was highest in the Archipelago Sea and the Gulf of Finland (59%) and lowest around Åland Islands and in Gulf of Bothnia (51%; Fig. 3C).

Over half (64%) of the research observations could be associated to a specific littoral biotope (EU EUNIS biotopes).^2^ Of these the predominant biotope among the research observations indicating biodiversity loss was Infralittoral Rock (47%, Fig. 3B). No observations overall were associated with Coarse Sediments or Hydrolittoral Sand or Rock.^3^ The relative occurrence of biodiversity loss was highest for Infralittoral Biogenic Habitats (e.g., mussel beds; 90%) and lowest for Hydrolittoral Mixed Sediments (20%).

The literature indicating biodiversity loss concerned nearly 130 species or taxa (Table S5). Most research observations indicating biodiversity loss regarded fish (33%) and macroalgae (31%). Overall, there was only one research observation on zooplankton and no data on microbes. Among the organism groups with multiple observations the relative occurrence of biodiversity loss was highest in epifauna (91%) and lowest in aquatic vascular plants and charophytes (44%, Fig. 3C).

Various sources of bias inherently coupled to published research (publication bias, study interest and trends, location of research infrastructure, funding, etc.) restrict the interpretation of these findings. The extent of biodiversity loss or the importance of different expression forms cannot be fully addressed, likely underestimating the true scope of biodiversity loss in nature. The need for reducing research and publication bias, including improved reporting of both positive and negative or null effects should be emphasized (Wood 2020). Despite all inherent bias, the approach of this paper to synthesize available evidence from published research, summarizes for the first time the various ways biodiversity loss is expressed in the study area.

## Drivers of biodiversity loss

**Fig. 4:**
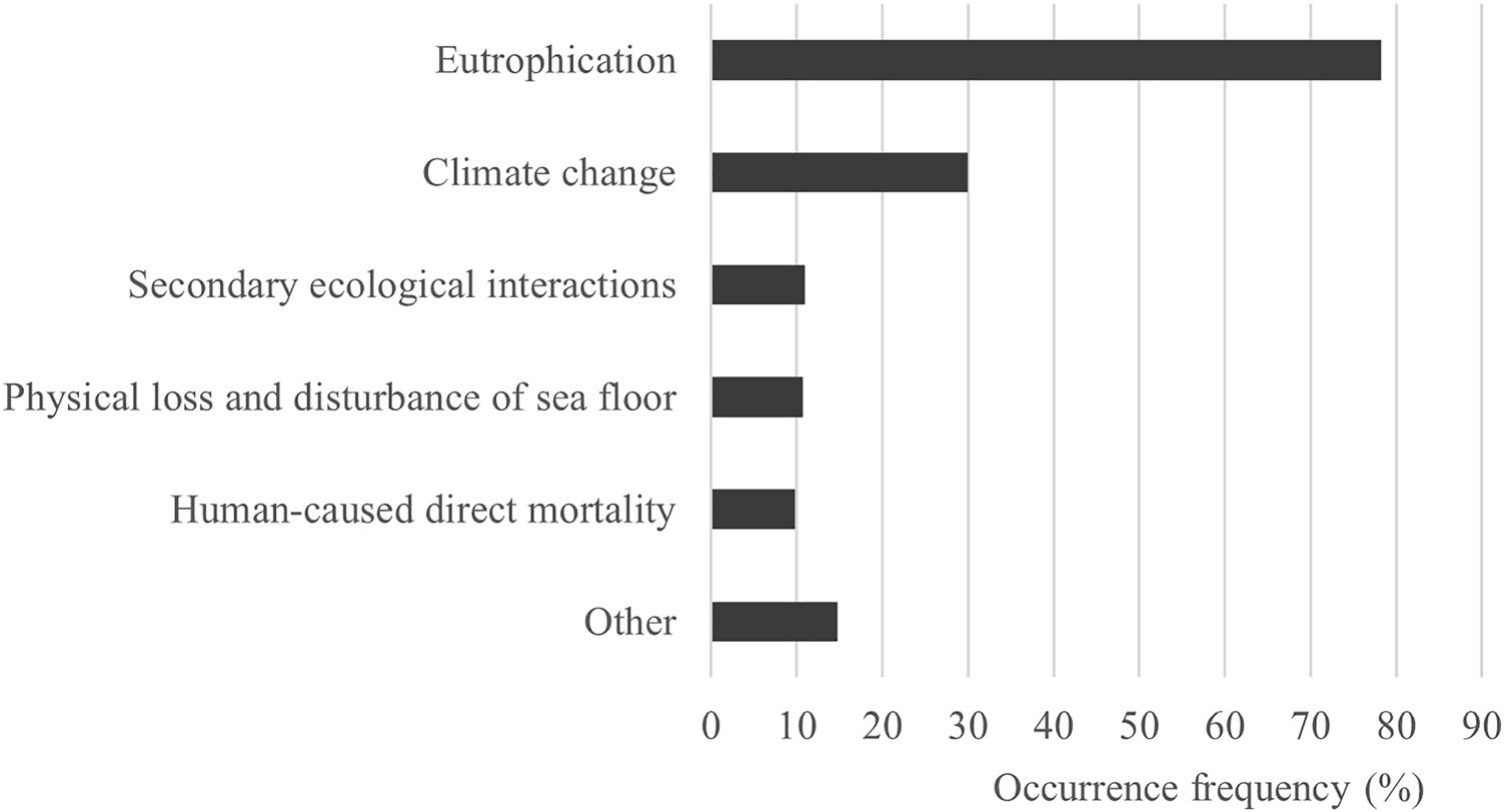
The occurrence frequency (%) of the five most common drivers of biodiversity loss in the biodiversity loss evidence dataset (n = 427). Each research observation indicating biodiversity loss may be associated with one or multiple loss drivers that all were counted separately, attaining a possible occurrence frequency between 0–100% for each individual driver. The “Other” category includes the following eight drivers with less than 7% occurrence frequencies: variation in key hydrological conditions, reduction of eutrophication, loss or deterioration of habitats, invasive species, acidification of water, artificial warming of water, species stocking of fish, and harmful substances

Thirteen drivers of biodiversity loss were identified from the literature. Eutrophication was the most common driver (Fig. 4), associated with nearly 80% of the biodiversity loss observations and with thirty expression forms. For the most common expression form, local disappearance of species, four underlying causes emerged: eutrophication, climate change, physical disturbance of the seabed, and ecological interactions. In 43% of the occasions more than one driver was associated with the biodiversity loss observation, suggesting that synergistic effects take place in biodiversity loss. For instance, most (88%) of the climate change related biodiversity loss observations were also linked to some other loss driver, eutrophication being the most common one (74%). However, these cumulative impacts and their effects were rarely investigated explicitly in the literature available.

The effects of eutrophication were common (50% prevalence in biodiversity loss observations) throughout the coast, except in Bothnian Bay, and visible in all organism groups, except for epifauna (Figs. S6–S8). Epifaunal communities have previously shown changes in biodiversity metrics along eutrophication gradients (e.g., Rinne et al. 2022), although these have not been studied temporally. Eutrophication was the main loss driver in 95–100% of the biodiversity loss records of microalgae, macroalgae, and aquatic plants and charophytes, and its prevalence in the literature was highest (> 80%) in the southern coastal areas (Figs. S6–S8). Eutrophication leads to changes in water and habitat quality and in trophic structure, disrupting biotic communities and ecosystems (e.g., Bonsdorff et al. 1997; Korpinen and Bonsdorff 2014). Eutrophication effects were manifested in multiple ways (Table 2), but most frequently as changed nutrient availability, reduced light penetration, and increased sedimentation affecting plants and algae (e.g., Eveleens Maarse et al. 2020). Eutrophication also caused habitat degradation for benthic invertebrates through organic enrichment and hypoxia (Weigel et al. 2015), and for fish, e.g., through increased filamentous algae (Jokinen et al. 2016).

Eutrophication is primarily caused by land-based nutrient (N and P) loading, and 50–75% of the total nutrient load to the coastal waters of Finland is directly caused by human activities (Fleming et al. 2023). Nutrient load levels are too high in all Finnish marine areas, which poses a significant challenge to reach good environmental status regarding eutrophication according to the EU MSFD (SYKE 2024). Additionally, the sustained internal loading of nutrients released from hypoxic sediments intensifies the situation (Puttonen et al. 2014). Given the magnitude of the eutrophication problem and the number of studies directed toward understanding its impacts, it is not surprising that it emerges as the main driver of biodiversity loss.

Climate change was the second most common (30% prevalence among records) driver of biodiversity loss (Fig. 4) and was associated with 18 expression forms, the most common ones being biomass decline and local disappearance of species. The prevalence of climate change as a loss driver increased radically (from 6 to 39%) from before to after the millennium shift, indicating increased climate change effects or research interest, or both (see Caro et al. 2022). The role of climate change was emphasized (> 50% prevalence) in the northern marine areas, especially in the Bothnian Sea and the Quark. In the literature, climate change was mostly presented as a potential driver of change and only one review article specifically addressed the various effects of climate change in marine coastal areas (Viitasalo and Bonsdorff 2022). Climate change has affected biodiversity mostly through increasing temperature and decreasing salinity, and  was identified as a driver of biodiversity loss in epifauna (Westerbom et al. 2019), soft bottom infauna (Weigel et al. 2015), and coastal fish (e.g., Peltonen and Weigel 2022; Table 2).

Physical disturbance of the seabed was associated with only 11% of the biodiversity loss observations (Fig. 4) and with 11 expression forms, the most common being decrease in species occurrence and local disappearance. Activities causing physical disturbance on the seabed include dredging and the deposition of dredged material, as well as coastal construction, port operations, and maritime traffic. Notably, it is estimated that there are at least about 36 000 small-scale (ø < 100 m) dredging areas, often linked to nearshore summer houses, covering roughly as much as five percent of the shallow soft bottoms of the Finnish coast (Virtanen et al. 2023). Fishing mortality was associated with approximately 10% of all biodiversity loss records (Fig. 4) and exhibited 12 different expression forms of biodiversity loss. The most common fishing-related expression forms were changes in age distribution and maturity and decrease in population biomass. Fishing causes direct mortality and selective fishing methods alter size and age distribution and may induce genetic changes toward slower growth and earlier maturation, leading to changes in population traits and productivity. Such effects have been observed for instance in migratory whitefish (*Coregonus lavaretus*) in the Bothnian Bay (Veneranta et al. 2021; Table 2).

Regarding harmful and hazardous substances, only one temporal research observation concerning deformities in herring (*Clupea harengus membras*; Rajasilta et al. 2016), was found in the literature. Marine litter or noise did not appear at all as causes of biodiversity loss, and there were only a few studies indicating direct effects of invasive species on biodiversity, mostly concerning benthic invertebrates (e.g., Packalén et al. 2008). Secondary changes through ecological interactions were the third most common cause of biodiversity loss (Fig. 4).

## Knowledge gaps regarding coastal biodiversity loss

Our literature study highlighted that there is a paucity of studies explicitly designed for detecting biodiversity loss. Our review found knowledge gaps across geographical areas, littoral biotopes, and organism groups along the Finnish coast, regarding several important aspects of detecting and understanding biodiversity loss.

***Research bias across geographical areas, biotopes, and organism groups.*** There is less research on biodiversity change in Gulf of Bothnia compared to southern coastal areas of Finland (Gulf of Finland, Åland Islands, Archipelago Sea). From the shallowest hydrolittoral zone or, e.g., regarding zooplankton or microbes, temporal research is not comprehensive or is missing altogether. Research observations on epifauna over time mostly concern the key species blue mussel (*Mytilus edulis*) and are scarce and limited to the Gulf of Finland. There is little or no temporal information on aquatic vascular plants and charophytes from four out of the six Finnish sea areas and only one study addressed temporal changes in eelgrass (*Zostera marina*) meadows (Boström et al. 2002), a key biotope supporting high biodiversity and nationally assessed as vulnerable (Kontula and Raunio 2018).

***Adaption potential and genetics.*** Particularly lacking is information on changes at the genetic level. Environmental pressures can drive very rapid evolutionary changes in organisms (Momigliano et al. 2017). Understanding genetic variation, adaptation potential and plasticity—and how they change—in key species, especially in species poor systems such as the Finnish coast, would be particularly important (Yu et al. 2020). Nature conservation and management practices that take genotypic diversity and evolutionary change into account will be more effective than previous practices (Lankau et al. 2011).

***Evidence-based links to and interactions among stress drivers.*** There seems to be a common issue in linking biodiversity loss to drivers in marine biodiversity research. In the assessed research literature, a relatively small fraction (8%) of the loss driver-biodiversity linkages was actually evidence-based and analytically tested. Thus, in the majority of the literature, most of the drivers are factors identified as potential drivers simply because they are common problems in the coastal environment. One way to overcome this issue, is to better integrate experimental stress ecology into studies of species, populations and food web dynamics (Villnäs et al. 2013; Korpinen et al. 2022). There is also a lack of information on the cumulative impacts of climate change, eutrophication and local pressures on the resilience of coastal ecosystems. Understanding these complex interactions is crucial for predicting and mitigating the long-term ecological consequences of multiple pressures (Reckermann et al. 2022; Viitasalo and Bonsdorff 2022). Moreover, the impact of the increasing prevalence of marine heatwaves are virtually unknown (Goebeler et al. 2022) and considering the high use of the coastal areas in recreation (i.e., summer cottages, boating) more studies are also needed on the cumulative biodiversity effects of local habitat disturbances (Virtanen et al. 2023).

***Ecosystem structure and functioning.*** Particularly lacking is information on ecosystem-level changes in shallow coastal areas. Cascading effects in food webs and ecosystem regime shifts have been studied and observed in the open Baltic Sea, but less so in coastal areas (Yletyinen et al. 2016; Tomczak et al. 2022). Our understanding of degrading ecosystem functioning is still lagging, but experimental in situ testing of stressors such as hypoxia (e.g., Villnäs et al. 2013) or marine heatwaves (Göbeler et al. 2025) suggest profound changes to, e.g., nutrient cycling. More attention should also be paid to less studied parts of ecosystems, e.g., microbial food webs and sea ice communities (Viitasalo and Bonsdorff 2022). This would enable a more comprehensive understanding of the population to ecosystem-level response to cumulative impacts of multiple pressures (Kortsch et al. 2021).

***Monitoring and inventory programs.*** There are also gaps in national biodiversity status assessments. Despite the large amount of data collected in the Finnish inventory program for underwater diversity (Forsblom et al. 2024), the threat status for a third of the underwater biotopes cannot be assessed due to data limitations (Kontula and Raunio 2018). Also, more than half of the inventories are focused on protected areas, highlighting the need for additional information from non-protected areas (Virtanen et al. 2022). There are also geographical and organism group specific gaps in the national marine monitoring programs (Rantajärvi et al. 2020). For example, coastal fish monitoring by gill net surveys is lacking from the entire Finnish part of the Gulf of Bothnia.

## Future perspectives and points of action

With advancing climate change amplifying the impacts of eutrophication—the main human-induced pressure in the Baltic Sea—biodiversity loss is not expected to slow down (Reckermann et al. 2022; Viitasalo and Bonsdorff 2022). In fact, these stress drivers are interlinked and often intensify each other (IPBES 2024), which emphasizes the need for better understanding of cumulative impacts from multiple stressors and more ambitious measures to combat the corresponding biodiversity loss. Having steep environmental gradients and a history of extensive disturbances and ecosystem degradation coupled with the early adoption of cross-border environmental management, it has been argued that the Baltic Sea can function as a “time machine” exceptionally well suited to investigate the effects of and potential remedies for future coastal ecosystem disruptions (Reusch et al. 2018)—making this study a case in point.

In the northern Baltic Sea, surface water salinity has decreased annually up to 0.02 since the 1960s (Kankaanpää et al. 2023). On the coast of western Gulf of Finland, seawater has warmed by 1.3–1.8 °C over the past century (1927–2020), and marine heatwaves have become more common and intense over time (Goebeler et al. 2022). Simultaneously, eutrophication status has not improved (SYKE 2024) and local stressors, such as habitat disturbances, are expected to increase (Virtanen et al. 2023). If species do not manage to adapt rapidly enough to new environmental conditions, it can have detrimental impacts on abundances and distribution (Vuorinen et al. 2015)—as predicted for instance for bladder wrack (*Fucus vesiculosus*), a key species in the Baltic Sea (Jonsson et al. 2018)—with serious effects on ecosystem structure and functioning (Yletyinen et al. 2016).

The monetary value of only the recreational benefits provided by the sea in Finland alone is over 2.5 B€ annually and improving the sea to a good environmental status would increase this value by over 700 M€ (HELCOM 2023c). Marine ecosystem services are in jeopardy if the elements of biodiversity producing these services are degraded or lost (Heckwolf et al. 2021; Jernberg et al. 2024). However, in a study by Jernberg et al. (2024) 15–51% of all potential linkages between natural capital stocks and ecosystem services could not be assessed because of data limitations, restricting the capacity of management and decision-making. This emphasizes the need for understanding marine coastal biodiversity loss and how it affects ecosystem services, in which we have significant economic and non-economic interests.

International and national biodiversity targets for increased protection and restoration of the Baltic Sea are in place (e.g., HELCOM 2021). However, the present actions are not enough. For instance, the measures proposed in the Finnish national marine action program are already initially self-assessed as insufficient, particularly regarding eutrophication, seabed integrity, biodiversity, and commercial fish stocks (Laamanen et al. 2021). Moreover, state-funded subsidies that harm the environment are maintained and regulation and current practices of environmental permitting have deficiencies, undermining efforts to halt biodiversity loss (HELCOM 2021, 2023a). Despite improvement in the agricultural sector, production intensification in farming is still subsidized through the EU Common Agricultural Policy causing continued nutrient pollution to the Baltic Sea (Heyl 2023). Also, for instance, the overall cumulative impacts of small-scale dredging activities in shallow coastal areas, currently exempt from environmental permitting obligations in Finland, should be better considered (Pappila and Puharinen 2022).

Halting biodiversity loss will require a society-wide transformation to put nature at the heart of all aspects of society and decision-making. This must be reflected in policymaking through binding commitments. Implementation of measures to halt coastal biodiversity loss should be accelerated and funding for it increased. Education and information transfer, i.e., transdisciplinary communication at all levels, to direct consumption behavior are key to enable society’s sustainability transition. Thus, knowledge of the status and role, both ecological but also economic, of marine coastal biodiversity among citizens and stakeholders should be considerably strengthened.

To advance our understanding of coastal biodiversity in the Baltic Sea, mapping biodiversity is only a first step. As identified in our results, biodiversity loss is far more complex than previously thought. Thus, monitoring should be developed, diversified and secured via long-term funding. In particular, monitoring schemes need to be developed to specifically address biodiversity loss, match the rate of change, and encompass all coastal areas. Monitoring and research also need to go beyond the most well-known habitats and species. Both the geographical research bias and the geographical features need to be better considered, as most species and habitats in the Baltic Sea occur along steep environmental gradients (e.g., salinity, nutrients and temperature) that may change due to global warming. Also, extensive monitoring to evaluate the impact of actions for reversing biodiversity loss is a prerequisite for successful adaptive management.

Reversing the negative coastal biodiversity trend synthesized in this study, encompasses not only reduction of pressures, but also significant protection measures and successful restoration efforts (EC 2020; HELCOM 2021). When developing marine species and habitat protection, typically threatened species and biotopes are prioritized at the expense of more common, but biodiversity-wise important ones (Virtanen and Moilanen 2023). Thus, a more holistic approach to developing the network of marine protected areas is needed (cf. Virtanen et al. 2018), where the habitat-forming and functionally important species are better considered (see ‘knowledge gaps’ above). Further, the demand for marine restorations is predicted to increase (Fraschetti et al. 2021) following the EU restoration law (EU 2024/1991), but efforts so far are still mostly short-term and small-scale (e.g., Gagnon et al. 2021). This calls for development and up-scaling of restoration practices. Importantly, however, the conditions for protection or restoration are not favorable if pressures have not been reduced. While reducing human-induced pressures is challenging, it is the only sustainable long-term solution to improve the state of the Baltic Sea, achievable through both international regulation and local measures (HELCOM ACTION 2021). According to the latest HELCOM assessment on the state of the Baltic Sea, measures to reduce pressures do work when implemented (HELCOM 2023a). 

As implicated by numerous previous works, and quantified in this study, eutrophication is among the most severe human-induced pressures and a major driver of biodiversity loss in coastal waters (Dai et al. 2023). Nutrient input from both agriculture and point sources (e.g., aquaculture) should be reduced by all means possible. As processes on land in the run-off area strongly impact receiving sea areas, reduction of diffuse loading via improved nutrient cycling, reforestation, and soil improvements could be achieved through tightening of legal instruments such as the Forest Act, the Water Act and EU’s Common Agricultural Policy (Boström et al. 2024). Minimizing eutrophication and local pressures is imperative for building resilience to global stressors such as climate change (IPBES 2024).

Finally, a solid knowledge base is central for comprehending the negative biodiversity trend and allows better targeting of limited resources to effective actions for agreed biodiversity goals (EC 2020). Importantly, however, despite knowledge limitations, enough information often already exists to confidently know the main human pressures and to state that biodiversity loss is widespread and the condition of coastal waters is poor, as in the case of the Finnish coast (HELCOM 2023a; SYKE 2024). This is likely true for most of the Baltic Sea and relevant for other regions as well, which warrants immediate action to save and safeguard the valuable coastal ecosystems.

## Supplementary Information

Below is the link to the electronic supplementary material.

Supplementary file1 (PDF 1617 KB)

## Footnotes

1 Certain cases regarding, e.g., population structure (age or length), species distribution, or complex community or ecosystem-level changes were difficult or impossible on linguistic (or on any other grounds) to clearly define as negative or positive but were nevertheless considered as forms of biodiversity loss in the context of this paper.

2 https://eunis.eea.europa.eu/habitats.jsp.

3 Although Hydrolittoral Rock did not directly occur as a biotope in the assessed literature, it is likely partly considered in studies assigned to Infralittoral Rock (e.g., concerning macroalgae).
