# Supplementary material for: Marine biodiversity loss in Finnish coastal waters: Evidence and implications for management
Source: Ambio. 2025 May 16;54(11):1786–808. doi: 10.1007/s13280-025-02185-x (PMC12480322; doi:10.1007/s13280-025-02185-x)
Supplement: Supplementary file 1 — Supplementary file1 (PDF 1617 KB) [file 13280_2025_2185_MOESM1_ESM.pdf]

Supplementary Information

Marine biodiversity loss in Finnish coastal waters: evidence and implications for management

Henri Sumelius, Samuli Korpinen, Alf Norkko, Sonja Salovius-Laurén, Markku Viitasalo, Christoffer Boström

Corresponding author: Henri Sumelius, Åbo Akademi University

Institutional mailing address: Åbo Akademi University, Environmental and Marine Biology,

Aurum, Henriksgatan 2, 20500 Åbo, Finland; email: henri.sumelius@abo.fi

Ambio, Electronic Supplementary Material

This supplementary information has not been peer reviewed.

Contents

1 Systematic literature search 2
1.1 Literature search Level 1 2
1.2 Literature search Level 2 5
1.3 Literature search Level 3 8
1.4 Literature search results 16
2 Screening of search records and data 20
3 Processing and analysis of research data 21
4 Outcomes from the literature search and supplemental results of the assessment 22

1 Systematic literature search

A systematic literature search of published peer-reviewed scientific literature was performed (10/2022). The search targeted articles that would potentially contain observations on changes in biodiversity and expressions of nature loss in shallow littoral areas along the Finnish coast and archipelago of the Baltic Sea. No temporal limitations related to publication year were defined for the search. The search focused on temporal trend studies or time point comparisons, with data structures that could indicate changes in biodiversity metrics, indicators, or any other elements of biodiversity.

The primarily study question for the literature search was: *How is marine nature/biodiversity loss expressed in shallow coastal habitats in Finland?* Based on the study question, first a search strategy and structural set-up for the literature search were developed. Then, the study question was partitioned into its main elements, followed by a listing of relevant search words. The literature search strategy was developed so that a query structure of three basic Levels was constructed. The different Levels correspond to the amount of inclusive detail used in identifying search words and building search strings for sub-queries, aiming for increased relevant coverage with each sub-sequent query Level, with the best coverage and potential for highest number of found literature records from the full query search at Level 3.

The literature search was conducted in English, as the selected peer-reviewed scientific articles are almost exclusively written in English, but no hits were excluded based on language. Search queries and sub-queries with complete search strings were constructed and consecutive runs of the search queries were made in **Web of Science** (All Databases including Web of Science Core Collection, BIOSIS Citation Index, Current Contents Connect, Data Citation Index, Derwent Innovations Index, MEDLINE®, SciELO Citation Index, and Zoological Record) and **Scopus** databases according to search strategy and set-up.

1.1 Literature search Level 1

Level 1 of the literature search dissects the study question into three main elements to be answered/targeted by the literature search: Area and Environment, Research subject, and Phenomenon. The query at Level 1 can be seen as the core apex of the search, whereas the subsequent Levels aim at widening the search in a purposeful way to cover more potentially relevant papers.

The Level 1 query is composed of three sub-queries corresponding to the three main elements of the search as Q 1.1 – Area and Environment, Q 1.2 – Research subject, and Q 1.3 – Phenomenon. The Web of Science search string of Level 1 query is thus:

Query 1: (TS=(Q 1.1 AND Q 1.2 AND Q 1.3))

where *TS* stands for search field code "*Topic*", searching for topic terms in the Title, Abstract, and keywords fields within a record. The Scopus search string of Level 1 query is:

Query 1: TITLE-ABS-KEY(Q 1.1 AND Q 1.2 AND Q 1.3)

where *TITLE-ABS-KEY* stands for search field code "*Doc Title, Abstract, Keyword*", searching for topic terms in the Title, Abstract, and keywords fields within a record.

Q 1.1 – Area and Environment

Sub-query Q 1.1 aims to cover and target the area and environment of the literature records matching with the study question, "*marine, shallow coastal areas in Finland*". The area and environment consist of the following terms defined:

Area:

- Finland ***[Finland OR Finnish OR Åland* OR Aland*]***
  AND
- marine areas ***[marine OR sea OR brackish]***
  OR
- Finnish sea areas ***["Bothnian Bay" OR "Bay of Bothnia" OR "Gulf of Bothnia" OR Quark OR Kvarken OR "Bothnian Sea" OR "Archipelago Sea" OR "Gulf of Finland"]*** Address ***[Finland]***
- Baltic Sea ***["Baltic Sea"]*** Address ***[Finland]***

Environment:

- shallow coastal areas ***[shallow OR littoral OR infralittoral OR *shore* OR flad* OR glo* OR lagune* OR coast* OR photic OR euphotic OR "sunlight zone*" OR "light zone*" OR bay* OR estuar*]***

The Web of Science search string of Level 1 sub-query Q 1.1 is thus:

Q 1.1: ((TS=(Finland OR Finnish OR Åland* OR Aland*) AND TS=(marine OR sea OR brackish)) OR (TS=("Baltic Sea" OR "Bothnian Bay" OR "Bay of Bothnia" OR "Gulf of Bothnia" OR Quark OR Kvarken OR "Bothnian Sea" OR "Archipelago Sea" OR "Gulf of Finland") AND AD=(Finland))) AND TS=(shallow OR littoral OR infralittoral OR *shore* OR flad* OR glo* OR lagune* OR coast* OR photic OR euphotic OR "sunlight zone*" OR "light zone*" OR bay* OR estuar*)

The Scopus search string of Level 1 sub-query *Q 1.1* is thus:

Q 1.1: ((TITLE-ABS-KEY (finland OR finnish OR åland* OR aland*) AND TITLE-ABS-KEY (marine OR sea OR brackish)) OR (TITLE-ABS-KEY ("Baltic Sea" OR "Bothnian Bay" OR "Bay of Bothnia" OR "Gulf of Bothnia" OR quark OR kvarken OR "Bothnian Sea" OR "Archipelago Sea" OR "Gulf of Finland") AND AFFILCOUNTRY (Finland))) AND (TITLE-ABS-KEY (shallow OR littoral OR infralittoral OR *shore* OR flad* OR glo* OR lagune* OR coast* OR photic OR euphotic OR "sunlight zone*" OR "light zone*" OR bay* OR estuar*))

Q 1.2 – Research subject

Sub-query Q 1.2 aims to cover and target the research subject of the literature records matching with the study question, "*nature/biodiversity*". This is here composed of different common components of nature as including the different main aspects of biodiversity, namely genetic diversity, species diversity, phylogenetic diversity, habitat and ecosystem Level diversity, and functional diversity. The research subject consists of the following terms defined:

Research subject:

- components of nature ***[nature OR ecolog* OR biolog* OR gene* OR geno* OR allel* OR phenotyp* OR phylogenetic* OR taxon* OR *species OR population* OR ecotype* OR stock* OR assemblage* OR communit* OR "food web" OR foodweb OR food-web OR trophic OR biotope* OR habitat* OR biome* OR ecosystem* OR trait* OR "functional group*" OR "functional role*"]***

  OR

- biodiversity ***[biodiversit* OR *diversit* OR *richness* OR *evenness* OR *distinctness* OR *similarit* OR *disparit* OR *divergence* OR heterogeneit* OR FGR OR FAD OR BEF OR biodiversity-ecosystem OR diversity-ecosystem OR endanger* OR extinct* OR "diversity ind*" OR "Simpson index" OR Shannon-Wiener OR Margalef*]***

The Web of Science search string of Level 1 sub-query Q 1.2 is thus:

> Q 1.2: ***TS=(nature OR ecolog* OR biolog* OR gene* OR geno* OR allel* OR phenotyp* OR phylogenetic* OR taxon* OR *species OR population* OR ecotype* OR stock* OR assemblage* OR communit* OR "food web" OR foodweb OR food-web OR trophic OR biotope* OR habitat* OR biome* OR ecosystem* OR trait* OR "functional group*" OR "functional role*" OR biodiversit* OR *diversit* OR *richness* OR *evenness* OR *distinctness* OR *similarit* OR *disparit* OR *divergence* OR heterogeneit* OR FGR OR FAD OR BEF OR biodiversity-ecosystem OR diversity-ecosystem OR endanger* OR extinct* OR "diversity ind*" OR "Simpson index" OR Shannon-Wiener OR Margalef*)***

The Scopus search string of Level 1 sub-query Q 1.2 is thus:

> ***Q 1.2: TITLE-ABS-KEY(nature OR ecolog* OR biolog* OR gene* OR geno* OR allel* OR phenotyp* OR phylogenetic* OR taxon* OR *species OR population* OR ecotype* OR stock* OR assemblage* OR communit* OR "food web" OR foodweb OR food-web OR trophic OR biotope* OR habitat* OR biome* OR ecosystem* OR trait* OR "functional group*" OR "functional role*" OR biodiversit* OR *diversit* OR *richness* OR *evenness* OR *distinctness* OR *similarit* OR *disparit* OR *divergence* OR heterogeneit* OR FGR OR FAD OR BEF OR biodiversity-ecosystem OR diversity-ecosystem OR endanger* OR extinct* OR "diversity ind*" OR "Simpson index" OR Shannon-Wiener OR Margalef*)***

Q 1.3 – Phenomenon

Sub-query Q 1.3 aims to cover and target the phenomenon assessed in the literature records matching with the study question, "*(nature) loss*"[1]. The phenomenon consists of the following terms defined:

Phenomenon:

---

[1] After the decision to also include evidence that potentially could have exhibited a negative change but that did not and instead show positive changes or no changes at all, a post-search testing of the effect of including the search-word "***increase****", was conducted. This test did not produce any additional eligible search hits compared to the original search.

- loss/degradation/change ***[loss* OR collaps* OR crash* OR degrad* OR declin* OR decreas* OR reduc* OR weakening* OR diminishing* OR worsening* OR diverg* OR develop* OR shift* OR alteration* OR temporal OR long-term OR "difference between timepoints" OR "difference between periods" OR trend* OR "past decade*" OR "over time" OR "pattern* in time" OR "over year*" OR "across year*" OR "over decade*" OR chang* OR variabilit* OR variation* OR homogeni* OR "more homogeneous" OR "increasingly homogeneous" OR status* OR state*]***

The Web of Science search string of Level 1 sub-query Q 1.3 is thus:

> ***Q 1.3: TS=(loss* OR collaps* OR crash* OR degrad* OR declin* OR decreas* OR reduc* OR weakening* OR diminishing* OR worsening* OR diverg* OR develop* OR shift* OR alteration* OR temporal OR long-term OR "difference between timepoints" OR "difference between periods" OR trend* OR "past decade*" OR "over time" OR "pattern* in time" OR "over year*" OR "across year*" OR "over decade*" OR chang* OR variabilit* OR variation* OR homogeni* OR "more homogeneous" OR "increasingly homogeneous" OR status* OR state*)***

The Scopus search string of Level 1 sub-query Q 1.3 is thus:

> ***Q 1.3: TITLE-ABS-KEY(loss* OR collaps* OR crash* OR degrad* OR declin* OR decreas* OR reduc* OR weakening* OR diminishing* OR worsening* OR diverg* OR develop* OR shift* OR alteration* OR temporal OR long-term OR "difference between timepoints" OR "difference between periods" OR trend* OR "past decade*" OR "over time" OR "pattern* in time" OR "over year*" OR "across year*" OR "over decade*" OR chang* OR variabilit* OR variation* OR homogeni* OR "more homogeneous" OR "increasingly homogeneous" OR status* OR state*)***

1.2 Literature search Level 2

Level 2 aims at widening the search in a purposeful way to cover more potentially relevant papers. The Level 2 Query builds upon Query 1 from the previous Level 1, by widening the concept of the research subject "*nature*", for which "*biodiversity*" was used as the quality element in focus, through adding Level 2 sub-queries to the Q 1.2 sub-query term of Query 1. As biodiversity is defined as "the variability among living organisms from all sources including terrestrial, marine, and other aquatic ecosystems and the ecological complexes of which they are a part. This includes variation in genetic, phenotypic, phylogenetic, and functional attributes, as well as changes in abundance and distribution over time and space within and among species, biological communities, and ecosystems" (IPBES 2019), the searched concept of biodiversity was elaborated to encompass more aspects of biodiversity than what just the core terminology of biodiversity and its sub-categorizations can as such manage to do.

The Level 2 Query is composed of two sub-queries all aiming at including more of the relevant aspects of nature and biodiversity to the search. These sub-queries are Q 2.1 – Additional aspects of biodiversity and Q 2.2 – Main organism groups/communities/assemblages. The Web of Science search string of Level 2 Query is thus:

> ***Query 2: TS=(Q 1.1 AND (Q 1.2 OR Q 2.1 OR Q 2.2) AND Q 1.3)***

The Scopus search string of Level 2 Query is:

> ***Query 2: TITLE-ABS-KEY(Q 1.1 AND (Q 1.2 OR Q 2.1 OR Q 2.2) AND Q 1.3)***

Q 2.1 – Additional aspects of biodiversity

Sub-query Q 2.1 aims to cover and target additional aspects of biodiversity. These consist of the following terms defined:

Additional aspects of biodiversity:

- biological entities/measurements/functions ***[phenology* OR abundance* OR biomass* OR densit* OR distribution* OR frequenc* OR occurrence* OR composition* OR turnover* OR "biotic integrit*" OR "biotic intactness*" OR "biotic resilience*" OR "functional integrit*" OR "functional intactness* OR "functional resilience" OR "primary producti*" OR "secondary producti*" OR "carbon sequestration" OR "nutrient recycling" OR decomposition]***
- population characteristics and life histories ***[lifehistor* OR life-histor* OR "life history*" OR longevit* OR "age distribution*" OR "age composition*" OR "age structure*" OR "mean age*" OR "average age* OR "size distribution*" OR "size composition*" OR "size structure*" OR "mean size*" OR "average size*" OR "length distribution*" OR "length composition*" OR "length structure*" OR "mean length*" OR "average length*" OR "length at age" OR length-at-age OR bodymass OR body-mass OR "body mass" OR "mean weight" OR "average weight" OR "weight at age" OR weight-at-age OR weight-at-length OR "weight at length" OR "body condition" OR "Fulton* condition" OR "Fulton index" OR maturity OR growth OR reproduct* OR spawning]***

The Web of Science search string of Level 2 sub-query Q 2.1 is thus:

> ***Q 2.1: TS=(phenology* OR abundance* OR biomass* OR densit* OR distribution* OR frequenc* OR occurrence* OR composition* OR turnover* OR "biotic integrit*" OR "biotic intactness*" OR "biotic resilience*" OR "functional integrit*" OR "functional intactness*" OR "functional resilience" OR "primary producti*" OR "secondary producti*" OR "carbon sequestration" OR "nutrient recycling" OR decomposition OR lifehistor* OR life-histor* OR "life history*" OR longevit* OR "age distribution*" OR "age composition*" OR "age structure*" OR "mean age*" OR "average age*" OR "size distribution*" OR "size composition*" OR "size structure*" OR "mean size*" OR "average size*" OR "length distribution*" OR "length composition*" OR "length structure*" OR "mean length*" OR "average length*" OR "length at age" OR length-at-age OR bodymass OR body-mass OR "body mass" OR "mean weight" OR "average weight" OR "weight at age" OR weight-at-age OR weight-at-length OR "weight at length" OR "body condition" OR "Fulton* condition" OR "Fulton index" OR maturity OR growth OR reproduct* OR spawning)***

The Scopus search string of Level 2 sub-query Q 2.1 is thus:

> ***Q 2.1: TITLE-ABS-KEY(phenology* OR abundance* OR biomass* OR densit* OR distribution* OR occurrence* OR composition* OR turnover* OR "biotic integrit*" OR "biotic intactness*" OR "biotic resilience*" OR "functional integrit*" OR "functional intactness*" OR "functional resilience" OR "primary producti*" OR "secondary producti*" OR "carbon sequestration" OR "nutrient recycling" OR decomposition OR lifehistor* OR life-histor* OR "life history*" OR longevit* OR "age distribution*" OR "age composition*" OR "age structure*" OR "mean age*" OR "average age*" OR "size distribution*" OR "size composition*" OR "size structure*" OR "mean size*" OR "average size*" OR "length distribution*" OR "length composition*" OR "length structure*" OR "mean length*" OR "average length*" OR "length at age" OR length-at-age OR bodymass OR body-mass OR "body mass" OR "mean weight" OR "average weight" OR "weight at age" OR weight-at-age OR weight-at-length OR "weight at length" OR "body condition" OR "Fulton* condition" OR "Fulton index" OR maturity OR growth OR reproduct* OR spawning)***

Q 2.2 – Main organism groups/communities/assemblages

Sub-query Q 2.2 aims to cover and target main organism groups, communities, and assemblages as aspects of biodiversity. Main organism groups/communities/assemblages consist of the following terms defined:

Main organism groups/communities/assemblages:

- bacteria ***[bacteria OR microbe* OR picoplankt* OR Spirulina OR Rivularia OR Calothrix]***

  OR

- aquatic vegetation, including water mosses ***[“aquatic vegetation*” OR “aquatic flora” OR macrophyte* OR “aquatic plant*” OR “water plant*” OR “sea grass*” OR seagrass* OR “reed belt*” OR “water moss*” OR “underwater meadow*”]***

  OR

- macroalgae, including charophytes ***[macroalgae OR “sea weed*” OR seaweed* OR charophyte* OR “brown algae” OR Phaeophyt* OR “red algae” OR Rhodophyt* OR “green algae” OR Chlorophyt* OR “filamentous algae”]***

  OR

- phytoplankton ***[phytoplankton OR microalgae OR "autotroph* *plankt*" OR "mixotroph* *plankt*" OR "photosynthe* *plankt*" OR "autotroph* microorganism*" OR "mixotroph* microorganism*" OR "photosynthe* microorganism*" OR cyanobacteria OR diatom* OR Chrysophyt* OR Cyanophyt* OR Cryptophyt* OR Dinophyt* OR Haptophyt* OR Euglenophyt* OR Chlorophyt* OR Zoomastigophor* OR “Incertae cedis” OR Bacillariophyt*]***

  OR

- zooplankton ***[*zooplankton OR "heterotroph* *plankt*" OR "heterotroph* microorganism*" OR “planktonic crustacean*” OR Radiolaria* OR Foraminifera* OR Amoeba OR Ciliate* OR Dinoflagellate*]***

  OR

- benthic invertebrates ***[“benthic invertebrate*” OR “benthic animal*” OR “benthic fauna*” OR *benth* OR infauna* OR “benthic macrofauna*” OR “benthic meiofauna*” OR “burrow* animal*” OR “burrow* fauna*” OR “burrow* macrofauna*” OR “burrow* meiofauna*” OR “burrow* invertebrate*”]***

  OR

- epibenthic, epiphytic and epizoic fauna ***[epibenthos OR “epibenthic fauna” OR “epibenthic animal*” OR epifauna OR epiphytes OR “epiphytic fauna” OR “epiphytic animal*” OR epiphyte* OR epizoic OR “associated fauna” OR “associated invertebrate fauna”]***

  OR

- periphyton [***periphyt****]

  OR

- fish [***fish****]

The Web of Science search string of Level 2 sub-query Q 2.2 is thus:

> ***Q 2.2: TS=(bacteria OR microbe* OR picoplankt* OR Spirulina OR Rivularia OR Calothrix OR “aquatic vegetation*” OR “aquatic flora” OR macrophyte* OR “aquatic plant*” OR “water plant*” OR “sea grass*” OR seagrass* OR “reed belt*” OR “water moss*” OR “underwater meadow*” OR macroalgae OR “sea weed*” OR seaweed* OR charophyte* OR “brown***

algae" OR Phaeophyt* OR "red algae" OR Rhodophyt* OR "green algae" OR Chlorophyt* OR "filamentous algae" OR phytoplankton OR microalgae OR "autotroph* *plankt*" OR "mixotroph* *plankt*" OR "photosynthe* *plankt*" OR "autotroph* microorganism*" OR "mixotroph* microorganism*" OR "photosynthe* microorganism*" OR cyanobacteria OR diatom* OR Chrysophyt* OR Cyanophyt* OR Cryptophyt* OR Dinophyt* OR Haptophyt* OR Euglenophyt* OR Chlorophyt* OR Zoomastigophor* OR "Incertae cedis" OR Bacillariophyt* OR *zooplankton OR "heterotroph* *plankt*" OR "heterotroph* microorganism*" OR "planktonic crustacean*" OR Radiolaria* OR Foraminifera* OR Amoeba OR Ciliate* OR Dinoflagellate* OR "benthic invertebrate*" OR "benthic animal*" OR "benthic fauna*" OR *benth* OR infauna* OR "benthic macrofauna*" OR "benthic meiofauna*" OR "burrow* animal*" OR "burrow* fauna*" OR "burrow* macrofauna*" OR "burrow* meiofauna*" OR "burrow* invertebrate*" OR epibenthos OR "epibenthic fauna" OR "epibenthic animal*" OR epifauna OR epiphytes OR "epiphytic fauna" OR "epiphytic animal*" OR epiphyte* OR epizoic OR "associated fauna" OR "associated invertebrate fauna" OR periphyt* OR fish*)

The Scopus search string of Level 2 sub-query Q 2.2 is thus:

Q 2.2: TITLE-ABS-KEY(bacteria OR microbe* OR picoplankt* OR Spirulina OR Rivularia OR Calothrix OR "aquatic vegetation*" OR "aquatic flora" OR macrophyte* OR "aquatic plant*" OR "water plant*" OR "sea grass*" OR seagrass* OR "reed belt*" OR "water moss*" OR "underwater meadow*" OR macroalgae OR "sea weed*" OR seaweed* OR charophyte* OR "brown algae" OR Phaeophyt* OR "red algae" OR Rhodophyt* OR "green algae" OR Chlorophyt* OR "filamentous algae" OR phytoplankton OR microalgae OR "autotroph* *plankt*" OR "mixotroph* *plankt*" OR "photosynthe* *plankt*" OR "autotroph* microorganism*" OR "mixotroph* microorganism*" OR "photosynthe* microorganism*" OR cyanobacteria OR diatom* OR Chrysophyt* OR Cyanophyt* OR Cryptophyt* OR Dinophyt* OR Haptophyt* OR Euglenophyt* OR Chlorophyt* OR Zoomastigophor* OR "Incertae cedis" OR Bacillariophyt* OR *zooplankton OR "heterotroph* *plankt*" OR "heterotroph* microorganism*" OR "planktonic crustacean*" OR Radiolaria* OR Foraminifera* OR Amoeba OR Ciliate* OR Dinoflagellate* OR "benthic invertebrate*" OR "benthic animal*" OR "benthic fauna*" OR *benth* OR infauna* OR "benthic macrofauna*" OR "benthic meiofauna*" OR "burrow* animal*" OR "burrow* fauna*" OR "burrow* macrofauna*" OR "burrow* meiofauna*" OR "burrow* invertebrate*" OR epibenthos OR "epibenthic fauna" OR "epibenthic animal*" OR epifauna OR epiphytes OR "epiphytic fauna" OR "epiphytic animal*" OR epiphyte* OR epizoic OR "associated fauna" OR "associated invertebrate fauna" OR periphyt* OR fish*)

1.3 Literature search Level 3

Level 3 aims at even further widening the search to cover more potentially relevant papers. The Level 3 Query builds upon Query 2 from the previous Level 2, by elaborating the concept of "*biodiversity*", through adding Level 3 sub-queries to the Q 1.2, Q 2.1, and Q 2.2 sub-query terms of Query 2. At this Level species and higher taxonomic common names and scientific nomenclature is added to the search.

The Level 3 Query is composed of two sub-queries all aiming at including more of the relevant aspects of nature and biodiversity to the search. These sub-queries are Q 3.1 – Organism and taxon common names and Q 3.2 – Scientific nomenclature of taxon. The Web of Science search string of Level 3 Query is thus:

Query 3: TS=(Q 1.1 AND (Q 1.2 OR Q 2.1 OR Q 2.2 OR Q 3.1 OR Q 3.2) AND Q 1.3)

The Scopus search string of Level 3 Query is thus:

Query 3: TITLE-ABS-KEY(Q 1.1 AND (Q 1.2 OR Q 2.1 OR Q 2.2 OR Q 3.1 OR Q 3.2) AND Q 1.3)

Q 3.1 – Organism and taxon common names

Sub-query Q 3.1 aims to cover and target organism and taxon common names as aspects of biodiversity. Organism and taxon common names consist of the following terms defined:

- Common names in English ***[barnacle* OR prawn* OR shrimp* OR crab* OR sturgeon* OR eel* OR garfish* OR flounder* OR flatfish OR turbot* OR herring* OR sprat* OR trout* OR seatrout* OR "sea trout*" OR whitefish* OR carp* OR bream* OR belica* OR dace* OR minnow* OR ide* OR sichel* OR roach* OR bleak* OR rudd* OR tench* OR loach* OR pike* OR cod* OR burbot* OR goby OR gobies OR smelt* OR sandeel* OR perch* OR ruffe* OR pike-perch* OR pikeperch* OR zander* OR "European bullhead*" OR lumpsucker* OR seasnail* OR sculpin* OR stickleback* OR blenny* OR gunnel* OR eelpout* OR vendace* OR salmon* OR grayling* OR pipefish* OR ragworm* OR leach* OR "trembling sea mat*" OR "moon jelly*" OR jellyfish* OR jelly-fish* OR snail* OR mudsnail* OR ramshorn* OR mussel* OR cockle* OR clam* OR polyp* OR "ribbon worm*" OR "water insect*" OR "aquatic insect*" OR "bristle worm*" OR mollusk* OR flatworm* OR "flat worm*" OR "wheel animal*" OR sponge* OR stoneworth* OR pondweed* OR bladderwrack OR bladder-wrack OR "bladder wrack" OR eelgrass* OR reed*]***

  OR

- Higher taxon anglified names ***[arthropod* OR mollusc* OR plathyhelminth* OR nematode* OR annelid* OR polychaete* OR cnidaria* OR bryozoan* OR rotifer* OR crustacean* OR bivalve* OR gastrop* OR hydrozoan* OR insect* OR malacostracan* OR amphipod* OR clupeid* OR cyprinid* OR decapod* OR isopod* OR mysid* OR percid* OR chironomid* OR gammarid* OR gobid* OR salmonid* OR copepod* OR calanoid* OR naiad* OR phanerogam* OR spermatophyt*]***

The Web of Science search string of Level 3 sub-query Q 3.1 is thus:

> ***Q 3.1: TS=(barnacle* OR prawn* OR shrimp* OR crab* OR sturgeon* OR eel* OR garfish* OR flounder* OR flatfish OR turbot* OR herring* OR sprat* OR trout* OR seatrout* OR "sea trout*" OR whitefish* OR carp* OR bream* OR belica* OR dace* OR minnow* OR ide* OR sichel* OR roach* OR bleak* OR rudd* OR tench* OR loach* OR pike* OR cod* OR burbot* OR goby OR gobies OR smelt* OR sandeel* OR perch* OR ruffe* OR pike-perch* OR pikeperch* OR zander* OR "European bullhead*" OR lumpsucker* OR seasnail* OR sculpin* OR stickleback* OR blenny* OR gunnel* OR eelpout* OR vendace* OR salmon* OR grayling* OR pipefish* OR ragworm* OR leach* OR "trembling sea mat*" OR "moon jelly*" OR jellyfish* OR jelly-fish* OR snail* OR mudsnail* OR ramshorn* OR mussel* OR cockle* OR clam* OR polyp* OR "ribbon worm*" OR "water insect*" OR "aquatic insect*" OR "bristle worm*" OR mollusk* OR flatworm* OR "flat worm*" OR "wheel animal*" OR sponge* OR stoneworth* OR pondweed* OR bladderwrack OR bladder-wrack OR "bladder wrack" OR eelgrass* OR reed* OR arthropod* OR mollusc* OR plathyhelminth* OR nematode* OR annelid* OR polychaete* OR cnidaria* OR bryozoan* OR rotifer* OR crustacean* OR bivalve* OR gastrop* OR hydrozoan* OR insect* OR malacostracan* OR amphipod* OR clupeid* OR cyprinid* OR decapod* OR isopod* OR mysid* OR percid* OR chironomid* OR gammarid* OR gobid* OR salmonid* OR copepod* OR calanoid* OR naiad* OR phanerogam* OR spermatophyt*)***

The Scopus search string of Level 3 sub-query Q 3.1 is thus:

> ***Q 3.1: TITLE-ABS-KEY(barnacle* OR prawn* OR shrimp* OR crab* OR sturgeon* OR eel* OR garfish* OR flounder* OR flatfish OR turbot* OR herring* OR sprat* OR trout* OR seatrout* OR "sea trout*" OR whitefish* OR carp* OR bream* OR belica* OR dace* OR minnow* OR***

ide* OR sichel* OR roach* OR bleak* OR rudd* OR tench* OR loach* OR pike* OR cod* OR burbot* OR goby OR gobies OR smelt* OR sandeel* OR perch* OR ruffe* OR pike-perch* OR pikeperch* OR zander* OR "European bullhead*" OR lumpsucker* OR seasnail* OR sculpin* OR stickleback* OR blenny* OR gunnel* OR eelpout* OR vendace* OR salmon* OR grayling* OR pipefish* OR ragworm* OR leach* OR "trembling sea mat*" OR "moon jelly*" OR jellyfish* OR jelly-fish* OR snail* OR mudsnail* OR ramshorn* OR mussel* OR cockle* OR clam* OR polyp* OR "ribbon worm*" OR "water insect*" OR "aquatic insect*" OR "bristle worm*" OR mollusk* OR flatworm* OR "flat worm*" OR "wheel animal*" OR sponge* OR stoneworth* OR pondweed* OR bladderwrack OR bladder-wrack OR "bladder wrack" OR eelgrass* OR reed* OR arthropod* OR mollusc* OR plathyhelminth* OR nematode* OR annelid* OR polychaete* OR cnidaria* OR bryozoan* OR rotifer* OR crustacean* OR bivalve* OR gastrop* OR hydrozoan* OR insect* OR malacostracan* OR amphipod* OR clupeid* OR cyprinid* OR decapod* OR isopod* OR mysid* OR percid* OR chironomid* OR gammarid* OR gobid* OR salmonid* OR copepod* OR calanoid* OR naiad* OR phanerogam* OR spermatophyt*)

Q 3.2 – Scientific nomenclature of taxon

Sub-query Q 3.2 aims to cover and target organisms and taxon common names as aspects of biodiversity. Scientific nomenclature of taxon consists of the following terms defined:

- higher taxon_Class ***[Actinopteri OR Bangiophyceae OR Bivalvia OR Bryopsida OR Charophyceae OR Chlorophyceae OR Clitellata OR Demospongiae OR Florideophyceae OR Gastropoda OR Gymnolaemata OR Hexanauplia OR Hoplonemertea OR Hydrozoa OR Insecta OR Magnoliopsida OR Malacostraca OR Petromyzonti OR Phaeophyceae OR Phylactolaemata OR Polychaeta OR Scyphozoa OR Trebouxiophyceae OR Ulvophyceae OR Xanthophyceae]***

  OR

- higher taxon_Order ***[Acipenseriformes OR Acrochaetiales OR Ahnfeltiales OR Alismatales OR Amphipoda OR Anguilliformes OR Anthoathecata OR Architaenioglossa OR Bangiales OR Beloniformes OR Carangiformes OR Cardiida OR Ceramiales OR Chaetophorales OR Charales OR Cheilostomata OR Cladophorales OR Clupeiformes OR Coleoptera OR Ctenostomatida OR Cypriniformes OR Decapoda OR Dicranales OR Diptera OR Ectocarpales OR Esociformes OR Fucales OR Gadiformes OR Gigartinales OR Gobiiformes OR Hemiptera OR Hildenbrandiales OR Hypnales OR Isopoda OR Lamiales OR Laminariales OR Leptothecata OR Limnomedusae OR Littorinimorpha OR Myoida OR Mysida OR Mytilida OR Nudibranchia OR Odonata OR Osmeriformes OR Perciformes OR Petromyzontiformes OR Phyllodocida OR Poales OR Prasiolales OR Ranunculales OR Rhynchobdellida OR Sabellida OR Salmoniformes OR Saxifragales OR Semaeostomeae OR Sessilia OR Sphacelariales OR Spionida OR Spongillida OR Syngnathiformes OR Terebellida OR Tilopteridales OR Trichoptera OR Ulotrichales OR Ulvales OR Unionida OR Vaucheriales]***

  OR

- higher taxon_Family ***[Acinetosporaceae OR Acipenseridae OR Acrochaetiaceae OR Acroloxidae OR Aeshnidae OR Ahnfeltiaceae OR Alismataceae OR Amblystegiaceae OR Ammodytidae OR Anguillidae OR Araceae OR Asellidae OR Balanidae OR Bangiaceae OR Bathyporeiidae OR Belonidae OR Bithyniidae OR Brachytheciaceae OR Calliergonaceae OR Calliopiidae OR Callithamniaceae OR Campanularidae OR Cardiidae OR Ceramiaceae OR Chaetiliidae OR Chaetophoraceae OR Characeae OR Chironomidae OR Chordaceae OR Chordariaceae OR Chrysomelidae OR Cladophoraceae OR Clupeidae OR Cobitidae OR Coenagrionidae OR Cordylophoridae OR Corophiidae OR Cottidae OR Crangonidae OR Cyaneidae OR Cyclopteridae OR***

Cyperaceae OR Cyprinidae OR Dreissenidae OR Dryopidae OR Dytiscidae OR Ecnomidae OR Ectocarpaceae OR Electridae OR Esocidae OR Fabriciidae OR Fissidentaceae OR Fontinalaceae OR Fucaceae OR Furcellariaceae OR Gadidae OR Gammaridae OR Gasterosteidae OR Gerridae OR Glossiphoniidae OR Gobiidae OR Halicryptidae OR Haloragaceae OR Halosiphonaceae OR Hildenbrandiaceae OR Hydridae OR Hydrobiidae OR Hydrocharitaceae OR Hydrophilidae OR Hydropsychidae OR Hydroptilidae OR Idoteidae OR Janiridae OR Kornmanniaceae OR Lepidostomatidae OR Leptoceridae OR Leuciscidae OR Libellulidae OR Limapontiidae OR Limnephilidae OR Liparidae OR Lithodermataceae OR Lotidae OR Lumpenidae OR Lymnaeidae OR Monostromataceae OR Murchisonellidae OR Myidae OR Mysidae OR Mytilidae OR Nepidae OR Nereididae OR Neritidae OR Olindiidae OR Osmeridae OR Palaemonidae OR Pallaseidae OR Panopeidae OR Percidae OR Petromyzontidae OR Pholidae OR Phryganeidae OR Phyllophoraceae OR Physidae OR Piscicolidae OR Pithophoraceae OR Planorbidae OR Plantaginaceae OR Pleuronectidae OR Plumatellidae OR Poaceae OR Polycentropodidae OR Polyidaceae OR Polynoidae OR Pontoporeiidae OR Portunidae OR Potamogetonaceae OR Prasiolaceae OR Protohydridae OR Psychomyiidae OR Psychrolutidae OR Ranunculaceae OR Rhodomelaceae OR Ruppiaceae OR Salmonidae OR Scophthalmidae OR Scytosiphonaceae OR Sphacelariaceae OR Spionidae OR Spongillidae OR Stypocaulaceae OR Syngnathidae OR Tateidae OR Tellinidae OR Terebellidae OR Tergipedidae OR Tetrastemmatidae OR Tincidae OR Ulmaridae OR Ulotrichaceae OR Ulvaceae OR Unionidae OR Valvatidae OR Varunidae OR Vaucheriaceae OR Victorellidae OR Viviparidae OR Zoarcidae OR Zosteraceae]

OR

- genus names ***[Halicryptus OR Abramis OR Acipenser OR Alburnus OR Ammodytes OR Anguilla OR Belone OR Blicca OR Carassius OR Clupea OR Cobitis OR Coregonus OR Cottus OR Cyclopterus OR Esox OR Gadus OR Gasterosteus OR Gobius OR Gymnocephalus OR Hyperoplus OR Leucaspius OR Leuciscus OR Liparis OR Lota OR Lumpenus OR Myoxocephalus OR Neogobius OR Nerophis OR Oncorhynchus OR Osmerus OR Pelecus OR Perca OR Pholis OR Phoxinus OR Platichthys OR Pomatoschistus OR Pungitius OR Rutilus OR Salmo OR Sander OR Scardinius OR Scophthalmus OR Spinachia OR Sprattus OR Syngnathus OR Taurulus OR Thymallus OR Tinca OR Vimba OR Zoarces OR Bangia OR Anodonta OR Cerastoderma OR Dreissena OR Limecola OR Mya OR Mytilopsis OR Mytilus OR Parvicardium OR Calliergon OR Fissidens OR Fontinalis OR Hygrohypnum OR Oxyrrhynchium OR Chara OR Nitella OR Nitellopsis OR Tolypella OR Chaetophora OR Cystobranchus OR Helobdella OR Piscicola OR Ephydatia OR Spongilla OR Aglaothamnion OR Ahnfeltia OR Ceramium OR Coccotylus OR Furcellaria OR Grania OR Hildenbrandia OR Phyllophora OR Polyides OR Polysiphonia OR Rhodochorton OR Rhodomela OR Acroloxus OR Alderia OR Anisus OR Bithynia OR Ecrobia OR Gyraulus OR Limapontia OR Lymnaea OR Peringia OR Physa OR Potamopyrgus OR Stagnicola OR Tenellia OR Theodoxus OR Valvata OR Viviparus OR Einhornia OR Victorella OR Amphibalanus OR Cyanophthalma OR Prostoma OR Cordylophora OR Gonothyraea OR Hydra OR Maeotias OR Protohydra OR Aeshna OR Agraylea OR Agrypnetes OR Agrypnia OR Allotrichia OR Aquarius OR Ceraclea OR Chironomus OR Clunio OR Coenagrion OR Cricotopus OR Cyrnus OR Dryops OR Ecnomus OR Erythromma OR Grammotaulius OR Halocladius OR Hydropsyche OR Hydroptila OR Hygrotus OR Ischnura OR Laccobius OR Lepidostoma OR Limnephilus OR Macroplea OR Nehalennia OR Oecetis OR Orthetrum OR Paratanytarsus OR Phryganea OR Polycentropus OR Psectrocladius OR Ranatra OR Sympetrum OR Tanytarsus OR Telmatogeton OR Tinodes OR Triaenodes OR Trichostegia OR Alisma OR Bolboschoenus OR Callitriche OR Eleocharis OR Leersia OR Lemna OR Myriophyllum OR Najas OR Phragmites OR Potamogeton OR Ranunculus OR Ruppia OR Schoenoplectus OR Stuckenia OR Zannichellia OR Zostera OR Apocorophium OR Asellus OR Bathyporeia OR Calliopius OR Carcinus OR Corophium OR Crangon OR Eriocheir OR Gammarus OR Hemimysis OR Idotea OR Jaera OR Leptocheirus OR***

Monoporeia OR Mysis OR Neomysis OR Palaemon OR Pallaseopsis OR Pontoporeia OR Praunus OR Rhithropanopeus OR Saduria OR Lampetra OR Battersia OR Chorda OR Dictyosiphon OR Ectocarpus OR Elachista OR Eudesme OR Fucus OR Halopteris OR Halosiphon OR Leathesia OR Protohalopteris OR Pseudolithoderma OR Pylaiella OR Scytosiphon OR Sphacelorbus OR Stictyosiphon OR Plumatella OR Bylgides OR Fabricia OR Fabriciola OR Hediste OR Manayunkia OR Marenzelleria OR Pygospio OR Terebellides OR Aurelia OR Cyanea OR Prasiola OR Rosenvingiella OR Acrosiphonia OR Aegagropila OR Blidingia OR Capsosiphon OR Chaetomorpha OR Cladophora OR Monostroma OR Percursaria OR Rhizoclonium OR Spongomorpha OR Ulothrix OR Ulva OR Urospora OR Vaucheria]

OR

- zooplankton taxon ***[Acartia OR Acineta OR Amoebozoa OR Anopla OR Arcella OR Askenasia OR Asplanchna OR Bosmina OR Brachionus OR Bryozoa OR Bylgides OR Bythotrephes OR Calanoida OR Calanus OR Centropages OR Cercopagis OR Ceriodaphnia OR Chaetognatha OR Chironomidae OR Chydorus OR Ciliophora OR Cladocera OR Cnidaria OR Collotheca OR Colurella OR Copepoda OR Coxliella OR Ctenophora OR Cyanea OR Cyclopoida OR Cyclops OR Daphnia OR Decapoda OR Diacyclops OR Diaphanosoma OR Diaptomus OR Didinium OR Difflugia OR Einhornia OR Euchlanis OR Eucyclops OR Eudiaptomus OR Eurytemora OR Evadne OR Filinia OR Fritillaria OR Harpacticoida OR Helicostomella OR Heliozoa OR Hyperia OR Hyperiidea OR Kellicottia OR Keratella OR Lacrymaria OR Lecane OR Leprotintinnus OR Leptodora OR Limnocalanus OR Macrocyclops OR Megacyclops OR Mertensia OR Microsetella OR Mnemiopsis OR Notholca OR Oikopleura OR Oithona OR Paracalanus OR Parasagitta OR Pleopis OR Pleurobrachia OR Podon OR Podonidae OR Polyarthra OR Polyphemus OR Pseudocalanus OR Radiosperma OR Rhithropanopeus OR Rotifera OR Sagitta OR Sessilida OR Strombidium OR Synchaeta OR Temora OR Thermocyclops OR Tintinnopsis OR Trichocerca OR Tunicata OR Vorticella OR Zoothamnium]***

OR

- synonyms/old names ***[Ulvopsis OR Enteromorpha OR Octodicerads OR Eurhynchium OR Audouinella OR Audouinella OR Sphacelari OR Stypocaulo OR Tetrastemma OR Prostomatella OR Cardium OR Macoma OR Hydrobia OR Embletonia OR Planorbis OR Laomedea OR Pelmatohydra OR Electra OR Nereis OR Harmothoe OR Triglopsis OR Stizostedion OR Acerina OR Gobiusculus OR Psetta OR Mesidotea OR Pallasea OR Leander OR Balanus OR Ylodes OR Trichostecia OR Coelambus]***

The Web of Science search string of Level 3 sub-query Q 3.2 is thus:

Q 3.2: TS=(Actinopteri OR Bangiophyceae OR Bivalvia OR Bryopsida OR Charophyceae OR Chlorophyceae OR Clitellata OR Demospongiae OR Florideophyceae OR Gastropoda OR Gymnolaemata OR Hexanauplia OR Hoplonemertea OR Hydrozoa OR Insecta OR Magnoliopsida OR Malacostraca OR Petromyzonti OR Phaeophyceae OR Phylactolaemata OR Polychaeta OR Scyphozoa OR Trebouxiophyceae OR Ulvophyceae OR Xanthophyceae OR Acipenseriformes OR Acrochaetiales OR Ahnfeltiales OR Alismatales OR Amphipoda OR Anguilliformes OR Anthoathecata OR Architaenioglossa OR Bangiales OR Beloniformes OR Carangiformes OR Cardiida OR Ceramiales OR Chaetophorales OR Charales OR Cheilostomata OR Cladophorales OR Clupeiformes OR Coleoptera OR Ctenostomatida OR Cypriniformes OR Decapoda OR Dicranales OR Diptera OR Ectocarpales OR Esociformes OR Fucales OR Gadiformes OR Gigartinales OR Gobiiformes OR Hemiptera OR Hildenbrandiales OR Hypnales OR Isopoda OR Lamiales OR Laminariales OR Leptothecata OR Limnomedusae OR Littorinimorpha OR Myoida OR Mysida OR Mytilida OR Nudibranchia OR Odonata OR Osmeriformes OR Perciformes OR Petromyzontiformes OR Phyllodocida OR Poales OR Prasiolales OR Ranunculales OR Rhynchobdellida OR Sabellida OR Salmoniformes OR

Saxifragales OR Semaeostomeae OR Sessilia OR Sphacelariales OR Spionida OR Spongillida OR Syngnathiformes OR Terebellida OR Tilopteridales OR Trichoptera OR Ulotrichales OR Ulvales OR Unionida OR Vaucheriales OR Acinetosporaceae OR Acipenseridae OR Acrochaetiaceae OR Acroloxidae OR Aeshnidae OR Ahnfeltiaceae OR Alismataceae OR Amblystegiaceae OR Ammodytidae OR Anguillidae OR Araceae OR Asellidae OR Balanidae OR Bangiaceae OR Bathyporeiidae OR Belonidae OR Bithyniidae OR Brachytheciaceae OR Calliergonaceae OR Calliopiidae OR Callithamniaceae OR Campanularidae OR Cardiidae OR Ceramiaceae OR Chaetiliidae OR Chaetophoraceae OR Characeae OR Chironomidae OR Chordaceae OR Chordariaceae OR Chrysomelidae OR Cladophoraceae OR Clupeidae OR Cobitidae OR Coenagrionidae OR Cordylophoridae OR Corophiidae OR Cottidae OR Crangonidae OR Cyaneidae OR Cyclopteridae OR Cyperaceae OR Cyprinidae OR Dreissenidae OR Dryopidae OR Dytiscidae OR Ecnomidae OR Ectocarpaceae OR Electridae OR Esocidae OR Fabriciidae OR Fissidentaceae OR Fontinalaceae OR Fucaceae OR Furcellariaceae OR Gadidae OR Gammaridae OR Gasterosteidae OR Gerridae OR Glossiphoniidae OR Gobiidae OR Halicryptidae OR Haloragaceae OR Halosiphonaceae OR Hildenbrandiaceae OR Hydridae OR Hydrobiidae OR Hydrocharitaceae OR Hydrophilidae OR Hydropsychidae OR Hydroptilidae OR Idoteidae OR Janiridae OR Kornmanniaceae OR Lepidostomatidae OR Leptoceridae OR Leuciscidae OR Libellulidae OR Limapontiidae OR Limnephilidae OR Liparidae OR Lithodermataceae OR Lotidae OR Lumpenidae OR Lymnaeidae OR Monostromataceae OR Murchisonellidae OR Myidae OR Mysidae OR Mytilidae OR Nepidae OR Nereididae OR Neritidae OR Olindiidae OR Osmeridae OR Palaemonidae OR Pallaseidae OR Panopeidae OR Percidae OR Petromyzontidae OR Pholidae OR Phryganeidae OR Phyllophoraceae OR Physidae OR Piscicolidae OR Pithophoraceae OR Planorbidae OR Plantaginaceae OR Pleuronectidae OR Plumatellidae OR Poaceae OR Polycentropodidae OR Polyidaceae OR Polynoidae OR Pontoporeiidae OR Portunidae OR Potamogetonaceae OR Prasiolaceae OR Protohydridae OR Psychomyiidae OR Psychrolutidae OR Ranunculaceae OR Rhodomelaceae OR Ruppiaceae OR Salmonidae OR Scophthalmidae OR Scytosiphonaceae OR Sphacelariaceae OR Spionidae OR Spongillidae OR Stypocaulaceae OR Syngnathidae OR Tateidae OR Tellinidae OR Terebellidae OR Tergipedidae OR Tetrastemmatidae OR Tincidae OR Ulmaridae OR Ulotrichaceae OR Ulvaceae OR Unionidae OR Valvatidae OR Varunidae OR Vaucheriaceae OR Victorellidae OR Viviparidae OR Zoarcidae OR Zosteraceae OR Halicryptus OR Abramis OR Acipenser OR Alburnus OR Ammodytes OR Anguilla OR Belone OR Blicca OR Carassius OR Clupea OR Cobitis OR Coregonus OR Cottus OR Cyclopterus OR Esox OR Gadus OR Gasterosteus OR Gobius OR Gymnocephalus OR Hyperoplus OR Leucaspius OR Leuciscus OR Liparis OR Lota OR Lumpenus OR Myoxocephalus OR Neogobius OR Nerophis OR Oncorhynchus OR Osmerus OR Pelecus OR Perca OR Pholis OR Phoxinus OR Platichthys OR Pomatoschistus OR Pungitius OR Rutilus OR Salmo OR Sander OR Scardinius OR Scophthalmus OR Spinachia OR Sprattus OR Syngnathus OR Taurulus OR Thymallus OR Tinca OR Vimba OR Zoarces OR Bangia OR Anodonta OR Cerastoderma OR Dreissena OR Limecola OR Mya OR Mytilopsis OR Mytilus OR Parvicardium OR Calliergon OR Fissidens OR Fontinalis OR Hygrohypnum OR Oxyrrhynchium OR Chara OR Nitella OR Nitellopsis OR Tolypella OR Chaetophora OR Cystobranchus OR Helobdella OR Piscicola OR Ephydatia OR Spongilla OR Aglaothamnion OR Ahnfeltia OR Ceramium OR Coccotylus OR Furcellaria OR Grania OR Hildenbrandia OR Phyllophora OR Polyides OR Polysiphonia OR Rhodochorton OR Rhodomela OR Acroloxus OR Alderia OR Anisus OR Bithynia OR Ecrobia OR Gyraulus OR Limapontia OR Lymnaea OR Peringia OR Physa OR Potamopyrgus OR Stagnicola OR Tenellia OR Theodoxus OR Valvata OR Viviparus OR Einhornia OR Victorella OR Amphibalanus OR Cyanophthalma OR Prostoma OR Cordylophora OR Gonothyraea OR Hydra OR Maeotias

OR Protohydra OR Aeshna OR Agraylea OR Agrypnetes OR Agrypnia OR Allotrichia OR Aquarius OR Ceraclea OR Chironomus OR Clunio OR Coenagrion OR Cricotopus OR Cyrnus OR Dryops OR Ecnomus OR Erythromma OR Grammotaulius OR Halocladius OR Hydropsyche OR Hydroptila OR Hygrotus OR Ischnura OR Laccobius OR Lepidostoma OR Limnephilus OR Macroplea OR Nehalennia OR Oecetis OR Orthetrum OR Paratanytarsus OR Phryganea OR Polycentropus OR Psectrocladius OR Ranatra OR Sympetrum OR Tanytarsus OR Telmatogeton OR Tinodes OR Triaenodes OR Trichostegia OR Alisma OR Bolboschoenus OR Callitriche OR Eleocharis OR Leersia OR Lemna OR Myriophyllum OR Najas OR Phragmites OR Potamogeton OR Ranunculus OR Ruppia OR Schoenoplectus OR Stuckenia OR Zannichellia OR Zostera OR Apocorophium OR Asellus OR Bathyporeia OR Calliopius OR Carcinus OR Corophium OR Crangon OR Eriocheir OR Gammarus OR Hemimysis OR Idotea OR Jaera OR Leptocheirus OR Monoporeia OR Mysis OR Neomysis OR Palaemon OR Pallaseopsis OR Pontoporeia OR Praunus OR Rhithropanopeus OR Saduria OR Lampetra OR Battersia OR Chorda OR Dictyosiphon OR Ectocarpus OR Elachista OR Eudesme OR Fucus OR Halopteris OR Halosiphon OR Leathesia OR Protohalopteris OR Pseudolithoderma OR Pylaiella OR Scytosiphon OR Sphacelorbus OR Stictyosiphon OR Plumatella OR Bylgides OR Fabricia OR Fabriciola OR Hediste OR Manayunkia OR Marenzelleria OR Pygospio OR Terebellides OR Aurelia OR Cyanea OR Prasiola OR Rosenvingiella OR Acrosiphonia OR Aegagropila OR Blidingia OR Capsosiphon OR Chaetomorpha OR Cladophora OR Monostroma OR Percursaria OR Rhizoclonium OR Spongomorpha OR Ulothrix OR Ulva OR Urospora OR Vaucheria OR Acartia OR Acineta OR Amoebozoa OR Anopla OR Arcella OR Askenasia OR Asplanchna OR Bosmina OR Brachionus OR Bryozoa OR Bylgides OR Bythotrephes OR Calanoida OR Calanus OR Centropages OR Cercopagis OR Ceriodaphnia OR Chaetognatha OR Chironomidae OR Chydorus OR Ciliophora OR Cladocera OR Cnidaria OR Collotheca OR Colurella OR Copepoda OR Coxliella OR Ctenophora OR Cyanea OR Cyclopoida OR Cyclops OR Daphnia OR Decapoda OR Diacyclops OR Diaphanosoma OR Diaptomus OR Didinium OR Difflugia OR Einhornia OR Euchlanis OR Eucyclops OR Eudiaptomus OR Eurytemora OR Evadne OR Filinia OR Fritillaria OR Harpacticoida OR Helicostomella OR Heliozoa OR Hyperia OR Hyperiidea OR Kellicottia OR Keratella OR Lacrymaria OR Lecane OR Leprotintinnus OR Leptodora OR Limnocalanus OR Macrocyclops OR Megacyclops OR Mertensia OR Microsetella OR Mnemiopsis OR Notholca OR Oikopleura OR Oithona OR Paracalanus OR Parasagitta OR Pleopis OR Pleurobrachia OR Podon OR Podonidae OR Polyarthra OR Polyphemus OR Pseudocalanus OR Radiosperma OR Rhithropanopeus OR Rotifera OR Sagitta OR Sessilida OR Strombidium OR Synchaeta OR Temora OR Thermocyclops OR Tintinnopsis OR Trichocerca OR Tunicata OR Vorticella OR Zoothamnium OR Ulvopsis OR Enteromorpha OR Octodicerads OR Eurhynchium OR Audouinella OR Audouinella OR Sphacelari OR Stypocaulo OR Tetrastemma OR Prostomatella OR Cardium OR Macoma OR Hydrobia OR Embletonia OR Planorbis OR Laomedea OR Pelmatohydra OR Electra OR Nereis OR Harmothoe OR Triglopsis OR Stizostedion OR Acerina OR Gobiusculus OR Psetta OR Mesidotea OR Pallasea OR Leander OR Balanus OR Ylodes OR Trichostecia OR Coelambus)

The Scopus search string of Level 3 sub-query Q 3.2 is thus:

Q 3.2: TITLE-ABS-KEY(Actinopteri OR Bangiophyceae OR Bivalvia OR Bryopsida OR Charophyceae OR Chlorophyceae OR Clitellata OR Demospongiae OR Florideophyceae OR Gastropoda OR Gymnolaemata OR Hexanauplia OR Hoplonemertea OR Hydrozoa OR Insecta OR Magnoliopsida OR Malacostraca OR Petromyzonti OR Phaeophyceae OR Phylactolaemata OR Polychaeta OR Scyphozoa OR Trebouxiophyceae OR Ulvophyceae OR Xanthophyceae OR Acipenseriformes OR

Acrochaetiales OR Ahnfeltiales OR Alismatales OR Amphipoda OR Anguilliformes OR Anthoathecata OR Architaenioglossa OR Bangiales OR Beloniformes OR Carangiformes OR Cardiida OR Ceramiales OR Chaetophorales OR Charales OR Cheilostomata OR Cladophorales OR Clupeiformes OR Coleoptera OR Ctenostomatida OR Cypriniformes OR Decapoda OR Dicranales OR Diptera OR Ectocarpales OR Esociformes OR Fucales OR Gadiformes OR Gigartinales OR Gobiiformes OR Hemiptera OR Hildenbrandiales OR Hypnales OR Isopoda OR Lamiales OR Laminariales OR Leptothecata OR Limnomedusae OR Littorinimorpha OR Myoida OR Mysida OR Mytilida OR Nudibranchia OR Odonata OR Osmeriformes OR Perciformes OR Petromyzontiformes OR Phyllodocida OR Poales OR Prasiolales OR Ranunculales OR Rhynchobdellida OR Sabellida OR Salmoniformes OR Saxifragales OR Semaeostomeae OR Sessilia OR Sphacelariales OR Spionida OR Spongillida OR Syngnathiformes OR Terebellida OR Tilopteridales OR Trichoptera OR Ulotrichales OR Ulvales OR Unionida OR Vaucheriales OR Acinetosporaceae OR Acipenseridae OR Acrochaetiaceae OR Acroloxidae OR Aeshnidae OR Ahnfeltiaceae OR Alismataceae OR Amblystegiaceae OR Ammodytidae OR Anguillidae OR Araceae OR Asellidae OR Balanidae OR Bangiaceae OR Bathyporeiidae OR Belonidae OR Bithyniidae OR Brachytheciaceae OR Calliergonaceae OR Calliopiidae OR Callithamniaceae OR Campanularidae OR Cardiidae OR Ceramiaceae OR Chaetiliidae OR Chaetophoraceae OR Characeae OR Chironomidae OR Chordaceae OR Chordariaceae OR Chrysomelidae OR Cladophoraceae OR Clupeidae OR Cobitidae OR Coenagrionidae OR Cordylophoridae OR Corophiidae OR Cottidae OR Crangonidae OR Cyaneidae OR Cyclopteridae OR Cyperaceae OR Cyprinidae OR Dreissenidae OR Dryopidae OR Dytiscidae OR Ecnomidae OR Ectocarpaceae OR Electridae OR Esocidae OR Fabriciidae OR Fissidentaceae OR Fontinalaceae OR Fucaceae OR Furcellariaceae OR Gadidae OR Gammaridae OR Gasterosteidae OR Gerridae OR Glossiphoniidae OR Gobiidae OR Halicryptidae OR Haloragaceae OR Halosiphonaceae OR Hildenbrandiaceae OR Hydridae OR Hydrobiidae OR Hydrocharitaceae OR Hydrophilidae OR Hydropsychidae OR Hydroptilidae OR Idoteidae OR Janiridae OR Kornmanniaceae OR Lepidostomatidae OR Leptoceridae OR Leuciscidae OR Libellulidae OR Limapontiidae OR Limnephilidae OR Liparidae OR Lithodermataceae OR Lotidae OR Lumpenidae OR Lymnaeidae OR Monostromataceae OR Murchisonellidae OR Myidae OR Mysidae OR Mytilidae OR Nepidae OR Nereididae OR Neritidae OR Olindiidae OR Osmeridae OR Palaemonidae OR Pallaseidae OR Panopeidae OR Percidae OR Petromyzontidae OR Pholidae OR Phryganeidae OR Phyllophoraceae OR Physidae OR Piscicolidae OR Pithophoraceae OR Planorbidae OR Plantaginaceae OR Pleuronectidae OR Plumatellidae OR Poaceae OR Polycentropodidae OR Polyidaceae OR Polynoidae OR Pontoporeiidae OR Portunidae OR Potamogetonaceae OR Prasiolaceae OR Protohydridae OR Psychomyiidae OR Psychrolutidae OR Ranunculaceae OR Rhodomelaceae OR Ruppiaceae OR Salmonidae OR Scophthalmidae OR Scytosiphonaceae OR Sphacelariaceae OR Spionidae OR Spongillidae OR Stypocaulaceae OR Syngnathidae OR Tateidae OR Tellinidae OR Terebellidae OR Tergipedidae OR Tetrastemmatidae OR Tincidae OR Ulmaridae OR Ulotrichaceae OR Ulvaceae OR Unionidae OR Valvatidae OR Varunidae OR Vaucheriaceae OR Victorellidae OR Viviparidae OR Zoarcidae OR Zosteraceae OR Halicryptus OR Abramis OR Acipenser OR Alburnus OR Ammodytes OR Anguilla OR Belone OR Blicca OR Carassius OR Clupea OR Cobitis OR Coregonus OR Cottus OR Cyclopterus OR Esox OR Gadus OR Gasterosteus OR Gobius OR Gymnocephalus OR Hyperoplus OR Leucaspius OR Leuciscus OR Liparis OR Lota OR Lumpenus OR Myoxocephalus OR Neogobius OR Nerophis OR Oncorhynchus OR Osmerus OR Pelecus OR Perca OR Pholis OR Phoxinus OR Platichthys OR Pomatoschistus OR Pungitius OR Rutilus OR Salmo OR Sander OR Scardinius OR Scophthalmus OR Spinachia OR Sprattus OR Syngnathus OR Taurulus OR Thymallus OR Tinca OR Vimba OR Zoarces OR Bangia OR Anodonta OR Cerastoderma OR Dreissena OR Limecola OR Mya OR Mytilopsis OR Mytilus OR Parvicardium OR Calliergon OR Fissidens OR Fontinalis OR Hygrohypnum OR Oxyrrhynchium OR Chara OR Nitella OR Nitellopsis OR Tolypella OR Chaetophora OR Cystobranchus OR Helobdella OR

Piscicola OR Ephydatia OR Spongilla OR Aglaothamnion OR Ahnfeltia OR Ceramium OR Coccotylus OR Furcellaria OR Grania OR Hildenbrandia OR Phyllophora OR Polyides OR Polysiphonia OR Rhodochorton OR Rhodomela OR Acroloxus OR Alderia OR Anisus OR Bithynia OR Ecrobia OR Gyraulus OR Limapontia OR Lymnaea OR Peringia OR Physa OR Potamopyrgus OR Stagnicola OR Tenellia OR Theodoxus OR Valvata OR Viviparus OR Einhornia OR Victorella OR Amphibalanus OR Cyanophthalma OR Prostoma OR Cordylophora OR Gonothyraea OR Hydra OR Maeotias OR Protohydra OR Aeshna OR Agraylea OR Agrypnetes OR Agrypnia OR Allotrichia OR Aquarius OR Ceraclea OR Chironomus OR Clunio OR Coenagrion OR Cricotopus OR Cyrnus OR Dryops OR Ecnomus OR Erythromma OR Grammotaulius OR Halocladius OR Hydropsyche OR Hydroptila OR Hygrotus OR Ischnura OR Laccobius OR Lepidostoma OR Limnephilus OR Macroplea OR Nehalennia OR Oecetis OR Orthetrum OR Paratanytarsus OR Phryganea OR Polycentropus OR Psectrocladius OR Ranatra OR Sympetrum OR Tanytarsus OR Telmatogeton OR Tinodes OR Triaenodes OR Trichostegia OR Alisma OR Bolboschoenus OR Callitriche OR Eleocharis OR Leersia OR Lemna OR Myriophyllum OR Najas OR Phragmites OR Potamogeton OR Ranunculus OR Ruppia OR Schoenoplectus OR Stuckenia OR Zannichellia OR Zostera OR Apocorophium OR Asellus OR Bathyporeia OR Calliopius OR Carcinus OR Corophium OR Crangon OR Eriocheir OR Gammarus OR Hemimysis OR Idotea OR Jaera OR Leptocheirus OR Monoporeia OR Mysis OR Neomysis OR Palaemon OR Pallaseopsis OR Pontoporeia OR Praunus OR Rhithropanopeus OR Saduria OR Lampetra OR Battersia OR Chorda OR Dictyosiphon OR Ectocarpus OR Elachista OR Eudesme OR Fucus OR Halopteris OR Halosiphon OR Leathesia OR Protohalopteris OR Pseudolithoderma OR Pylaiella OR Scytosiphon OR Sphacelorbus OR Stictyosiphon OR Plumatella OR Bylgides OR Fabricia OR Fabriciola OR Hediste OR Manayunkia OR Marenzelleria OR Pygospio OR Terebellides OR Aurelia OR Cyanea OR Prasiola OR Rosenvingiella OR Acrosiphonia OR Aegagropila OR Blidingia OR Capsosiphon OR Chaetomorpha OR Cladophora OR Monostroma OR Percursaria OR Rhizoclonium OR Spongomorpha OR Ulothrix OR Ulva OR Urospora OR Vaucheria OR Acartia OR Acineta OR Amoebozoa OR Anopla OR Arcella OR Askenasia OR Asplanchna OR Bosmina OR Brachionus OR Bryozoa OR Bylgides OR Bythotrephes OR Calanoida OR Calanus OR Centropages OR Cercopagis OR Ceriodaphnia OR Chaetognatha OR Chironomidae OR Chydorus OR Ciliophora OR Cladocera OR Cnidaria OR Collotheca OR Colurella OR Copepoda OR Coxliella OR Ctenophora OR Cyanea OR Cyclopoida OR Cyclops OR Daphnia OR Decapoda OR Diacyclops OR Diaphanosoma OR Diaptomus OR Didinium OR Difflugia OR Einhornia OR Euchlanis OR Eucyclops OR Eudiaptomus OR Eurytemora OR Evadne OR Filinia OR Fritillaria OR Harpacticoida OR Helicostomella OR Heliozoa OR Hyperia OR Hyperiidea OR Kellicottia OR Keratella OR Lacrymaria OR Lecane OR Leprotintinnus OR Leptodora OR Limnocalanus OR Macrocyclops OR Megacyclops OR Mertensia OR Microsetella OR Mnemiopsis OR Notholca OR Oikopleura OR Oithona OR Paracalanus OR Parasagitta OR Pleopis OR Pleurobrachia OR Podon OR Podonidae OR Polyarthra OR Polyphemus OR Pseudocalanus OR Radiosperma OR Rhithropanopeus OR Rotifera OR Sagitta OR Sessilida OR Strombidium OR Synchaeta OR Temora OR Thermocyclops OR Tintinnopsis OR Trichocerca OR Tunicata OR Vorticella OR Zoothamnium OR Ulvopsis OR Enteromorpha OR Octodicerads OR Eurhynchium OR Audouinella OR Audouinella OR Sphacelari OR Stypocaulo OR Tetrastemma OR Prostomatella OR Cardium OR Macoma OR Hydrobia OR Embletonia OR Planorbis OR Laomedea OR Pelmatohydra OR Electra OR Nereis OR Harmothoe OR Triglopsis OR Stizostedion OR Acerina OR Gobiusculus OR Psetta OR Mesidotea OR Pallasea OR Leander OR Balanus OR Ylodes OR Trichostecia OR Coelambus)

1.4 Literature search results

Search records from the two searches with same search queries were retrieved from WoS (All databases) and Scopus search database platforms. The number of records retrieved were 3029 for WoS and 2115 for

Scopus, rendering a total of 5144 raw records. Below are presented the bibliographic results of the literature search (Table S1 and Table S2).

Table S1. Bibliographic records based on a systematic literature search on the study question "How is marine nature/biodiversity loss expressed in shallow coastal habitats (in Finland)?" conducted in the literature database Web of Science on 28th October 2022. The hits in sub-queries in Level 1 are hits for these isolated sub-queries. Hits in sub-queries in Level 2 are their contribution to Query 1, and in Level 3 their contribution to Query 2.

**Search Level**	**Search Level info**	**Queries**	**Hits**
1	Level 1 dissects the study question into three main elements to be answered/targeted by the literature search: Area and Environment, Research subject, and Phenomenon. The query at Level 1 can be seen as the core apex of the search. The Level 1 Query is composed of three sub-queries corresponding to the three main elements of the search as Q 1.1 – Area and Environment, Q 1.2 – Research subject, and Q 1.3 – Phenomenon.	*Query 1: TS=(**Q 1.1** AND **Q 1.2** AND **Q 1.3**)*	**2 749**
		Q 1.1 – Area and Environment	3 945
		Q 1.2 – Research subject	53 171 692
		Q 1.3 – Phenomenon	58 267 151
2	Level 2 aims at widening the search to cover more potentially relevant papers. The Level 2 Query builds upon Query 1, by widening the concept of the research subject "nature", for which "biodiversity" was used as the quality element in focus, through adding Level 2 sub-queries to the Q 1.2 sub-query term of Query 1. The Level 2 Query is composed of two sub-queries both aiming at including more of the relevant aspects of nature and biodiversity to the search. The sub-queries are Q 2.1 – Additional aspects of biodiversity and Q 2.2 – Main organism groups / communities/ assemblages.	*Query 2: TS=(Q 1.1 AND (Q 1.2 OR **Q 2.1** OR **Q 2.2**) AND Q 1.3)*	**2 981**
		Q 2.1 – Additional aspects of biodiversity and nature loss	+209
		Q 2.2 – Main organism groups/communities/assemblages	+46
3	Level 3 aims at even further widening the search to cover more potentially relevant papers. The Level 3 Query builds upon Query 2 from the previous Level 2, by elaborating the concept of "biodiversity", through adding Level 3 sub-queries to the Q 1.2, Q 2.1, and Q 2.2 sub-query terms of Query 2. At this Level species and higher taxonomic common names and scientific nomenclature is added to the search.	*Query 3: TS=(Q 1.1 AND (Q 1.2 OR Q 2.1 OR Q 2.2 OR **Q 3.1** OR **Q 3.2**) AND Q 1.3)*	**3 029**
		Q 3.1 – Organism and taxon common names	+48

	The Level 3 Query is composed of two sub-queries all aiming at including more of the relevant aspects of nature and biodiversity to the search. These sub-queries are Q 3.1 – Organism and taxon common names and Q 3.2 – Scientific nomenclature of taxon.	Q 3.2 – Scientific nomenclature of taxon	+0

Table S2. Bibliographic records based on a systematic literature search on the study question "How is marine nature/biodiversity loss expressed in shallow coastal habitats (in Finland)?" conducted in the literature database Scopus on 6th October 2022. The hits in sub-queries in Level 1 are hits for these isolated sub-queries. Hits in sub-queries in Level 2 are their contribution to Query 1, and in Level 3 their contribution to Query 2.

**Search Level**	**Search Level info**	**Queries**	**Hits**
1	Level 1 dissects the study question into three main elements to be answered/targeted by the literature search: Area and Environment, Research subject, and Phenomenon. The query at Level 1 can be seen as the core apex of the search. The Level 1 Query is composed of three sub-queries corresponding to the three main elements of the search as Q 1.1 – Area and Environment, Q 1.2 – Research subject, and Q 1.3 – Phenomenon.	*Query 1: (TITLE-ABS-KEY=(**Q 1.1** AND **Q 1.2** AND **Q 1.3**))*	**1 641**
		Q 1.1 – Area and Environment	3 360
		Q 1.2 – Research subject	28 092 811
		Q 1.3 – Phenomenon	42 564 153
2	Level 2 aims at widening the search to cover more potentially relevant papers. The Level 2 Query builds upon Query 1, by widening the concept of the research subject "nature", for which "biodiversity" was used as the quality element in focus, through adding Level 2 sub-queries to the Q 1.2 sub-query term of Query 1. The Level 2 Query is composed of two sub-queries both aiming at including more of the relevant aspects of nature and biodiversity to the search. The sub-queries are Q 2.1 – Additional aspects of biodiversity and Q 2.2 – Main organism groups / communities/ assemblages.	*Query 2: (TITLE-ABS-KEY=(Q 1.1 AND (Q 1.2 OR **Q 2.1** OR **Q 2.2**) AND Q 1.3))*	**2 017**
		Q 2.1 – Additional aspects of biodiversity and nature loss	+328
		Q 2.2 – Main organism groups/communities/assemblages	+111
3	Level 3 aims at even further widening the search to cover more potentially relevant papers. The Level 3 Query builds upon Query 2 from the previous Level 2, by elaborating the concept of "biodiversity", through adding Level 3 sub-queries to the Q 1.2, Q 2.1, and Q 2.2 sub-query terms of Query 2. At this Level species and higher taxonomic common names and scientific nomenclature is added to the search.<br>The Level 3 Query is composed of two sub-queries all aiming at including more of the relevant aspects of nature and biodiversity to the search. These sub-queries are Q 3.1 – Organism and taxon common names and Q 3.2 – Scientific nomenclature of taxon.	*Query 3: (TITLE-ABS-KEY=(Q 1.1 AND (Q 1.2 OR Q 2.1 OR Q 2.2 OR Q 3.1 OR Q 3.2) AND Q 1.3))*	**2 115**
		Q 3.1 – Organism and taxon common names	+96
		Q 3.2 – Scientific nomenclature of taxon	+7

2 Screening of search records and data

All search records (5144) from both searches were imported to Zotero software for reference handling. In Zotero, first all duplicate records were identified and removed one by one by using the program's functionality for detecting duplicates. Altogether 1631 duplicates were identified and removed leaving with 3513 unique records.

To identify the final articles to be included, the search results underwent a two-stage screening process, first based on the title and abstract, and then based on the full text. The screening was conducted according to set eligibility criteria. The primary eligibility criterion was that the article contains results from a temporal trend study or a point-in-time comparison, meaning it includes data that shows or could show changes in marine biodiversity in Finland's shallow coastal waters. Both qualitative and quantitative results were accepted. The eligibility criteria were as follows:

- **Temporal:** The observation period must be at least 4 years long, with no predefined start year.
- **Spatial:** All marine areas belonging to Finland and their shallow coastal areas, defined as underwater areas from the surface to the bottom to the lower boundary of the photic zone (approximately 10 meters, though case-by-case evaluation is used to include potentially relevant data rather than exclude it).
- **Organism groups:** All aquatic organisms except for waterfowl, mammals, and reptiles.
- **Changes in Biodiversity and factors of biodiversity loss:** Biodiversity changes are broadly interpreted as any temporal changes in any element of biodiversity. Biodiversity loss encompasses all such negative changes, including but not limited to local extinction of species or populations, declines in population abundance, changes in species occurrence and distribution, changes in communities, and altered or degraded ecological functions or other ecosystem level changes.
- **Data:** Primarily field study data derived from temporal observations, sampling, measurements, and monitoring conducted in nature, excluding pure experimental studies or future scenario modeling.

Articles containing relevant evidence meeting these criteria for biodiversity loss were ultimately selected for inclusion. In addition to the articles found in the literature search a few articles were manually added. To facilitate the relative comparison of expressions of biodiversity loss (e.g., between marine areas, littoral biotopes, or organism groups), the search results also recorded observations from relevant studies that could have indicated biodiversity loss but instead showed positive changes or no changes at all. In examining the results, references are made to the entire included dataset for all research findings, and specifically to the biodiversity loss dataset or the studies indicating biodiversity loss for the relevant subset of data.

Following an initial relevance screening based on title and abstract, 290 articles were included as likely relevant. These articles underwent a second, more detailed full-text screening. Out of the 290 articles in the second screening phase, the full-text versions of 10 articles were unavailable, preventing their assessment and leading to their exclusion. Of the articles assessed in the second phase, a total of 79 were considered to have potentially relevant evidence of changes in biological diversity. Of these, 61 articles contained one or more research observations indicating biodiversity loss, and 18 articles contained only evidence of positive changes or temporal data with no clear changes. Among the articles with multiple research observations, 43 contained evidence of both biodiversity loss and positive changes or no changes at all. Articles deemed irrelevant or not meeting the eligibility criteria (201 in total) were either discarded or marked as otherwise relevant references for possible future use.

In addition to the articles that passed the literature search screening, 11 relevant articles were found from the reference lists of included articles or through other manual searches (e.g., unstructured searches with Google and Google Scholar). Of these, 10 contained evidence of biodiversity loss, and 1 contained evidence of positive changes or no changes at all. Ultimately, the search procedure yielded 90 unique articles for

inclusion, of which 71 contained evidence of biodiversity loss, and 19 contained evidence of only positive changes or no changes at all.

3 Processing and analysis of research data

From the included articles, research observations related to changes in biodiversity were extracted. A research observation was considered if it was discernable and separately extractable. Each article could contain one or more research observations related to changes in biodiversity. If multiple clearly identical observations based on the same dataset were found, only one was included in the final set of research observations. However, if the identity or possible similarity of observations was unclear, observations were then rather included than removed from the list. Each research observation related to changes in biodiversity was linked to accompanying information in the literature or indirectly revealed information, such as temporal and geographical coverage, environmental characteristics, biotopes, and taxonomic details.

The research observations were categorized into the following broad biodiversity topics: genetic, population, community, ecosystem level and other complex changes. For a more detailed classification, different biodiversity elements were identified and categorized in biodiversity categories (only shown in supplementary results) as follows: Biotic abundance and biomass = organism count, biomass, density, and coverage; Taxon occurrence = presence/absence of a species, frequency of occurrence, and distribution; Community structure = species composition and abundance, dominance ratios of species, biodiversity indices, trophic levels, and community size structure; Individual traits and qualities = growth, sexual maturity, reproductive output, body composition, and body disorders; Population structure = age, sex, and size distribution; Ecosystem changes = various changes at the ecosystem level; Ecological functions = functional diversity and ecological processes; 'Other' category = genetic differentiation, species/population general condition, and species/population phenology.

The type of change (or main type of expression) indicating biodiversity loss was defined as follows: change, decrease, local disappearance, increase, no improvement, and multiple (complex) changes. The change type 'decrease' also included e.g. the following changes: decline, reduction, weakening, diminishing, or slowing down. The biodiversity elements and the type of change constituted the expression form of biodiversity loss for each research observation, for example, "change in species distribution" or "decrease in population size."

The collected research observations were processed and analyzed with respect to expression forms of biodiversity loss and other characteristics of the dataset, and results were examined as a whole and utilizing different categorizations. It is essential to note that when presenting results, biodiversity loss findings based on research literature are presented in two ways: 1) as the number of research observations indicating biodiversity loss and 2) as the relative proportion of observations indicating biodiversity loss among all included data for each assessable categorization of interest (= 'relative occurrence').

Research observations indicating biodiversity loss were linked to drivers of change to the extent that such information was available. Causes of biodiversity loss were classified as probable, possible, or evidence based. Evidence-based causes were those whose impact on an element of biodiversity had been specifically studied. The significance of causes was assessed as the occurrence frequency percentage in the datasets of research observations indicating biodiversity loss. A specific biodiversity loss observation may be associated with several causes so each driver could attain an occurrence frequency between 0 and 100 percent. The found causes were denoted as follows: eutrophication, climate change, loss/degradation of habitats, variations in key environmental conditions, direct human-induced mortality (fishing), invasive species, reduction in eutrophication, acidification, physical disturbance of seabed, harmful substances, stocking of fish species, indirect ecological interactions, and artificial warming of water.

Examination of results was conducted also separately for each organism group, biotope, and marine area, thereby highlighting the key findings from three complementary perspectives. The ‘knowledge base’ (= number of research observations), the ‘relative occurrence of biodiversity loss’ (= proportion of biodiversity loss evidence within all included data for each organism group, biotope, or sea area grouping), and the ‘number of expressions forms’ (= the number of different expression forms of biodiversity loss observed in the scientific literature) of biodiversity loss were classified for the purpose of summarizing. The classification of the ‘knowledge base’ specific to organism groups, biotopes, and marine areas was based on rounded percentage shares of the number of research observations in the whole dataset and classified as sparse (0–9%, "orange"), moderate (10–24 %, "yellow"), or abundant (≥25%, "green"). The classification of the relative occurrence of biodiversity loss was based on the rounded percentage of biodiversity loss observations relative to all observations for each group or grouping and was classified as low (0–32%, "green"), moderate (33–67%, "yellow"), or high (≥68%, "orange"). The number of biodiversity loss expression forms was classified for each group or grouping based on the identified number of different expression forms of biodiversity loss and was classified as low (0–9 forms, "green"), moderate (10–19 forms, "yellow"), or high (≥20 forms, "orange").

4 Outcomes from the literature search and supplemental results of the assessment

Based on the literature search and the eligibility screening a total of 90 relevant research articles were found and included (Table S3). The included articles originated from 48 different scientific journals (Table S4). In total, 774 research observations of relevance were found, of which 427 observations indicated biodiversity loss, and the rest were evidence of positive changes or no changes at all. The included research observations were mainly derived from research articles (757 pieces) but also from a few literature reviews (17 pieces). The data used in the included articles and observations varied greatly, encompassing primary sampling data from studies, raw data from previous research, literature information, national monitoring and inventory data, official statistical data, and monitoring information from research projects, municipalities, and industry.

Most of the observations consisted of changes between two comparison years or periods (501 pieces, 65%), a small portion came from current state reviews (46 pieces, 6%), and the rest from multi-year time series (227 pieces, 30%). The included articles spanned the past 40 years, with publication years ranging from 1984 to 2023. The number of included articles and research observations per publication year and decade are presented in Figure S1. The average publication rate of the included articles was approximately 2.3 articles per year. The publication rate of articles has varied greatly between years but has accelerated from the 1980s to the present: there are five articles from the 1980s, 14 from the 1990s, 26 from the 2000s, 30 from the 2010s, and 15 articles from the 2020s. Similarly, the distribution of research observations by publication year showed inter-annual variation and an increasing number of observations over time: there were 11 observations from the 1980s, 107 from the 1990s, 211 from the 2000s, 211 from the 2010s, and 234 from the 2020s. On average, there were 19.4 observations per year.

The temporal coverage of the included datasets varied between 4 and 250 years, with an average of 37 years (± 28 years). The majority of datasets containing evidence of biodiversity loss covered a period of 26–50 years (37%, Figure S2A), and when examined in ten-year intervals, the dominant temporal coverage was 11–20 years (25%). The temporal placement of the research observations also varied widely. The start years of the comparisons ranged from 1800 to 2010, and the end years ranged from 1955 to 2022. Over half (58%) of the research findings extended at least until 2005 (Figure S2B), and about 74% of the biodiversity loss datasets included newer data that extended beyond the turn of the millennium. When examined in terms of temporal coverage, the relative occurrence of biodiversity loss observations within the entire dataset was highest (88%) in the datasets with the most extensive temporal coverage, over 75 years, but otherwise varied between approximately 50 and 60% (Figure S3A). The relative occurrence of biodiversity loss observations was highest in the earliest datasets (100% when the end years of the datasets were between 1955–1964), although there were very few of these observations (5 in total). After that, the relative occurrence of biodiversity loss varied modestly between approximately 43 and 64% (Figure S3B).

In addition to the results showing the distribution of the research observations relevant to biodiversity change across organism groups, biotopes and geographical areas presented in the main article, the data was also categorized and assessed by the identified biodiversity categories (Figure S4). Of these categories, species abundance and biomass, and taxon occurrence were most abundant among the observations indicating biodiversity loss (38% and 35%, respectively), while the categories representing ecological functions and ecosystem change as well as the 'other' category (e.g. genetic differentiation and species/population status), were the rarest (Figure S4). The relative occurrence of biodiversity loss observations was lowest (44%) in the species abundance category and highest (91%) in the 'other' category (Figure S4).

There was a clear correlation between the number of expression forms and the number of research observations both regarding marine areas and organism groups (Pearson's correlation: 0.85–0.87; $p < 0.001$; Figure S5).

The occurrence frequency (%) of drivers of biodiversity loss among research observations is presented by organism groups, littoral biotopes, and geographical areas in Figures S6–S8. When analyzed by organism groups, the occurrence frequency of different drivers varied considerably between organism groups (Figure S6). The role of eutrophication was evident for most organism groups, and it appeared in all or nearly all (95–100%) of the evidence concerning biodiversity loss in microalgae, macroalgae, and aquatic plants and charophytes. In the microalgae group, aside from eutrophication, a few rarer causes were present, with their combined prevalence in the data amounting to just under 12%. For macroalgae, climate change (34%), and for aquatic plants, indirect ecological interactions (76%) and physical disturbance of the seabed (83%) were also notably prevalent in the data, alongside eutrophication. In the data concerning epifauna, environmental fluctuations were clearly the most common cause (100%) of negative biodiversity changes. For infauna, eutrophication was also the dominant cause (80%), while for fish, alongside eutrophication (57%), significant causes included climate change (53%) and human-induced direct mortality (30%). In the 'Multiple groups or ecosystems' category, eutrophication was the most significant cause (58%), while the importance of climate change was clearly lower (8%) compared to the entire dataset.

When analyzed by biotopes, the occurrence frequency of different drivers of biodiversity loss varied significantly across different habitat types in the data (Figure S7). Eutrophication was evident in the biodiversity loss data across all habitat-type groups and was the most common single cause in all groups. Eutrophication was identified as a driver of biodiversity loss in all hydrolittoral soft bottoms and mixed sediments, and in a large portion (83%) of infralittoral soft bottoms and mixed sediments, as well as hard bottoms and biogenic environments (94%). In addition to eutrophication, physical disturbance of the seabed and indirect ecological interactions were identified as drivers of loss in hydrolittoral soft bottoms and mixed sediments. Other significant causes of biodiversity loss in infralittoral soft bottoms and mixed sediments included climate change (9%), indirect ecological interactions (19%), and physical disturbance of the seabed (29%). Climate change was also a significant cause, along with eutrophication (33%), in the biodiversity loss of infralittoral hard bottoms and biogenic environments. In the littoral water mass, besides eutrophication, 'other' causes accounted for 40% of the biodiversity loss.

Geographically, the occurrence frequency of biodiversity loss causes in the data varied between sea areas (Figure S8). Eutrophication played a significant role (50%) in all marine areas except for the Bothnian Bay, with the greatest impact (>80%) observed in the southern marine areas. In contrast, the role of climate change was more prominent in the northern marine regions (>50%). In the Bothnian Bay, the most significant causes of biodiversity loss, based on prevalence in the data, were climate change and human-induced direct mortality (57% and 67%), while the significance of eutrophication was considerably lower than the overall average in the data (14%). In the Quark and the Bothnian Sea, the main causes of biodiversity loss were climate change (75% and 76%) and eutrophication (54% and 76%). In the sea areas of the Åland Islands, Archipelago Sea, and Gulf of Finland, eutrophication was clearly the most important cause, with a higher

prevalence than in the overall dataset (83–86% vs. 78%). In all of these areas, climate change was also identified as a common driver of biodiversity loss (>15%), and in the Gulf of Finland, indirect ecological interactions and physical disturbance of the seabed were also frequently recorded (28% and 25%).

Table S3. A list of included articles (tot. 90). A minus (–) sign after an article indicates that the article reports biodiversity loss, i.e., negative changes in some aspect of biodiversity. A plus (+) sign refers to data that could potentially have indicated negative changes in some aspect of biodiversity but did not; instead, they showed positive changes or no changes in the state of biodiversity.

(1)	Altartouri A, Nurminen L, Jolma A. 2014. Modeling the role of the close- range effect and environmental variables in the occurrence and spread of Phragmites australis in four sites on the Finnish coast of the Gulf of Finland and the Archipelago Sea. Ecology and Evolution 4:987–1005.	+
(2)	Aronsuu K, Huhmarniemi A. 2004. Changes in the European whitefish (*Coregonus lavaretus* (L.)) population of the Kalajoki — potential consequences of the alterations of fishing patterns in the Gulf of Bothnia. Annales Zoologici Fennici 41:195–204.	–
(3)	Bergström L, Heikinheimo O, Svirgsden R, Kruze E, Ložys L, Lappalainen A, Saks L, Minde A, Dainys J, Jakubavičiute E, Ådjers K, Olsson J. 2016. Long term changes in the status of coastal fish in the Baltic Sea. Estuarine, Coastal and Shelf Science 169:74–84.	–, +
(4)	Blomqvist E. 1984. Changes in fish community structure and migration activity in a brackish bay isolated by land upheaval and reverted by dredging. Ophelia Supplement 3:11–21.	–
(5)	Bonsdorff E, Aarnio K, Lindell A, Sandberg E. 1992. Long-term changes in the archipelago waters of Aland - a comparison of the zoobenthos 1972-90. Memoranda Societatis pro Fauna et Flora Fennica 68:1–9.	–, +
(6)	Bonsdorff E, Blomqvist E, Mattila J, Norkko A. 1997a. Long-term changes and coastal eutrophication. Examples from the Aland Islands and the Archipelago Sea, northern Baltic Sea. Oceanologica Acta 20:319–329.	–, +
(7)	Bonsdorff E, Blomqvist E, Mattila J, Norkko A. 1997b. Coastal eutrophication: Causes, consequences and perspectives in the Archipelago areas of the northern Baltic Sea. Estuarine Coastal and Shelf Science 44:63–72.	–, +
(8)	Boström C, Bonsdorff E, Kangas P, Norkko A. 2002. Long-term changes of a brackish-water eelgrass (*Zostera marina* L.) community indicate effects of coastal eutrophication. Estuarine Coastal and Shelf Science 55:795–804.	–, +
(9)	Candolin U, Voigt H-R. 2020. Population growth correlates with increased fecundity in three-spined stickleback populations in a human-disturbed environment. Aquatic Sciences 82:21.	+
(10)	Eveleens Maarse F, Salovius-Laurén S, Snickars M. 2020. Long-term changes in the phytobenthos of the southern Åland Islands, northern Baltic Sea. Nordic Journal of Botany 38: e02751	–, +
(11)	Fernandes JA, Kauppila P, Uusitalo L, Fleming-Lehtinen V, Kuikka S, Pitkänen H. 2012. Evaluation of reaching the targets of the water framework directive in the Gulf of Finland. Environmental Science and Technology 46:8220–8228.	+
(12)	Finni T, Laurila S, Laakkonen S. 2001. The history of eutrophication in the sea area of Helsinki in the 20th century - Long-term analysis of plankton assemblages. AMBIO 30:264–271.	–
(13)	Hänninen J, Vuorinen I. 2001. Macrozoobenthos structure in relation to environmental changes in the Archipelago Sea, northern Baltic Sea. Boreal Environmental Research 6:93–105.	+
(14)	Heikinheimo O, Pekcan-Hekim Z, Raitaniemi J. 2014. Spawning stock-recruitment relationship in pikeperch *Sander lucioperca* (L.) in the Baltic Sea, with temperature as an environmental effect. Fisheries Research 155:1–9.	+
(15)	Holmström N, Haahtela I, Bonsdorff E. 2007. A new reality for coastal zoobenthos: Long-term changes (1958–2005) in a shallow sheltered bay. Memoranda Societatis pro Fauna et Flora Fennica 83:1–8.	–, +
(16)	Honkanen T, Helminen H. 2000. Impacts of fish farming on eutrophication: Comparisons among different characteristics of ecosystem. International Review of Hydrobiology 85:673–686.	+
(17)	Hudd R, Leskelä A. 1998. Acidification-induced species shifts in coastal fisheries off the River Kyrönjoki, Finland: A case study. AMBIO 27:535–538.	–, +
(18)	Hudd R, Kjellman J, Urho L. 1996. The increase of coincidence in relative year-class strengths of coastal perch (*Perca fluviatilis* L.) stocks in the Baltic Sea. Annales Zoologici Fennici 33:383–387.	+
(19)	Ilus E, Keskitalo J. 2008. The response of phytoplankton to increased temperature in the Loviisa archipelago, Gulf of Finland. Boreal Environment Research 13:503–516.	–, +

(20) Jaatinen K, Westerbom M, Norkko A, Mustonen O, Koons DN. 2021. Detrimental impacts of climate change may be exacerbated by density-dependent population regulation in blue mussels. Journal of Animal Ecology 90:562–573. +

(21) Jokinen H, Wennhage H, Ollus V, Aro E, Norkko A. 2016. Juvenile flatfish in the northern Baltic Sea – long-term decline and potential links to habitat characteristics. Journal of Sea Research 107:67–75. –

(22) Jokinen H, Wennhage H, Lappalainen A, Ådjers K, Rask M, Norkko A. 2015. Decline of flounder (*Platichthys flesus* (L.)) at the margin of the species' distribution range. Journal of Sea Research 105:1–9. –

(23) Jutila E, Saura A, Kallio-Nyberg I, Huhmarniemi A, Romakkaniemi A. 2007. The status and exploitation of sea trout on the Finnish coast of the Gulf of Bothnia in the Baltic Sea. Teoksessa: Harris G, Milner N (Toim.). Sea Trout: Biology, Conservation and Management. Blackwell Publishing Ltd. pp. 128–138. –

(24) Kääriä J, Eklund J, Hallikainen S, Kääriä R, Rajasilta M, Ranta-aho K, Soikkeli M. 1988. Effects of coastal eutrophication on the spawning grounds of the Baltic herring in the SW Archipelago of Finland. Kieler Meeresforschungen - Sonderheft 6:348–356. –

(25) Kangas P, Autio H, Hällfors G, Luther H, Niemi A, Salemaa H. 1982. A general model of the decline of *Fucus vesiculosus* at Tvärminne, south coast of Finland in 1977-81 (Baltic Sea). Acta Botanica Fennica 118:1–27. –

(26) Keskitalo J. 1987. Phytoplankton in the sea area off the Olkiluoto nuclear power station, west coast of Finland. Annales Botanici Fennici 24:281–299. –, +

(27) Kjellman J, Hudd R. 1996. Changed length-at-age of burbot, *Lota lota*, from an acidified estuary in the Gulf of Bothnia. Environmental Biology of Fishes 45:65–73. –, +

(28) Kokkonen E, Vainikka A, Heikinheimo O. 2015. Probabilistic maturation reaction norm trends reveal decreased size and age at maturation in an intensively harvested stock of pikeperch *Sander lucioperca*. Fisheries Research 167:1–12. –, +

(29) Kokkonen E, Heikinheimo O, Pekcan-Hekim Z, Vainikka A. 2019. Effects of water temperature and pikeperch (*Sander lucioperca*) abundance on the stock–recruitment relationship of Eurasian perch (*Perca fluviatilis*) in the northern Baltic Sea. Hydrobiologia 841:79–94. –, +

(30) Korhola A, Blom T. 1996. Marked early 20th century pollution and the subsequent recovery of Töölö Bay, central Helsinki, as indicated by subfossil diatom assemblage changes. Hydrobiologia 341:169–179. –, +

(31) Kraufvelin P, Sinisalo B, Leppäkoski E, Mattila J, Bonsdorff E. 2001. Changes in zoobenthic community structure after pollution abatement from fish farms in the Archipelago Sea (N. Baltic Sea). Marine Environmental Research 51:229–245. –, +

(32) Laine AO, Luodekari K, Poikonen M, Viitasalo M. 2003. A comparison between 1928 and 2000 indicates major changes in the macrozoobenthos species composition and abundance on the SW coast of Finland (Baltic Sea). Proceedings of the Estonian Academy of Sciences. Biology. Ecology 52:3. –, +

(33) Lappalainen A, Pesonen L. 2000. Changes in fish community structure after cessation of waste water discharge in a coastal bay area west of Helsinki, Northern Baltic Sea. Archive of Fishery and Marine Research 48:226–241. –, +

(34) Lappalainen A, Söderkultalahti P, Wiik T. 2002. Changes in the commercial fishery for pikeperch (*Stizostedion lucioperca*) on the Finnish coast from 1980 to 1999 – Consequences of environmental and economic factors. Archive of Fishery and Marine Research 49:199–212. +

(35) Lappalainen A, Rask M, Koponen H, Vesala S. 2001. Relative abundance, diet and growth of perch (*Perca fluviatilis*) and roach (*Rutilus rutilus*) at Tvärminne, northern Baltic Sea, in 1975 and 1997: Responses to eutrophication? Boreal Environment Research 6:107–118. –

(36) Lappalainen A, Hyvönen J, Söderkultalahti P, Heikkinen J. 2020. Estimating annual cpue indices for perch (*Perca fluviatilis*) from monthly logbook data of a gill-net fishery in the Bothnian Bay, Baltic Sea. Boreal Environment Research 25:79–91. –, +

(37) Lappalainen A, Saks L, Sustar M, Heikinheimo O, Juergens K, Kokkonen E, Kurkilahti M, Verliin A, Vetemaa M. 2016. Length at maturity as a potential indicator of fishing pressure effects on coastal pikeperch (*Sander lucioperca*) stocks in the northern Baltic Sea. Fisheries Research 174:47–57. +

(38) Lappalainen J, Lehtonen H. 1995. Year-class strength of pikeperch (*Stizostedion lucioperca* L) in relation to environmental factors in a shallow Baltic Bay. Annales Zoologici Fennici 32:411–419. +

(39) Lehikoinen A, Heikinheimo O, Lappalainen A. 2011. Temporal changes in the diet of great cormorant (*Phalacrocorax carbo sinensis*) on the southern coast of Finland – Comparison with available fish data. Boreal Environment Research 16:61–70. –, +

(40) Lehikoinen A, Heikinheimo O, Lehtonen H, Rusanen P. 2017. The role of cormorants, fishing effort and temperature on the catches per unit effort of fisheries in Finnish coastal areas. Fisheries Research 190:175–182. +

(41)	Lehtonen H. Jokikokko E. 1995. Changes in the heavily exploited vendace (*Coregonus albula* L) stock in the northern Bothnian Bay. Advances in Limnology 46:379–386.	–, +
(42)	Lehtonen H, Urho L, Kjellman J. 1998. Responses of ruffe (*Gymnocephalus cernuus* (L.)) abundance to eutrophication. Journal of Great Lakes Research 24:285–292.	–, +
(43)	Lehtonen H, Leskinen E, Selen R, Reinikainen M. 2009. Potential reasons for the changes in the abundance of pike, *Esox lucius*, in the western Gulf of Finland, 1939–2007. Fisheries Management and Ecology 16:484–491.	–, +
(44)	Leonardsson K, Hudd R, Veneranta L, Huhmarniemi A, Jokikokko E. 2016. Optimal time and sample allocation for unicohort fish larvae, sea-spawning whitefish (*Coregonus lavaretus* s. l.) as a case study. Ices Journal of Marine Science 73:374–383.	+
(45)	Leppäkoski E, Helminen H, Hanninen J, Tallqvist M. 1999. Aquatic biodiversity under anthropogenic stress: an insight from the Archipelago Sea (SW Finland). Biodiversity and Conservation 8:55–70.	–
(46)	Mattila J. 1993. Long-term changes in the bottom fauna along the Finnish coast of the southern Bothnian Sea. Aqua Fennica 23:143–152.	–, +
(47)	McCairns RJS, Kuparinen A, Panda B, Jokikokko E, Merilä J. 2012. Effective size and genetic composition of two exploited, migratory whitefish (*Coregonus lavaretus lavaretus*) populations. Conservation Genetics 13:1509–1520.	–, +
(48)	Momigliano P, Jokinen H, Calboli F, Aro E, Merilä J. 2019. Cryptic temporal changes in stock composition explain the decline of a flounder (*Platichthys* spp.) assemblage. Evolutionary Applications 12:549–559.	–
(49)	Munsterhjelm R, Henricson C, Sandberg-Kilpi E. 2008. The decline of a charophyte – occurrence dynamics of *Chara tomentosa* L. at the southern coast of Finland. Memoranda Societatis pro Fauna et Flora Fennica 84:56–80.	–
(50)	Mustamaki N, Mattila J. 2015. Structural changes in three coastal fish assemblages in the northern Baltic Sea archipelago. Estuarine Coastal and Shelf Science 164:408–417.	–, +
(51)	Mustamäki N, Bergström U, Ådjers K, Sevastik A, Mattila J. 2014. Pikeperch (*Sander lucioperca* (L.)) in decline: High Mortality of Three Populations in the Northern Baltic Sea. AMBIO 43:325–336.	–, +
(52)	Olli K, Nyman E, Tamminen T. 2023. Half-century trends in alpha and beta diversity of phytoplankton summer communities in the Helsinki Archipelago, the Baltic Sea. Journal Of Plankton Research 45:146–162.	+
(53)	Olsson J, Andersson ML, Bergström U, Arlinghaus R, Audzijonyte A, Berg S, Briekmane L, Dainys J, Ravn HD, Droll J, Dziemian Ł, Fey DP, Van Gemert R, Greszkiewicz M, Grochowski A, Jakubavičiūtė E, Lozys L, Lejk AM, Mustamäki N, Naddafi R, Olin M, Saks L, Skov C, Smoliński S, Svirgsden R, Tiainen J, Östman Ö. 2023. A pan-Baltic assessment of temporal trends in coastal pike populations. Fisheries Research 260:106594.	–, +
(54)	Packalén A, Korpinen S, Lehtonen KK. 2008. The invasive amphipod species *Gammarus tigrinus* (Sexton, 1939) can rapidly change littoral communities in the Gulf of Finland (Baltic Sea). Aquatic Invasions 3:405–412.	–
(55)	Palmqvist G, von Numers M. 2006. Changes in the flora during a half-century in the central part of the Archipelago Sea, SW Finland. Memoranda Societatis pro Fauna et Flora Fennica 82:10–23.	+
(56)	Parmanne R, Lindström K. 2003. Annual variation in gobiid larval density in the northern Baltic Sea. Journal of Fish Biology 62:413–426.	+
(57)	Pekcan-Hekim Z, Urho L, Auvinen H, Heikinheimo O, Lappalainen J, Raitaniemi J, Söderkultalahti P. 2011. Climate warming and pikeperch year-class catches in the Baltic Sea. AMBIO 40:447–456.	+
(58)	Peltonen H, Weigel B. 2022. Responses of coastal fishery resources to rapid environmental changes. Journal Of Fish Biology 101:686–698.	–, +
(59)	Perus J, Bonsdorff E. 2004. Long-term changes in macrozoobenthos in the Åland archipelago, northern Baltic Sea. Journal of Sea Research 52:45–56.	–, +
(60)	Pitkänen H, Peuraniemi M, Westerbom M, Kilpi M, von Numers M. 2013. Long-term changes in distribution and frequency of aquatic vascular plants and charophytes in an estuary in the Baltic Sea. Annales Botanici Fennici 50:1–54.	–, +
(61)	Rajasilta M, Mankki J, Ranta-Aho K, Vuorinen I. 1999. Littoral fish communities in the Archipelago Sea, SW Finland: a preliminary study of changes over 20 years. Hydrobiologia 393:253–260.	–
(62)	Rajasilta M, Mäkinen K, Ruuskanen S, Hänninen J, Laine P. 2021. Long-term data reveal the associations of the egg quality with abiotic factors and female traits in the Baltic herring under variable environmental conditions. Frontiers in Marine Science 8:698480.	–
(63)	Rajasilta M, Elfving M, Hänninen J, Laine P, Vuorinen I, Paranko J. 2016. Morphological abnormalities in gonads of the Baltic herring (*Clupea harengus mem*bras): Description of types and prevalence in the northern Baltic Sea. AMBIO 45:205–214.	–, +

(64) Rajasilta M, Hänninen J, Laaksonen L, Laine P, Suomela J-P, Vuorinen I, Mäkinen K. 2019. Influence of environmental conditions, population density, and prey type on the lipid content in Baltic herring (*Clupea harengus membras*) from the northern Baltic Sea. Canadian Journal of Fisheries and Aquatic Sciences 76:576–585. –

(65) Reuss N, Conley D, Bianchi T. 2005. Preservation conditions and the use of sediment pigments as a tool for recent ecological reconstruction in four Northern European estuaries. Marine Chemistry 95:283–302. +

(66) Rinne H, Salovius-Laurén S. 2020. The status of brown macroalgae Fucus spp. and its relation to environmental variation in the Finnish marine area, northern Baltic Sea. Ambio 49:118–129. –, +

(67) Rinne H, Kostamo K. 2022. Distribution and species composition of red algal communities in the northern Baltic Sea. Estuarine Coastal and Shelf Science 269:107806. –, +

(68) Rönnberg C, Bonsdorff E. 2004. Baltic Sea eutrophication: area-specific ecological consequences. Hydrobiologia 514:227–241. –, +

(69) Rönnberg O, Mathiesen L. 1998. Long-term changes in the marine macroalgae of Lågskär, Åland Sea (N Baltic). Nordic Journal of Botany 18:379–384. –, +

(70) Rönnberg O, Lehto J, Haahtela I. 1985. Recent changes in the occurrence of *Fucus vesiculosus* in the Archipelago Sea, SW Finland. Annales Botanici Fennici 22:231–244. –, +

(71) Roos C, Rönnberg O, Berglund J, Alm A. 2004. Long-term changes in macroalgal communities along ferry routes in a northern Baltic archipelago. Nordic Journal of Botany 23:247–259. –, +

(72) Sahla M, Tolvanen H, Ruuskanen A, Kurvinen L. 2020. Assessing long term change of *Fucus* spp. communities in the northern Baltic Sea using monitoring data and spatial modeling. Estuarine, Coastal and Shelf Science 245:107023. –

(73) Snickars M, Weigel B, Bonsdorff E. 2015. Impact of eutrophication and climate change on fish and zoobenthos in coastal waters of the Baltic Sea. Marine Biology 162:141–151. –, +

(74) Snickars M, Rinne H, Salovius-Laurén S, Arponen H, O'Brien K. 2014. Disparity in the occurrence of *Fucus vesiculosus* in two adjacent areas of the Baltic Sea — current status and outlook for the future. Boreal Environment Research 19:441–451. –, +

(75) Torn K, Krause-Jensen D, Martin G. 2006. Present and past depth distribution of bladderwrack (*Fucus vesiculosus*) in the Baltic Sea. Aquatic Botany 84:53–62. –

(76) Vahteri P, Vuorinen I. 2016. Continued decline of the bladderwrack, *Fucus vesiculosus*, in the Archipelago Sea, northern Baltic proper. Boreal Environment Research 21:373–386. –, +

(77) Veneranta L, Hudd R, Vanhatalo J. 2013. Reproduction areas of sea-spawning coregonids reflect the environment in shallow coastal waters. Marine Ecology Progress Series 477:231–250. –, +

(78) Veneranta L, Kallio-Nyberg I, Saloniemi I, Jokikokko E, Nash AER. 2021. Changes in age and maturity of anadromous whitefish (*Coregonus lavaretus*) in the northern Baltic Sea from 1998 to 2014. Aquatic Living Resources 34:9. –, +

(79) Viitasalo M, Bonsdorff E. 2022. Global climate change and the Baltic Sea ecosystem: direct and indirect effects on species, communities and ecosystem functioning. Earth System Dynamics 13:711–747. –

(80) Villnäs A, Perus J, Bonsdorff E. 2011. Structural and functional shifts in zoobenthos induced by organic enrichment - Implications for community recovery potential. Journal Of Sea Research 65:8–18. –, +

(81) Virtanen EA, Kallio N, Nurmi M, Jernberg S, Saikkonen L, Forsblom L. 2023. Recreational land use contributes to the loss of marine biodiversity. People and Nature pan3:10444. –

(82) von Numers M, Korvenpää T. 2007. 20th Century vegetation changes in an island archipelago, SW Finland. Ecography 30:789–800. +

(83) Weckström K. 2006. Assessing recent eutrophication in coastal waters of the Gulf of Finland (Baltic Sea) using subfossil diatoms. Journal of Paleolimnology 35:571–592. –, +

(84) Weckström K, Korhola A, Weckström J. 2007. Impacts of eutrophication on diatom life forms and species richness in coastal waters of the Baltic Sea. AMBIO 36:155–160. –

(85) Weigel B, Blenckner T, Bonsdorff E. 2016. Maintained functional diversity in benthic communities in spite of diverging functional identities. OIKOS 125:1421–1433. –, +

(86) Weigel B, Mäkinen J, Kallasvuo M, Vanhatalo J. 2021. Exposing changing phenology of fish larvae by modeling climate effects on temporal early life-stage shifts. Marine Ecology Progress Series 666:135–148. –

(87) Weigel B, Andersson HC, Meier HEM, Blenckner T, Snickars M, Bonsdorff E. 2015. Long-term progression and drivers of coastal zoobenthos in a changing system. Marine Ecology Progress Series 528:141–159. –, +

(88)	Westerbom M, Mustonen O, Jaatinen K, Kilpi M, Norkko A. 2019. Population dynamics at the range margin: implications of climate change on sublittoral blue mussels (*Mytilus trossulus*). Frontiers in Marine Science 6:292.	–, +
(89)	Yletyinen J, Bodin Ö, Weigel B, Nordström MC, Bonsdorff E, Blenckner T. 2016. Regime shifts in marine communities: a complex systems perspective on food web dynamics. Proceedings of the Royal Society B: Biological Sciences 283:20152569.	–
(90)	Ådjers K, Appelberg M, Eschbaum, R, Lappalainen A, Minde A, Repečka R, Thoresson G. 2006. Trends in coastal fish stocks of the Baltic Sea. Boreal Environment Research 11.13–25.	–, +

Table S4. Articles and research observations included in the report, listed by scientific journal.

**Scientific journal**	**Articles (nr)**	**Observations (nr)**
Acta Botanica Fennica	1	1
Advances in Limnology	2	5
AMBIO	7	23
Annales Botanici Fennici	3	70
Annales Zoologici Fennici	3	5
Aqua Fennica	1	5
Aquatic Botany	1	3
Aquatic Invasions	1	1
Aquatic Living Resources	1	20
Aquatic Sciences	1	2
Archive of Fishery and Marine Research	2	4
Biodiversity and Conservation	1	2
Boreal Environment Research	8	35
Canadian Journal of Fisheries and Aquatic Sciences	1	1
Sea Trout: Biology, Conservation and Management (book)	1	3
Concervation Genetics	1	5
Earth System Dynamics	1	1
Ecography	1	4
Ecology and Evolution	1	4
Environmental Biology of Fishes	1	7
Environmental Science and Technology	1	2
Estuarine, Coastal and Shelf Science	6	103
Evolutionary Applications	1	1
Fisheries Management and Ecology	1	2
Fisheries Research	5	17
Frontiers in Marine Science	2	14
Hydrobiologia	4	37
ICES Journal of Marine Science	1	2
International Review of Hydrobiology	1	3
Journal of Animal Ecology	1	1
Journal of Fish Biology	2	93
Journal of Great Lakes Research	1	6
Journal of Paleolimnology	1	10
Journal of Plankton Research	1	1
Journal of Sea Research	4	30
Kieler Meeresforschungen - Sonderheft	1	1
Marine Biology	1	2
Marine Chemistry	1	1
Marine Ecology Progress Series	3	35
Marine Environmental Research	1	13
Memoranda - Societatis pro Fauna et Flora Fennica	3	20
Nordic Journal of Botany	3	142
Oceanologica Acta	1	6

OIKOS	1	2
Ophelia	1	1
People and Nature	1	4
Proceedings of the Estonian Academy of Sciences	1	19
Proceedings of the Royal Society B: Biological Sciences	1	5

Table S5. Species or taxa addressed in the biodiversity loss datasets, organized by organism groups. The list includes the taxonomic names found in each dataset, and therefore, it may contain some outdated names as well as different names for the same species.

Microalgae
Bacillariophyceae
Peridinella catenata
Calycomonas sp.
Cyclotella sp.
Synedra acus
Synedra pulchella

Macroalgae
Aglaothamnion roseum
Ahnfeltia plicata
Audouinella efflorescens
Battersia plumigera
Capsosiphon fulvescens
Ceramium tenuicorne
Chaetomorpha linum
Chaetomorpha sp.
Chorda filum
Chroodactylon ornatum
Cladophora rupestris
Coccotylus truncatus
Dictyosiphon chordaria
Dictyosiphon foeniculaceus
Ectocarpus siliculosus
Enteromorpha sp.
Erythrocladia polystromatica
Eudesme virescens
Fucus vesiculosus
Fucus spp.
Furcellaria lumbricalis
Gaillona rosea
Grania efflorescens
Halopteris scoparia
Litosiphon laminariae
Phyllophora pseudoceranoides
Pilayella littoralis
Polysiphonia fibrillosa
Polysiphonia fucoides
Pseudolithoderma rosenvingei
Rhodochorton purpureum
Rhodomela confervoides
Sphacelaria arctica
Sphacelaria plumigera
Sphacelaria spp.
Spongomorpha aeruginosa
Stictyosiphon tortilis
Streblonema oligosporum
Ulva intestinalis
Urospora penicilliformis
Vertebrata fucoides

Aquatic plants
Alisma plantago-aquatica
Chara aspera
Chara baltica
Chara globularis
Chara tomentosa
Elatine hydropiper
Elatine triandra
Eleocharis acicularis
Eleocharis parvula
Equisetum fluviatile
Isoëtes echinospora
Isoëtes lacustris
Myriophyllum alterniflorum
Nitella sp.
Nymphaea alba
Persicaria amphibia
Plantago uniflora
Potamogeton alpinus
Potamogeton gramineus
Potamogeton pectinatus
Potamogeton perfoliatus
Potamogeton pusillus
Ranunculus confervoides
Ranunculus peltatus
Ranunculus reptans
Sagittaria sagittifolia
Schoenoplectus maritimus
Schoenoplectus sp.
Stuckenia filiformis
Tolypella nidifica
Typha angustifolia
Typha latifolia

Epifauna
Gammarus spp.
Mytilus edulis
Mytilus trossulus

Infauna
Asellus asellus
Bithynia tentaculata
Bylgides sarsi
Cerastoderma glaucum
Ceratopogonidae
Chironomidae
Corophium volutator
Gammarus locusta
Halicryptus spinulosus
Harmothoe sarsi
Hydrobia sp.
Jaera albifrons
Lymnea peregra
Macoma balthica
Monoporeia affinis
Mya arenaria
Nereis diversicolor
Oligochaeta
Polydora redeki
Pontoporeia affinis
Prostoma obscurum
Pygospio elegans
Saduria entomon
Theodoxus fluviatilis
Trichoptera

Fish
Abramis brama
Alburnus alburnus
Blicca bjoerkna
Clupea harengus
Coregonus albula
Coregonus lavaretus
Coregonus maraena
Coregonus sp.
Esox lucius
Gasterosteus aculeatus
Gobius niger
Gobiusculus flavescens
Gymnocephalus cernuus
Lota lota

Nerophis ophidion	*Platichthys* sp.	*Salmo trutta*
Osmerus eperlanus	*Pomatoschistus minutus*	*Sander lucioperca*
Perca fluviatilis	*Pungitius pungitius*	*Zoarces viviparus*
Phoxinus phoxinus	*Rutilus rutilus*	

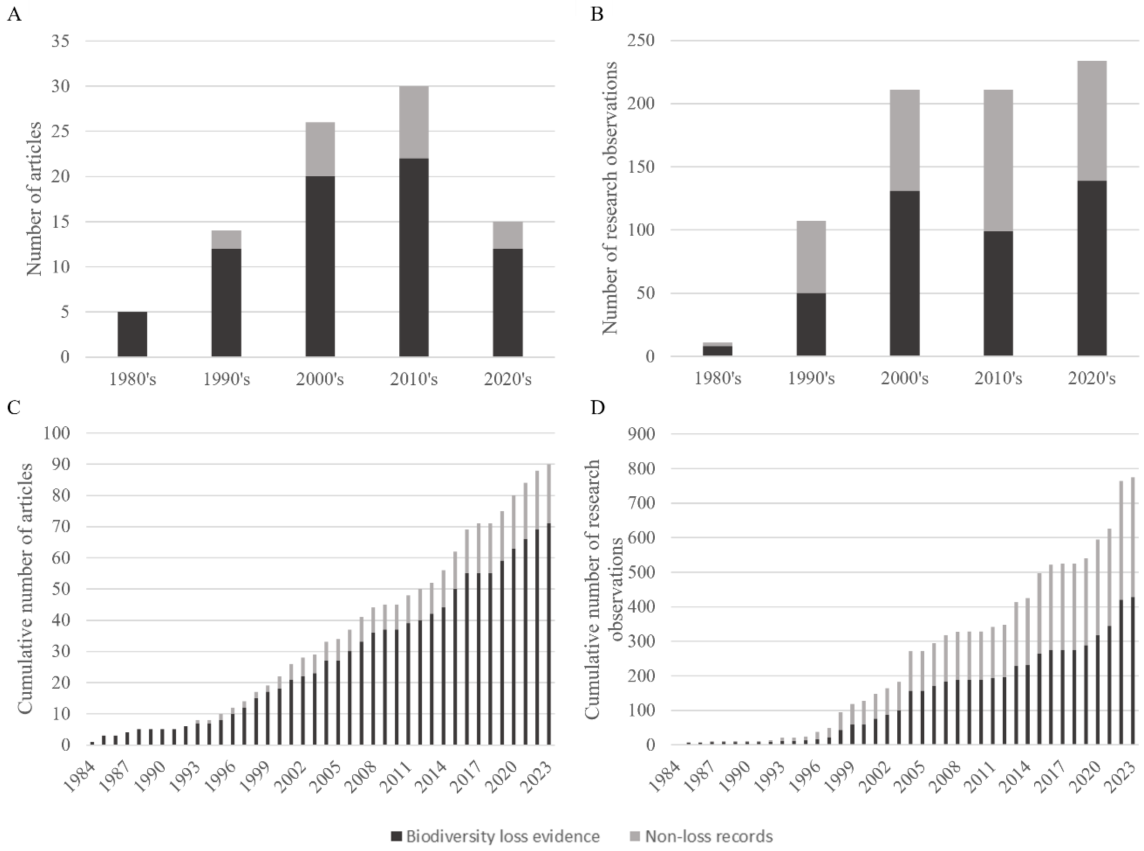

Figure S1. The absolute and cumulative numbers of included articles (A & C; n = 90) and research observations (B & D; n = 774) are presented by publication decade (A & B) and publication year (C & D). The share of biodiversity loss evidence in the entire dataset is shown in a darker shade whereas the records showing no such evidence is shown in a lighter shade.

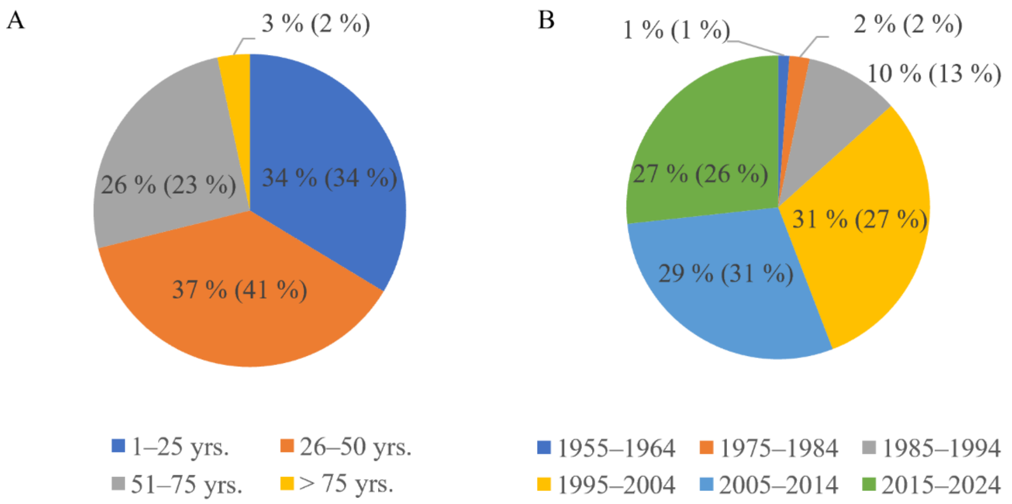

Figure S2. The distribution of research observations indicating biodiversity loss according to their temporal coverage in 25-year periods (A, n = 419) and according to the ending years of the data in ten-year intervals (B, n = 427). The percentages in parentheses reflect the corresponding distribution of the whole included research observation data set (n = 774).

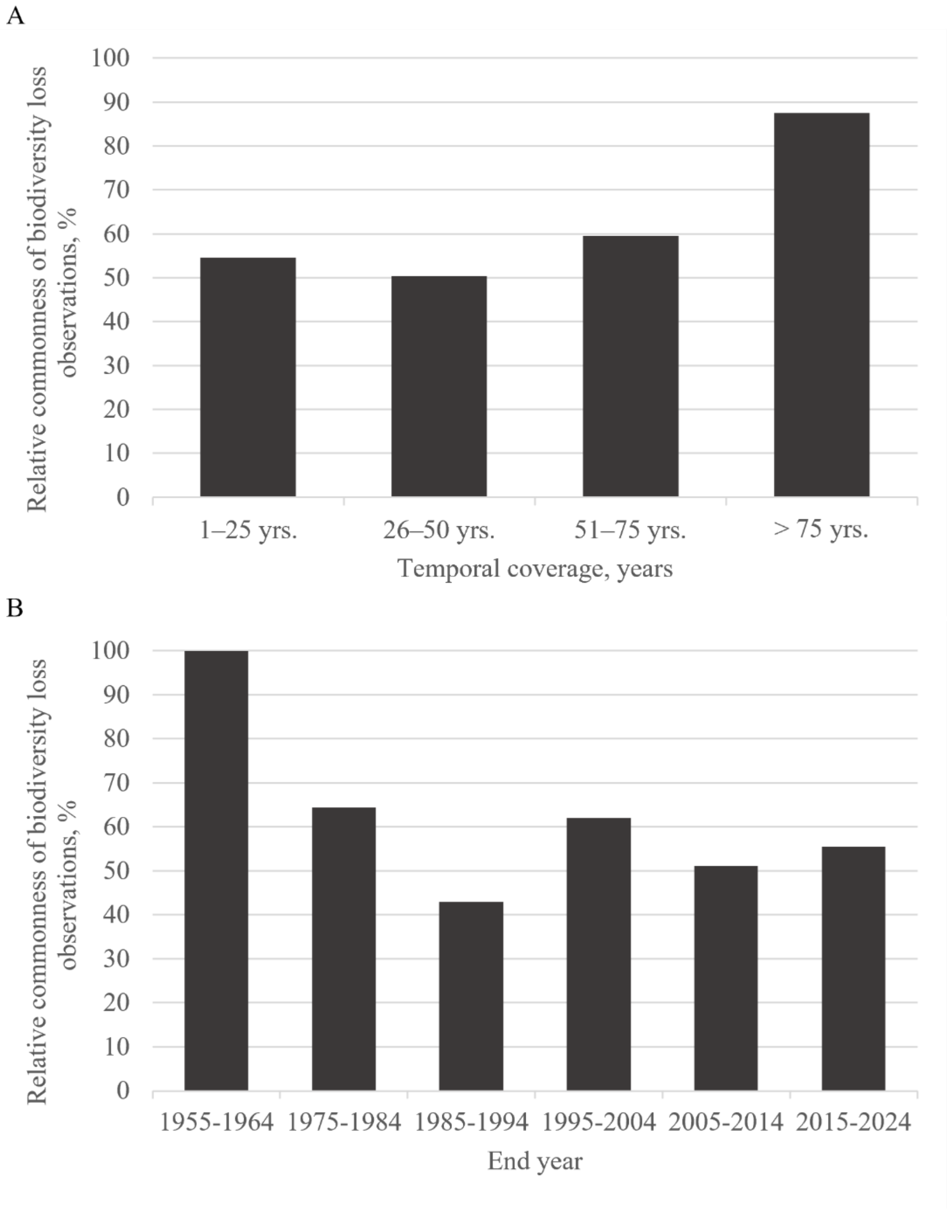

Figure S3. The relative occurrence of biodiversity loss observations in the included dataset displayed by (A) temporal coverage in 25-year intervals and by (B) the end year of change observations grouped into ten-year intervals.

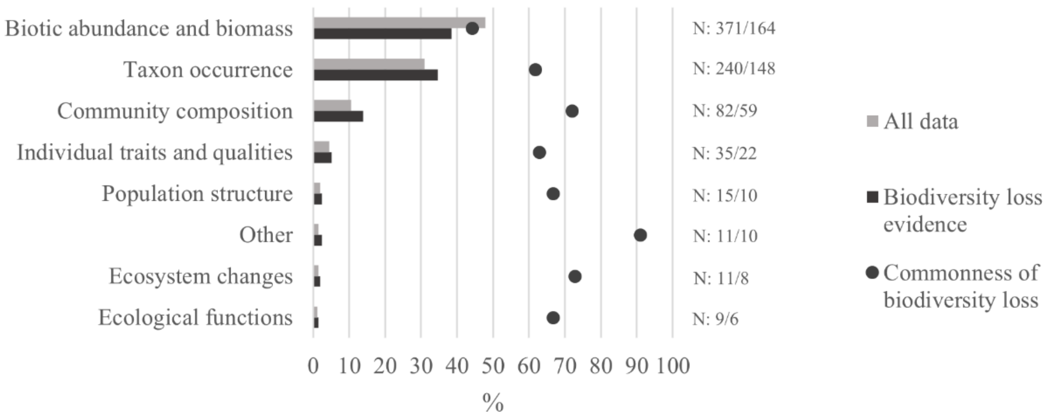

Figure S4. The percent distribution of data records by different categories of biodiversity. The share of all included data (n = 774) is shown in a lighter shade and the biodiversity loss evidence (n = 427) in a darker shade. The Commonness of biodiversity loss (= relative occurrence), as percentage of the biodiversity loss evidence of all research observations in each of the categories, is indicated as black filled circles. Number of observations in each category is indicated on the right side of the graphs ("N: all observations/biodiversity loss observations").

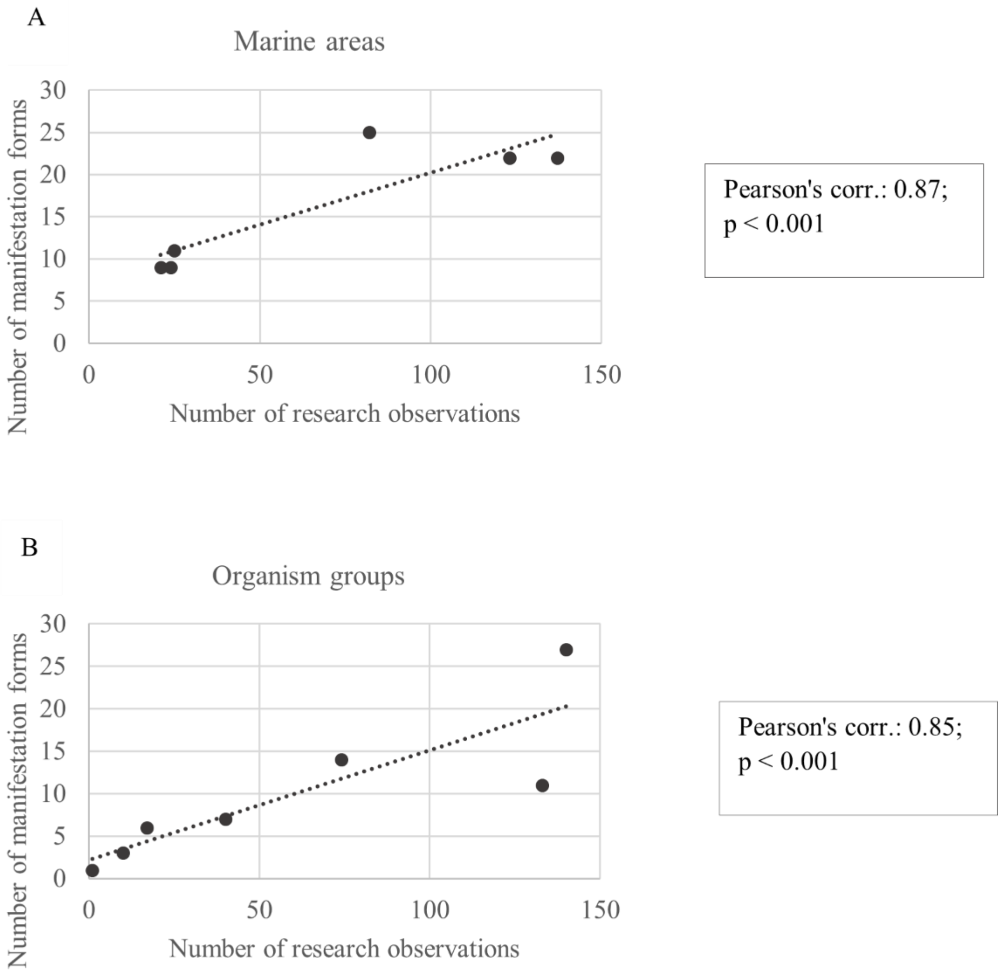

Figure S5. The relationship between the diversity of expression forms of biodiversity loss and the amount of biodiversity loss data, examined by marine area and organism group. There is a clear correlation between the number of expression forms and the number of research observations both regarding marine areas and organism groups (Pearson's correlation: 0.85–0.87; $p < 0.001$).

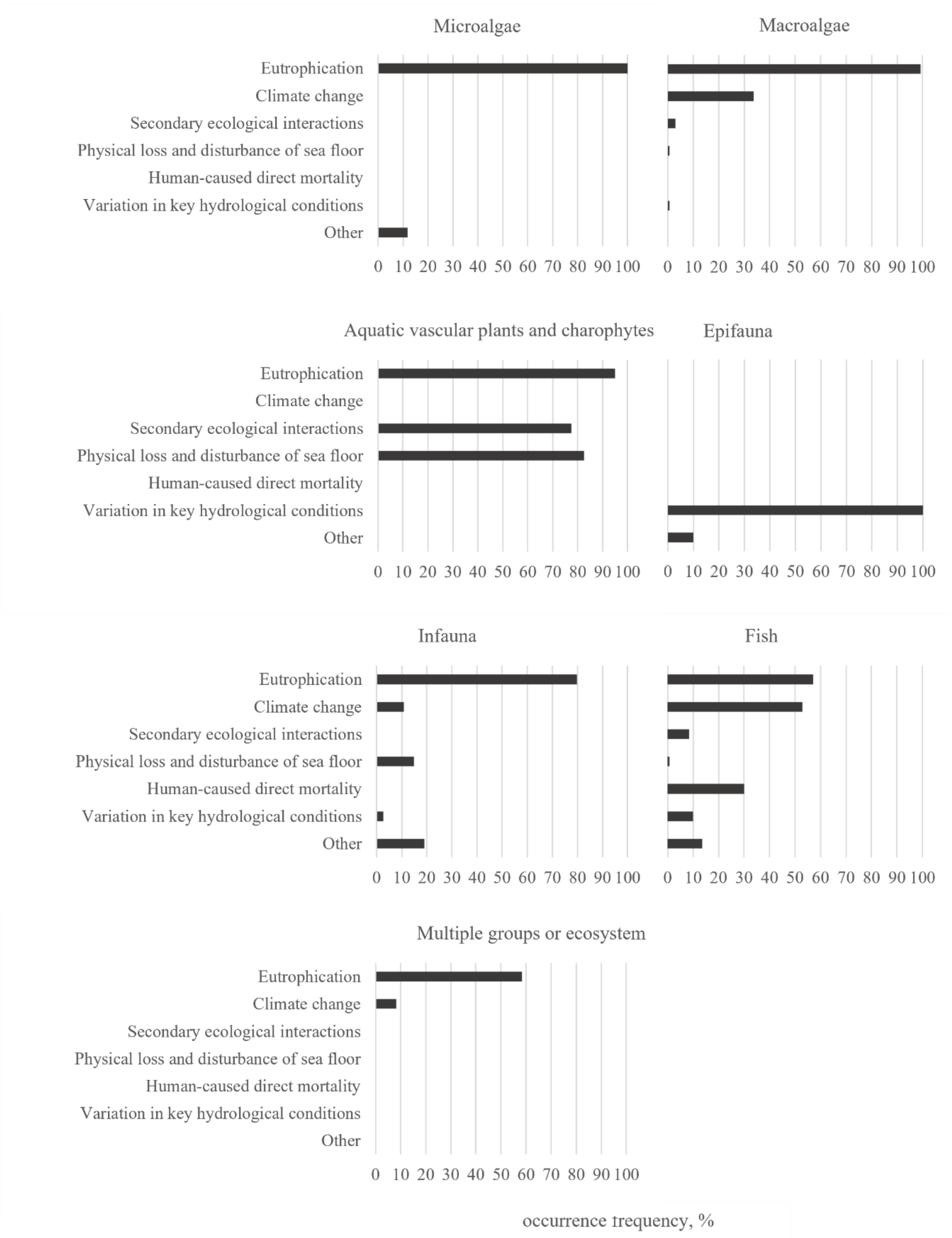

Figure S6. The occurrence frequency (%) of different drivers of biodiversity loss in the biodiversity loss evidence dataset presented for different littoral organism groups. Each research observation indicating biodiversity loss may be associated with one or multiple loss drivers that all were counted separately, attaining a possible occurrence frequency between 0–100%. The "Other" category includes the following seven drivers with less than 5% occurrence frequency: reduction of eutrophication, loss or deterioration of habitats, invasive species, acidification of water, artificial warming of water, species stocking of fish, and harmful substances. The number of research observations in different organism groups was:

Microalgae, N = 17; Macroalgae, N = 133; Aquatic vascular plants and charophytes, N = 40; Epifauna, N = 10; Infauna, N = 74; Fish, N = 138; Multiple groups or ecosystem, N = 8. Zooplankton not shown.

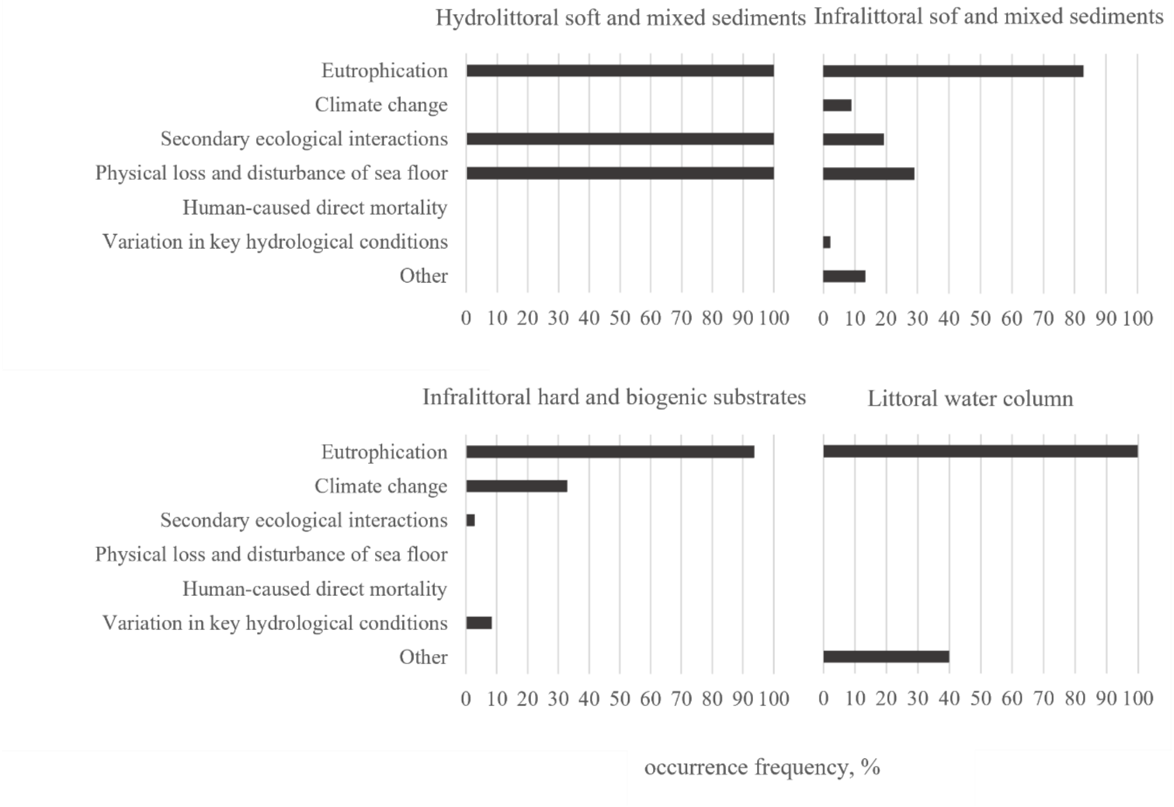

Figure S7. The occurrence frequency (%) of different drivers of biodiversity loss in the biodiversity loss evidence dataset presented for different littoral biotopes. Each research observation indicating biodiversity loss may be associated with one or multiple loss drivers that all were counted separately, attaining a possible occurrence frequency between 0–100%. The "Other" category includes the following seven drivers with less than 5% occurrence frequency: reduction of eutrophication, loss or deterioration of habitats, invasive species, acidification of water, artificial warming of water, species stocking of fish, and harmful substances. The number of research observations in different biotope categories was: Hydrolittoral soft and mixed sediments, N = 5; Infralittoral soft and mixed sediments, N = 134; Infralittoral hard and biogenic substrates, N = 143; Littoral water column, N = 5.

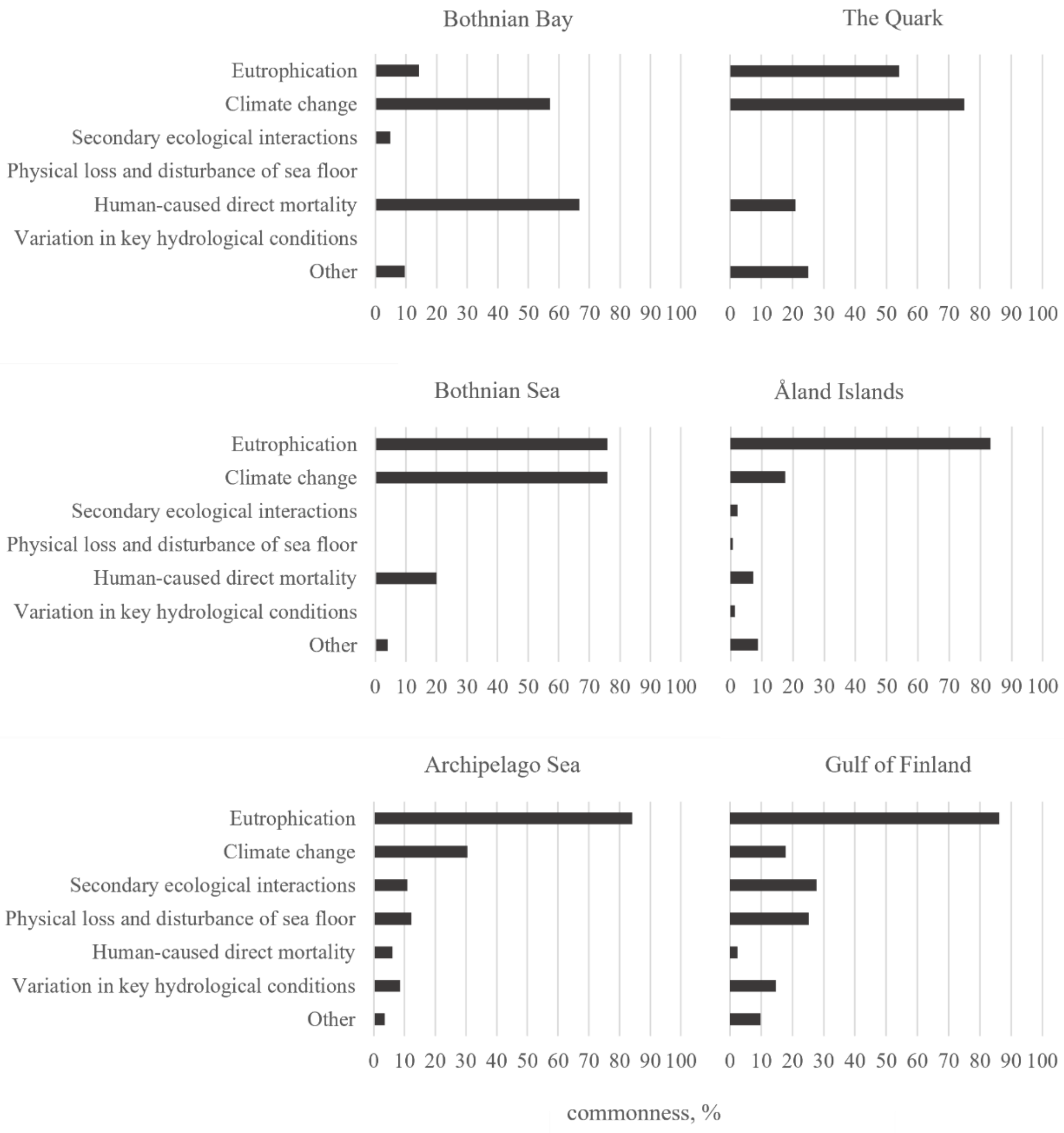

Figure S8. The occurrence frequency (%) of different drivers of biodiversity loss in the biodiversity loss evidence dataset presented for different geographical areas. Each research observation indicating biodiversity loss may be associated with one or multiple loss drivers that all were counted separately, attaining a possible occurrence frequency between 0–100%. The "Other" category includes the following seven drivers with less than 5% occurrence frequency: reduction of eutrophication, loss or deterioration of habitats, invasive species, acidification of water, artificial warming of water, species stocking of fish, and harmful substances. The number of research observations in different organism groups was: Bothnian Bay, N = 21; The Quark, N = 24; Bothnian Sea, N = 25; sea areas of the Åland Islands, N = 137; Archipelago Sea, N = 82; Gulf of Finland, N = 123; Multiple groups or ecosystem, N = 8.
